# Supplementary material for: Electrochemical cells from water ice? Preliminary methods and results
Source: PLoS One. 2023 Aug 24;18(8):e0285507. doi: 10.1371/journal.pone.0285507 (PMC10449211; doi:10.1371/journal.pone.0285507)
Supplement: S1 File — (DOC) [file pone.0285507.s001.doc]

Electrochemical cells from water ice? Preliminary methods and results

*Daniel S. Helman, Ph.D.*

Education Division, College of Micronesia-FSM, Yap Campus, Federated States of Micronesia

dhelman@comfsm.fm; danielhelmanteaching@yahoo.com

*Matthew Retallack, Ph.D.*

Carleton University, Canada

MatthewRetallack@cmail.carleton.ca

Supplemental Information

Table of Contents

[S1 Original Experimental Plans](#S1) and Methods

[S2 Detailed Descriptions of Results](#S2)

[S3 Experimental Failures and Faults](#S3)

[S4 Geomagnetic Correlation with Sunrise and Sunset](#S4)

[S5 Astrobiology and the Origin of Life](#S5)

[References](#References)

Section S1 Original Experimental Plans

Figure S1 shows two model schematics of how prototypes were planned for this project. Experiments 1-4 used the two-layer prototypes model, with p- and n- type layers, as with traditional photovoltaics. Experiments 5-6 used prototypes of electrochemical cells with an intermediate acid or base layer between the two ice layers. We called these two models the Semiconductor Model and the Electrochemical Model, respectively.


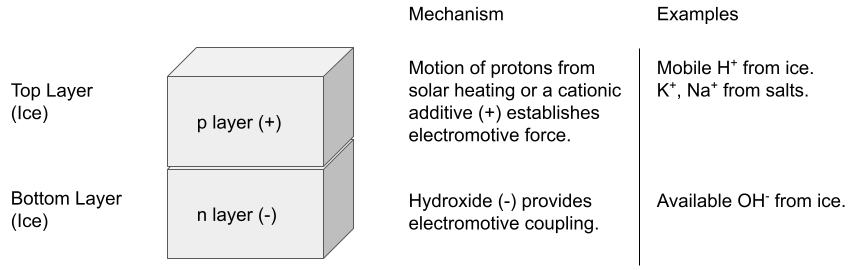


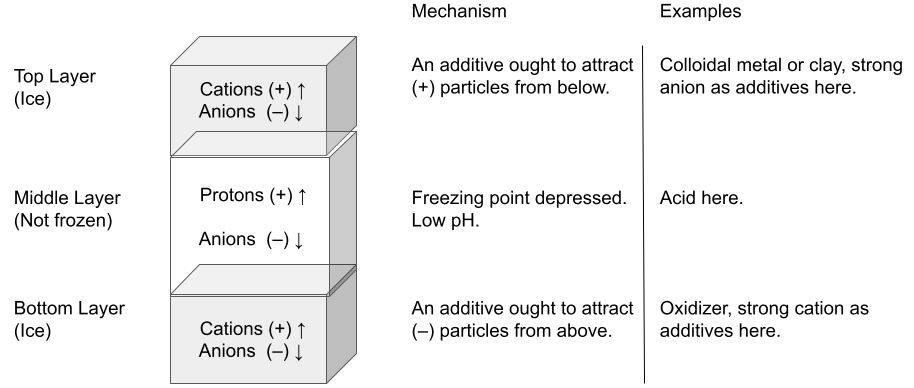


**Figure S1.** Top: General model of a power cell from water ice with different dopants in a top and bottom layer to create separate p- and n- layers as an electronic semiconductor. Bottom: General model for planning an electrochemical cell from water ice. Acid is shown. A base model has charges reversed.

An idealized model for the action of each Experiments 1-4 is listed in Table S1. The theoretical mechanism is based on incident solar radiation liberating charge carriers from the top panel to contribute to energy production. Table S2 shows the same for Experiments 5-6. Here, the theoretical mechanism is based on the motion of protons H+ and hydronium H_2_O^+^ in the middle layer, with additives in solution and suspension in the top and bottom layers, designed to promote coordinated motion of charge carriers, with positive going one direction and negative the other. The top layer should have anions (–) or metals or other sources of electrons in abundance to attract the protons. The bottom layer should have cations (+) or oxidizers in abundance to attract electrons.

**Table S1.** Theoretical Mechanism for the Charge Characteristics of Each Prototype Panel (Semiconductor Model).

| **Expt** | **Panel Composition** | | **Mechanism** | |
| --- | --- | --- | --- | --- |
| 1 | Top:  Bottom: | H_2_O  H_2_O | Top:  Bottom: | mobile H^+^ (p type)  available OH^–^ (n type) |
| 2 | Top:  Bottom: | H_2_O + monopotassium phosphate (KH_2_PO_4_)  H_2_O | Top:  Bottom: | mobile H^+^, K^+^ (p type)  available OH^–^ (n type) |
| 3 | Top:  Bottom: | H_2_O + rochelle salt (KNaC_4_H_4_O_6_·4H_2_O)  H_2_O | Top:  Bottom: | mobile H^+^, K^+^, Na^+^ (p type)  available OH^–^ (n type) |
| 4 | Top:  Bottom: | H_2_O + acetic acid (HC_2_H_3_O_2_) + iron filings (Fe)  H_2_O + ammonia (NH_3_) | Top:  Bottom: | mobile H^+^, Fe^3+^ (p type)  available OH^–^ (n type) |

**Table S2.** Theoretical Mechanism for the Charge Characteristics of Each Prototype Panel (Electrochemical Model).

| **Expt** | **Panel Composition** | | **Mechanism** | |
| --- | --- | --- | --- | --- |
| 5 | Top:  Middle:  Bottom: | H_2_O (2.5 L) + kaolinite (50 g) + sucrose (50 g)  H_2_O + acetic acid (HC_2_H_3_O_2_) (5%)  H_2_O (2.5 L) + limestone (CaCO_3_) (100 g) | Top:  Middle:  Bottom: | Sucrose hydrolysis (↓) kaolinite e^–^ charging (↓)  H^+^(↑) [C_2_H_3_O_2_]^–^(↓)  Ca^+^(↑) |
| 6 | Top:  Middle:  Bottom: | H_2_O (2.5 L) + kaolinite (50 g) + NaCl (50 g)  H_2_O + muriatic acid (HCl) (2%)  H_2_O (2.5 L) + MKP (KH_2_PO_4_) (100 g) | Top:  Middle:  Bottom: | Cl^–^(↓) kaolinite e^–^ charging (↓)  H^+^(↑) Cl^–^(↓)  K^+^(↑) H^+^(↑) |

The original design plans for Experimental Seasons 1 and 2 are given in Tables S3 and S4, and are included for interest. All ionic compounds as additives were chosen for safety and availability. In general, the safest ionic compounds to work with are those that are common in nature geologically: sodium chloride, calcium/magnesium sulfate, and sodium bicarbonate. None of the materials listed in these tables is particularly dangerous nor expensive.

**Table S3.** Original Design Plans, Experimental Season 1.

| **Trial** | **Materials** |
| --- | --- |
| 1 | Rochelle salt vs. distilled water |
| 2 | Sodium chloride vs. distilled water |
| 3 | Sodium chloride plus iron vs. distilled water |
| 4a | Monopotassium phosphate vs. distilled water |
| 4b | Monopotassium phosphate plus iron vs. distilled water |
| 4c | Sucrose vs. distilled water |
| 4d | Sucrose plus iron vs. distilled water |
| 4e | Calcium/magnesium sulfate plus iron vs. distilled water |
| 4f | Sodium bicarbonate vs. distilled water |
| 5 | Very thin (2 to 4 mm) vs. 10 cm (of the best material from the above) |
| 6 | Very thick (25 cm) vs. 10 cm (of the best material from the above) |
| 7 | [<best material from above, top>/distilled water, bottom] vs. [distilled water/distilled water] |
| 8 | [<best material from above, top>/distilled water plus iron, bottom] vs. [distilled water/distilled water] |
| 9 | The better of Trials 7 and 8, with a fine layer of iron filings between top and bottom layers. |
| 10 | To be determined |
| 11 | To be determined |

**Table S4.** Original Design Plans, Experimental Season 2.

| Trial | **Middle** layer | Top **l**ayer (ice) | Bottom **l**ayer (ice) | Principles |
| --- | --- | --- | --- | --- |
| 1 | Muriatic acid (HCl) | • NaCl  • Kaolinite clay (in solution) | • Monopotassium Phosphate (KH2PO4) | • Cl– ions are traveling down.  • Cl– should react with K+ ions in the bottom layer, leaving the phosphate ions free to release another proton to travel up.  • Hydroxide in the kaolinite clay should attract a proton from the slush layer. |
| 2 | Acetic Acid (HC2H3O2) | • Sugar  • Kaolinite clay (in solution) | • Powdered limestone (CaCO3) | • Acetate ions are traveling down.  • Sugar in the top layer should be subject to hydrolysis, thereby increasing the concentration of acetate and inducing a downward flow.  • Some dissolved calcium ions in the bottom layer should attract acetate. |
| 3 | Hydrogen peroxide (dilute) | • NaCl  • iron filings | • Rochelle salt (KNaC4H4O6·4H2O) | • Electrons will flow downwards.  • Iron filings will provide extra electrons to flow down.  • Rochelle salt will supply extra cations to flow up. |
| 4 | Sodium Hydroxide (NaOH) | • Rochelle salt (KNaC4H4O6·4H2O)  • NaCl | • Kaolinite clay (in solution)  • iron filings | • The sodium ions should travel down and the hydroxide or anions should travel up.  • The two salts in the top layer increase the sodium content and promote downward travel of sodium.  • The metal at the bottom promotes upward travel of electrons.  • They hydroxide in kaolinite promotes upward travel of hydroxide. |
| 5 | Ammonia | • MgSO4  • Monopotassium Phosphate (KH2PO4) | • Kaolinite clay (in solution)  • iron filings | • The ammonia layer will scavenge hydrogen.  • OH– and other anions should travel up.  • The two salts in the top layer provide cations to travel down.  • The two additives in the bottom provide electrons or hydroxide ions to travel up. |

Experiments took place in Ottawa, Canada and used ambient cold-weather conditions. Temperature and voltage data were taken at 1-minute intervals. Resistance measurements were taken before/after for Experiments 1-4 and iteratively for Experiments 5-6. These experiments and trials are abbreviated in the text in places by E for experiment and T for trial. E1T1, for example, is Experiment 1 Trial 1. There were two experimental seasons with different panel design rules. Year 1 relied on a p/n junction between two layers of ice modeled as a semiconductor, while Year 2 treated the cells as part of an electrochemical battery with a liquid acidic (or basic) layer between. Year 1 consisted of Experiments 1-4 (E1-4) and Year 2 of Experiments 5-6 (E5-6). Experiment 1 consisted of 3 trials (E1T1-3), Experiments 3, 5 and 6 of 2 trials (E3T1-2; E5T1-2; E6T1-2), and all others were just 1 trial (E2; E4). The main paper describes Experiment 6, which occurred last, during the second experimental season. The other experiments are included here for interest.

A total of six runs were attempted during the first experimental season, and compositional data are shown in Table S5. The top- and bottom-layer thicknesses were reduced from 4 cm to 2 cm (error: ±0.3 cm) after Run 2 to allow for faster freezing times. Run 2 used the same panels as Run 1 as Run 1 data had been lost from Panel A due to a computer fault. Run 3 was aborted due to a thaw in temperatures. Only one panel was run during Run 6 to free up the second DMM to take resistance readings, but the circuit to connect the meter was not designed correctly, and the data were not used.

Table S6 shows compositional and thickness data from the two runs that were attempted during the second experiment season. For each experiment, two panels of identical composition were used, typically with one elevated and exposed to face the Sun, and the other placed low, in shade and covered by plywood. Error in thickness is ±0.3 cm.

**Table S5.** Prototype Solar Panels from Ice Compositions and Thicknesses during Experimental Season 1. Panel Area: 0.13 m^2^.*

| Run | Experimental Panel (A) | | Control Panel (B) | | **Height (cm)** |
| --- | --- | --- | --- | --- | --- |
| 1 | Top:  Bottom: | H_2_O + monopotassium phosphate (500 g)  H_2_O | Top:  Bottom: | H_2_O  H_2_O ***Experiment 1 Trial 1*** | 4  4 |
| 2 | Top:  Bottom: | H_2_O + monopotassium phosphate (500 g)  H_2_O ***Experiment 2*** | Top:  Bottom: | H_2_O  H_2_O ***Experiment 1 Trial 2*** | 4  4 |
| 3 | Top:  Bottom: | H_2_O + rochelle salt (500 g)  H_2_O | Top:  Bottom: | H_2_O  H_2_O | 2  2 |
| 4 | Top:  Bottom: | H_2_O + rochelle salt (500 g)  H_2_O ***Experiment 3 Trial 1*** | Top:  Bottom: | H_2_O  H_2_O ***Experiment 1 Trial 3*** | 2  2 |
| 5 | Top:  Bottom: | H_2_O + rochelle salt (500 g)  H_2_O ***Experiment 3 Trial 2*** | Top:  Bottom: | acetic acid (10%) + iron filings (250 g)  ammonia (~ 2%) ***Experiment 4*** | 2  2 |
| 6 | Top:  Bottom: | acetic acid (5%) + iron filings (250 g)  ammonia (~ 2%) | Top:  Bottom: | (no panel)  (no panel) | 2  2 |

*Runs with equipment failure are listed in grey.

**Table S6.** Prototype Solar Panels from Ice Compositions and Thicknesses during Experimental Season 2. Panel Area: 0.13 m^2^.

| **Run** | **Composition** | | **Experiment** | **Height (cm)** |
| --- | --- | --- | --- | --- |
| 1 | Top:  Middle:  Bottom: | H_2_O (2.5 L) + kaolinite (50 g) + sucrose (50 g)  H_2_O + acetic acid (HC_2_H_3_O_2_) (5%)  H_2_O (2.5 L) + limestone (CaCO_3_) (100 g) | ***Experiment 5 Trials 1-2*** | 2.5  3.2  2.5 |
| 2 | Top:  Middle:  Bottom: | H_2_O (2.5 L) + kaolinite (50 g) + NaCl (50 g)  H_2_O + muriatic acid (HCl) (2%)  H_2_O (2.5 L) + MKP (KH_2_PO_4_) (100 g) | ***Experiment 6 Trials 1-2*** | 2.5  3.2  2.5 |

Restaurant bus pans (43.2 cm x 30.5 cm x 11.5 cm; surface area 0.13 m^2^) from HDPE were used to house the panels. These had holes drilled that could be opened during Year 1 to expel meltwater when temperatures were warm enough to melt some of the ice, thereby preventing short circuit via interlayer conduction. During the set up, before the panels were frozen, the holes were covered with duct tape, as shown in Figure S2. The tape was removed after the panels were frozen. Year 2 used a different model (electrochemical cell) and the same type of bus pans were used, but without holes drilled.


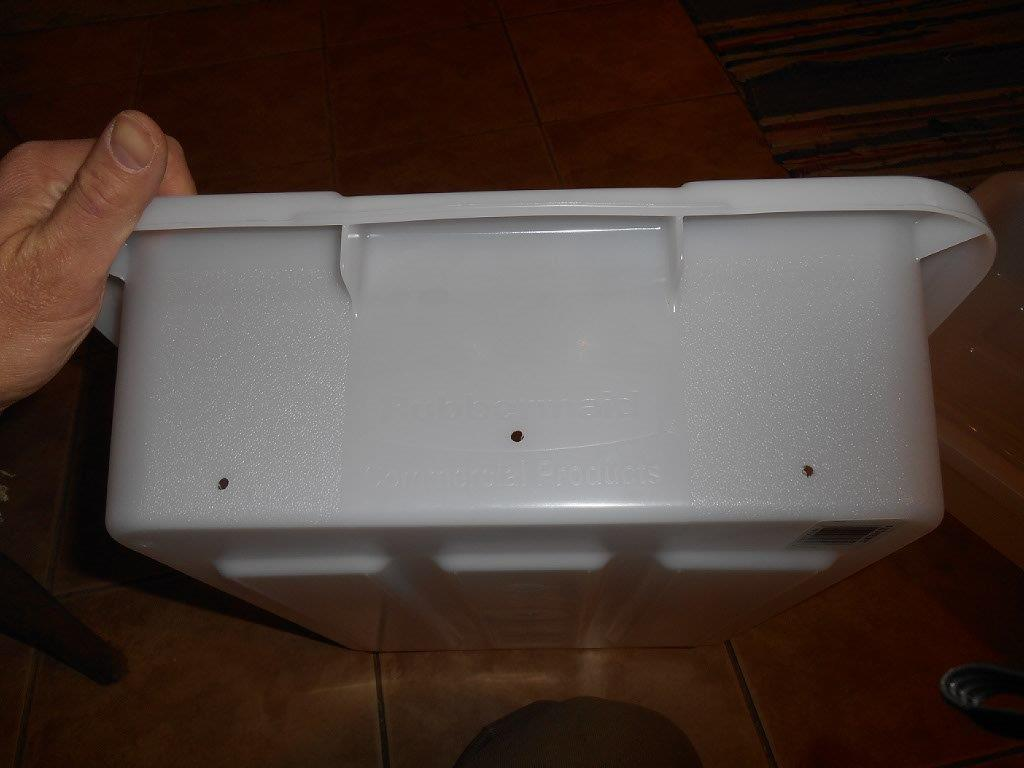
**Figure S2.** Location of drainage holes in the panel tray. Medial and lateral holes are 2.5 cm and 1.3 cm, respectively, above the panel box floor.

Additives were sourced as follows: Monopotassium phosphate (MKP) (KH_2_PO_4_) was purchased from Canadawide Scientific, Ltd. Powdered kaolinite clay, was from an art supplier, and pharmaceutical-grade powdered limestone and rochelle salt (KNaC_4_H_4_O_6_·4H_2_O) were sourced via internet search. NaCl, sucrose acetic acid and muriatic acid were sourced as household items locally. Glass cylinders (tea light holders) to use as spacers in the middle layer during Year 2 trials (E5-6) were sourced from a home goods store. During Year 2 trials (E5-6), the middle layer comprised 4.5 liters of distilled water plus acid. The acetic acid concentrations were as follows: for Experiment 4, 10% acetic acid solution; for Experiment 5, a 5% solution. For Experiment 6, the dilution of muriatic acid was as follows: 286ml of HCl (31.45%) was added to 4.5 liters of distilled water to get a 2% HCl solution.

For Experiments 1-4, panel construction consisted of mixing the distilled water with the solute and/or particles for suspension, and subsequent transfer to the bus pan. The bottom layer was allowed to freeze (via ambient cold) before proceeding with the top layer. For the top layer, mixing and transfer occurred and emplacement over the bottom layer was done by pouring the liquid over the now solid bottom layer. For Experiments 5-6 see the main paper for the fabrication protocol.

For operation, panels were placed on an elevated platform and tilted up approximately 7° towards the west for Experiment 1 (T1-2) and Experiment 2. This was adjusted to 40° for Experiment 1 (T3) and Experiment 3 (T1), as shown in Figure S3. Experiment 3 (T2) and Experiment 4 were done flat, after an attempt to tilt the panels produced leakage of melt in one of the panels, as ammonia and vinegar as solutes depressed the freezing temperature of the ice. For Experiments 5-6, panels were arranged so that one was elevated and exposed, and the other was placed in shade and covered with plywood, and both were laying flat. The arrangement was switched during each trial, so that each panel had equal time in each position.

| 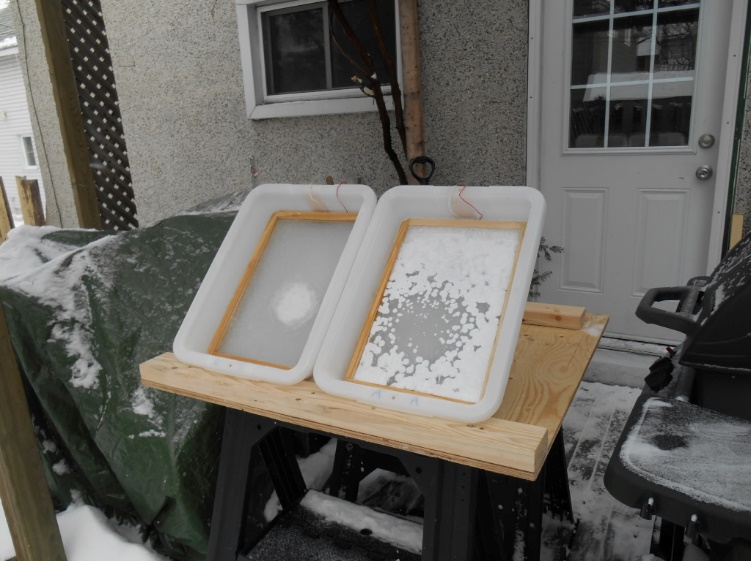 | 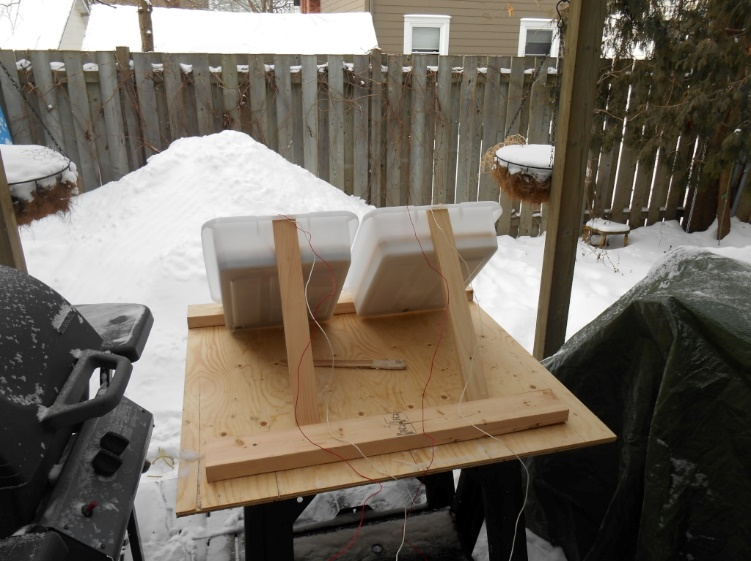 |
| --- | --- |

**Figure S3.** Prototype solar panels adjusted to 40° to face the incoming solar radiation. (a) Front. Left: water-water (Experiment 1, Trial 3). Right: rochelle salt-water (Experiment 3, Trial 1). Note the surface pitting and decreased transparency in the right panel. (b) Back.

Voltage was measured with two handheld UT61 digital multimeters (DMM) manufactured by Uni-T, connected via RS232 to USB converter to a notebook computer. Voltage data were taken at 1-minute intervals. For Experiments 1-4, resistance was measured with one of these DMMs before (E1T1-2; E2) or after (E1T3; E3T1) or both (E3T2; E4) and read by hand (E1; E2; E3T1) or connected to a notebook computer (E3T2; E4) via RS232 to USB. The description in the main article text gives the resistance measurement protocol for Experiments 5-6.

Section S2 Detailed Descriptions of Results

Experiments 1-4 were conducted during the first experimental season and are denoted below as Year 1, and Experiments 5-6 as Year 2. These experiments correspond to the various runs listed in Table S7. The detailed descriptions below follow the notation listed in the table, e.g. Y1R1 Panel B corresponds to E1T1.

**Table S7.** Prototype Solar Panels from Ice Compositions during Experimental Seasons 1 and 2.*

| Yr | Run | Experimental Panel (A) | | Control Panel (B) | |
| --- | --- | --- | --- | --- | --- |
| 1 | 1 | Top:  Bottom: | H_2_O + monopotassium phosphate (500 g)  H_2_O | Top:  Bottom: | H_2_O  H_2_O ***Experiment 1 Trial 1*** |
|  | 2 | Top:  Bottom: | H_2_O + monopotassium phosphate (500 g)  H_2_O ***Experiment 2*** | Top:  Bottom: | H_2_O  H_2_O ***Experiment 1 Trial 2*** |
|  | 3 | Top:  Bottom: | H_2_O + rochelle salt (500 g)  H_2_O | Top:  Bottom: | H_2_O  H_2_O |
|  | 4 | Top:  Bottom: | H_2_O + rochelle salt (500 g)  H_2_O ***Experiment 3 Trial 1*** | Top:  Bottom: | H_2_O  H_2_O ***Experiment 1 Trial 3*** |
|  | 5 | Top:  Bottom: | H_2_O + rochelle salt (500 g)  H_2_O ***Experiment 3 Trial 2*** | Top:  Bottom: | acetic acid (10%) + iron filings (250 g)  ammonia (~ 2%) ***Experiment 4*** |
|  | 6 | Top:  Bottom: | acetic acid (5%) + iron filings (250 g)  ammonia (~ 2%) | Top:  Bottom: | (no panel)  (no panel) |
| 2 | 1 | Top:  Middle:  Bottom: | H_2_O (2.5 L) + kaolinite (50 g) + sucrose (50 g)  H_2_O + acetic acid (HC_2_H_3_O_2_) (5%)  H_2_O (2.5 L) + limestone (CaCO_3_) (100 g)  ***Experiment 5 Trial 1*** | Top:  Middle:  Bottom: | H_2_O (2.5 L) + kaolinite (50 g) + sucrose (50 g)  H_2_O + acetic acid (HC_2_H_3_O_2_) (5%)  H_2_O (2.5 L) + limestone (CaCO_3_) (100 g)  ***Experiment 5 Trial 2*** |
|  | 2 | Top:  Middle:  Bottom: | H_2_O (2.5 L) + kaolinite (50 g) + NaCl (50 g)  H_2_O + muriatic acid (HCl) (2%)  H_2_O (2.5 L) + MKP (KH_2_PO_4_) (100 g)  ***Experiment 6 Trial 1*** | Top:  Middle:  Bottom: | H_2_O (2.5 L) + kaolinite (50 g) + NaCl (50 g)  H_2_O + muriatic acid (HCl) (2%)  H_2_O (2.5 L) + MKP (KH_2_PO_4_) (100 g)  ***Experiment 6 Trial 2*** |

*Runs with equipment failure are listed in grey.

S2.1 Year 1

• Year 1 Run 1: MKP-water and water-water prototypes. 20-23 January 2016.

Results from Year 1 Run 1 (Panel A: MKP-water. Panel B: water-water) are given in Figure S4.

| 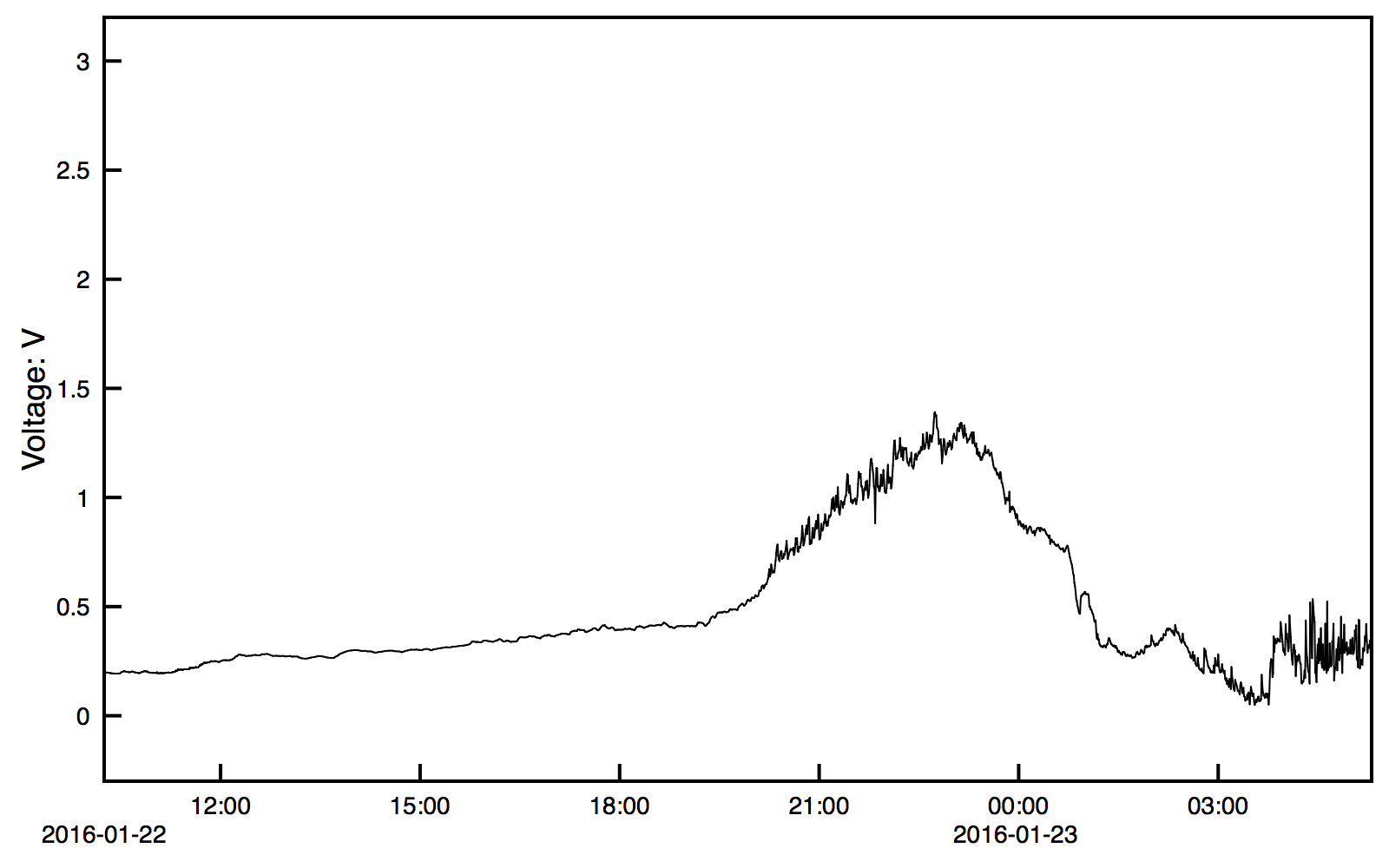 |
| --- |
| 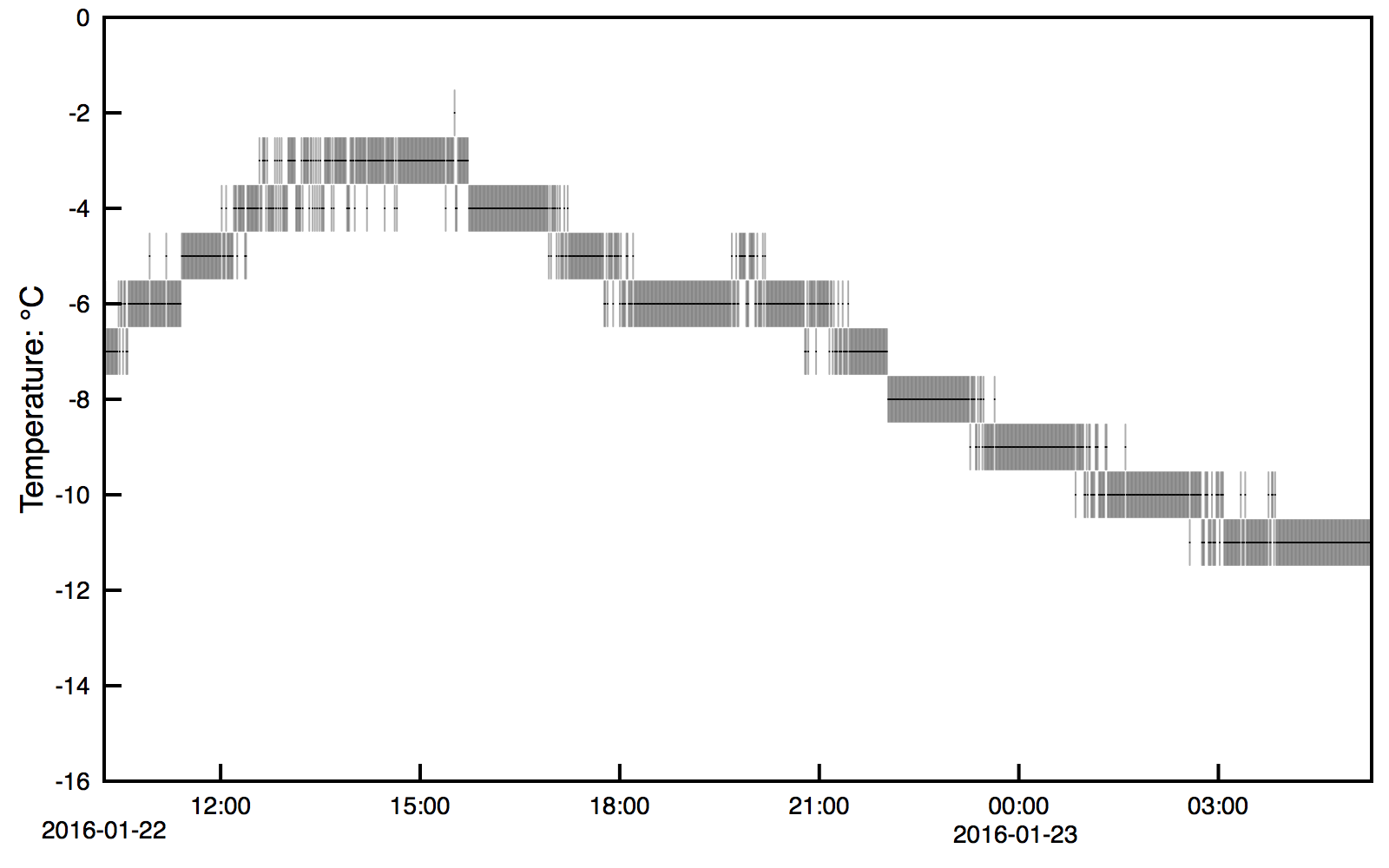 |
| 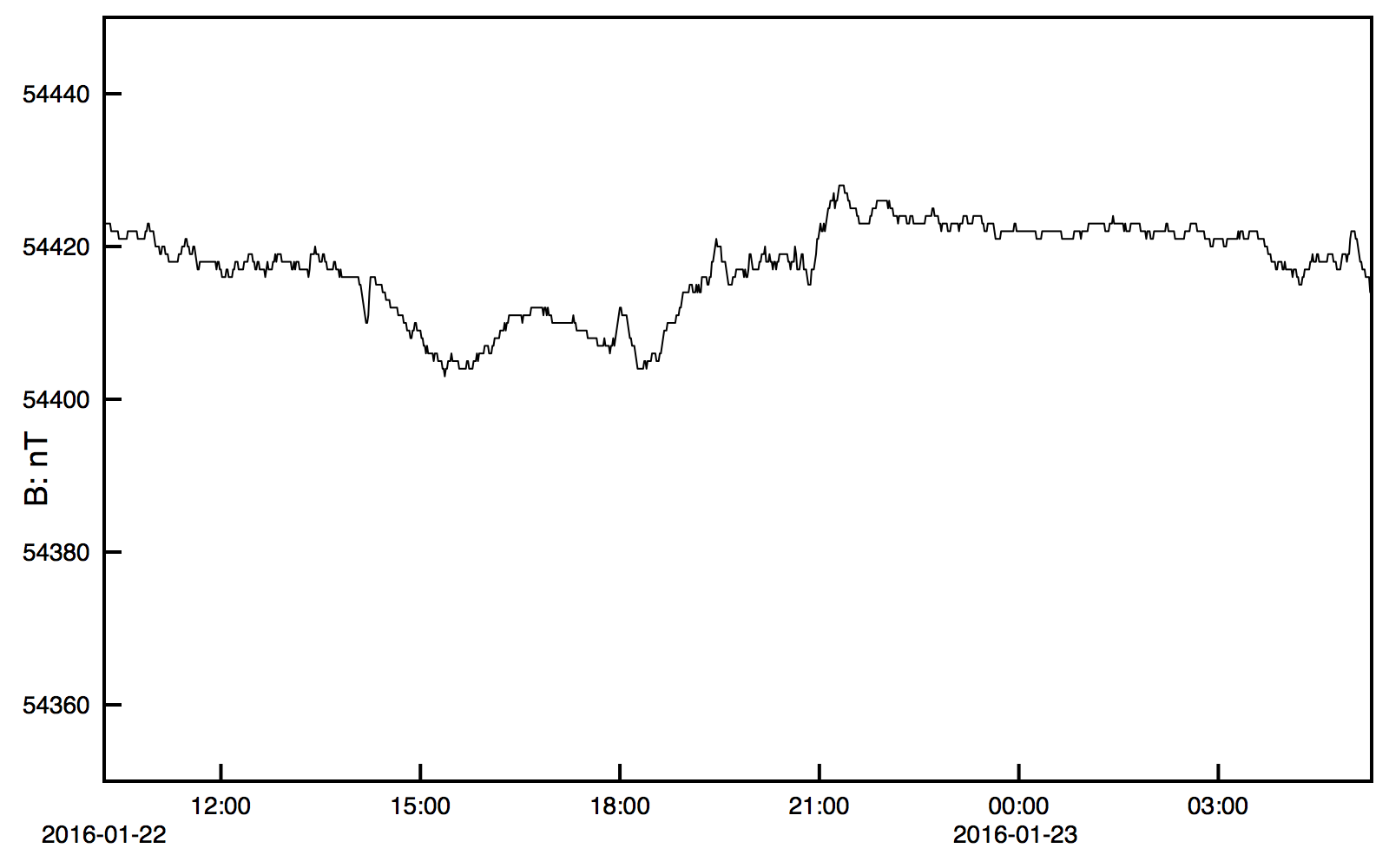 |
| 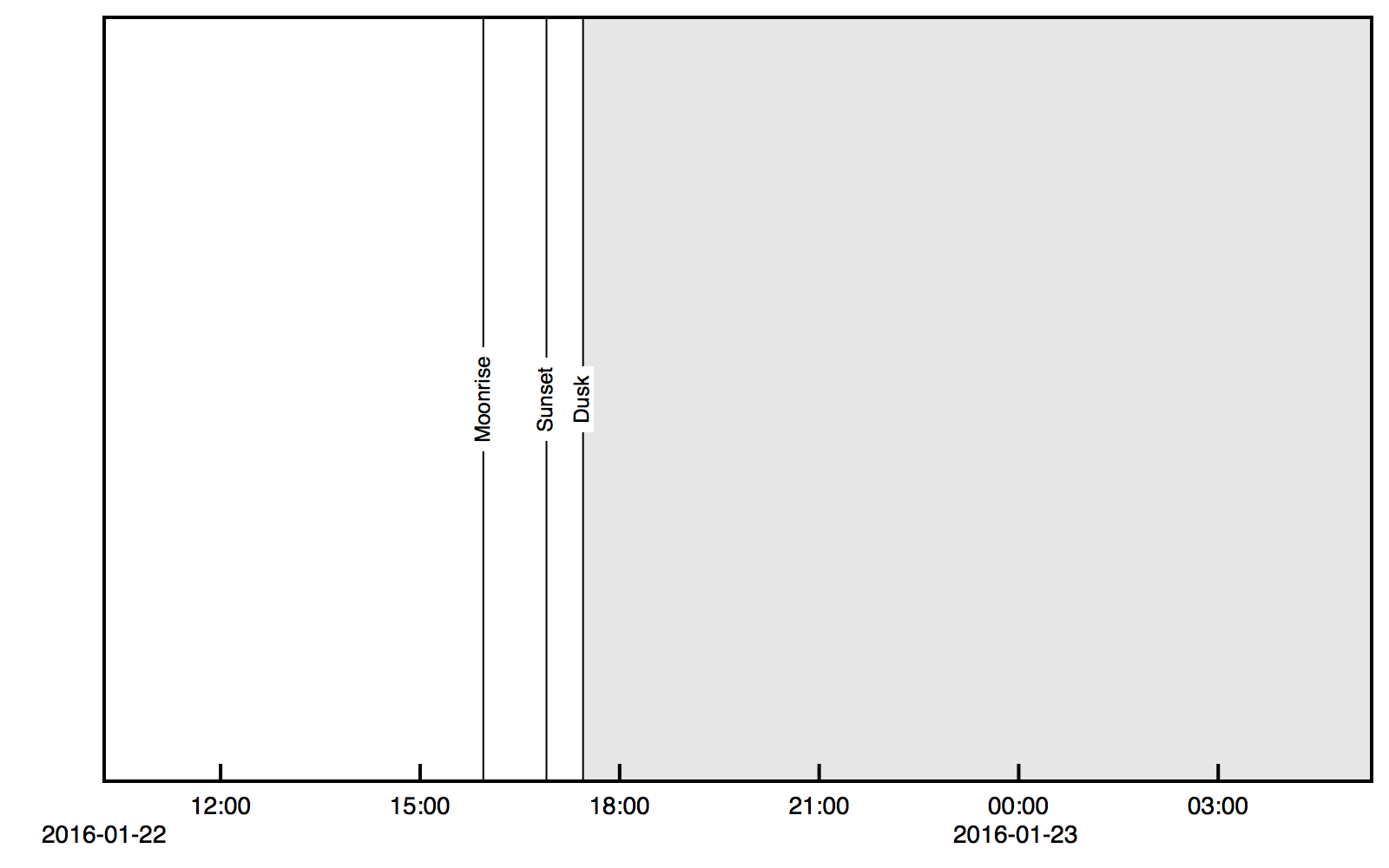 |

**Figure S4.** Data from Year 1 Run 1, 2016-01-22 10:14:58 to 2016-01-23 05:18:25 (Ottawa, ON, Canada, UTC–5). (a) Voltage: Panel B (water-water). (b) Temperature. (c) Total geomagnetic field strength. (d) Lunisolar data. Note: Voltage data from Panel A (MKP-water) were lost via a computer fault.

For Panel B (water-water), measured voltage started at 0.20 V at 10:15 (Jan. 22) and rose (linear) to 0.47 V at 19:28 (Jan. 22), and then rose (convex) to 1.39 V (22:44), with increased variability starting at 20:15. Voltage then fell (concave) to a minimum of 0.030 V (03:42 Jan. 23), but with two local (concave) maxima at 00:44 and 02:16 (Jan. 23). For greater detail: Positive voltage ranged from 0.0302 V (03:42:17 Jan. 23) to 1.3975 V (22:44:18 Jan. 22), with several excursions, none reaching lower than the base, and one reaching as high as 3.049 V (04:44:57 Jan. 23), and with one change in polarity excursion to –0.1815 V (04:44:58 Jan. 23). Noise is prevalent starting 20:14:49 (Jan. 23).

The onset of the period of convex increase in voltage at 19:28 (Jan. 22) is coincident with a brief warm period (from –6°C to –5°C) before temperatures fell steadily again. To describe the overall pattern: Temperatures started at –8°C, rose to –2°C briefly at 15:31 (Jan. 22), and then fell to –11°C steadily but for the brief warm excursion described above. The temperature during the run ranged from –11°C to –2°C.

Total geomagnetic field strength started at ^54^ 423 nT and dropped (sinusoid) to its minimum of ^54^ 403 nT at 15:18 before rising (sinusoid) to a local maximum of ^54^ 412 at 16:40 and then again dropping down (sinusoid) to ^54^ 404 at 18:16. Then field strength rose (convex) to ^54^ 428 at 21:20 before making a slow (concave) descent to ^54^ 421 nT at 03:38 on Jan. 23, and then a more variable descent to ^54^ 414 nT at 05:18. The magnitude of the total geomagnetic field ranged from ^54^ 403 to ^54^ 428 nT.

Moonrise occurred at 15:57 on Jan. 22, and sunset was at 16:54, with dusk occurring 33 minutes later at 17:27.

Power Output, Year 1 Run 1

Initial resistance measurements of 160 Ω (Panel A, MKP-water), and 205 Ω (Panel B, water-water) were taken after 5 minutes of operation immediately prior to the voltage data recorded for this run. With measured voltage = 0.2 V, the initial calculated current for Panel B (I = V/R) is about 1 mA and calculated power (P = VI) is about 0.2 mW for each panel.

Additional Observations, Year 1 Run 1

The sky was overcast at 14:10 on Jan. 22, then clear at 15:10. Dusk was settling in at 17:00 and the sky was dark at 18:00. Clear skies at 08:30 on Jan. 23.

Distilled water froze at the edges of the panels first. In the center, there was a distinct pattern of aligned nucleation sites of bubbles surrounded by crystalline matrix. Similar patterns were seen in the bottom (distilled water) layers of both Panels A and B. See Figure S5.

The addition of the top layer of liquid water in Panel B (water-water) was accompanied by sounds of cracking (fracture) in the bottom layer. These were absent during the same procedure with Panel A (MKP-water).

In Panel B (water-water), bubbles visible toward the centre of the panel would move as the panel was tilted, indicating there was still liquid water in the middle, between the two layers. There was no observed drainage out of the drain holes.

Overnight both panels developed a bulge of 1 to 2 cm thick, not at the midline, but offset slightly towards the lower end of each panel. Surface pitting of Panel A (MKP-water) was also visible and ongoing, as was progressive loss of transparency.

| 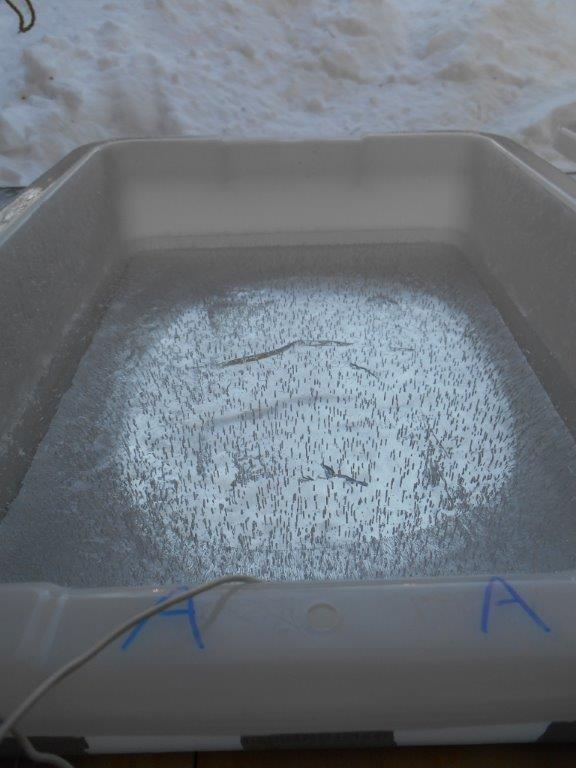 | 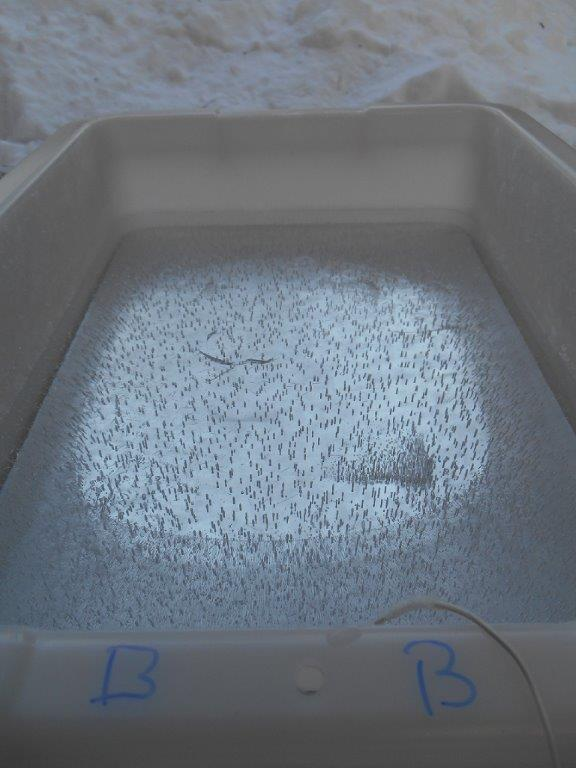 |
| --- | --- |

**Figure S5.** Bottom layer of distilled water in (a) Panel A and (b) Panel B. Note the distinct pattern of bubble nucleation sites as well as the loss of transparency.

• Year 1 Run 2: MKP-water and water-water prototypes, 23-24 January 2016.

Results from Year 1 Run 2 (Panel A: MKP-water. Panel B: water-water) are given in Figure S6.

| 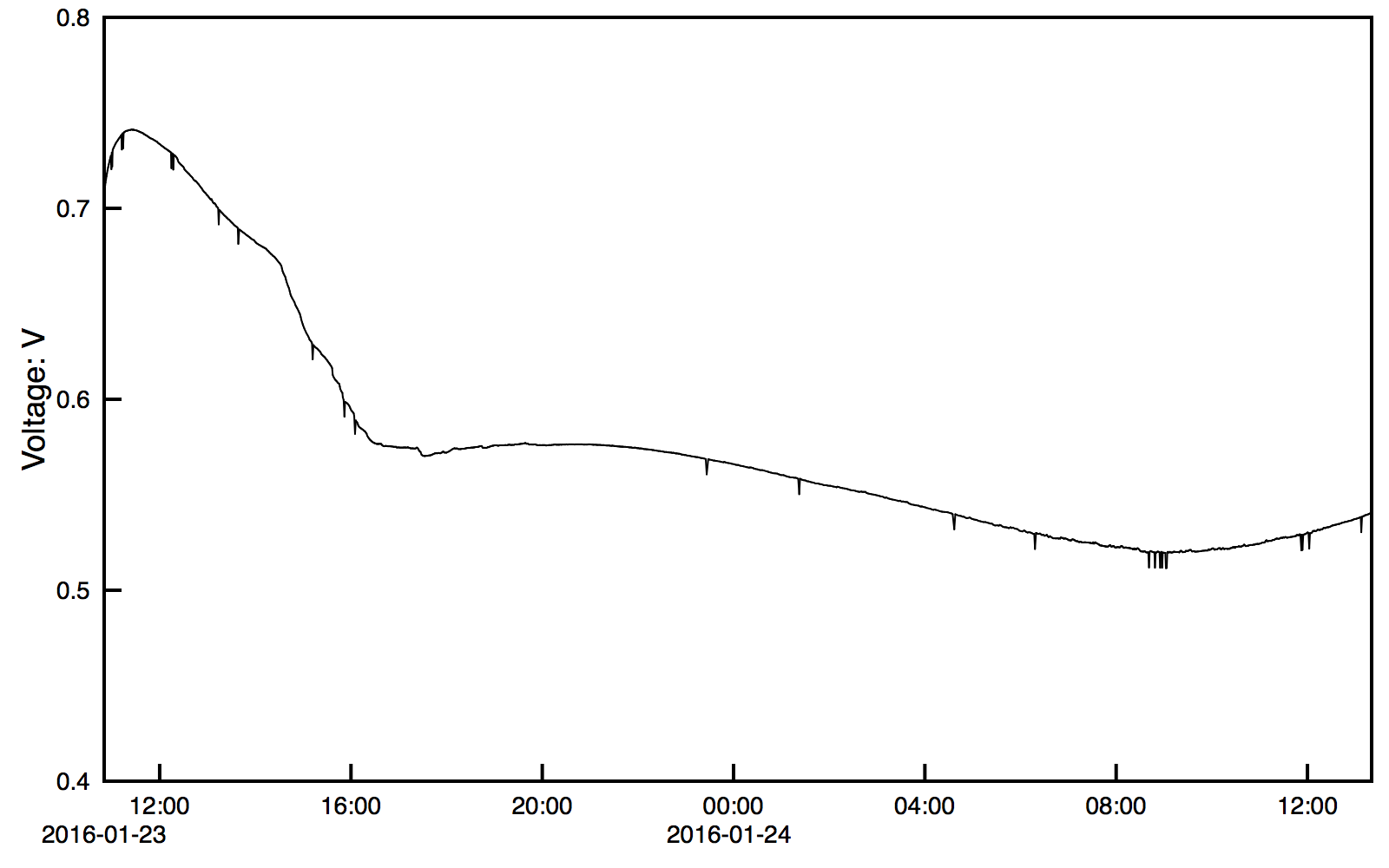 |
| --- |
| 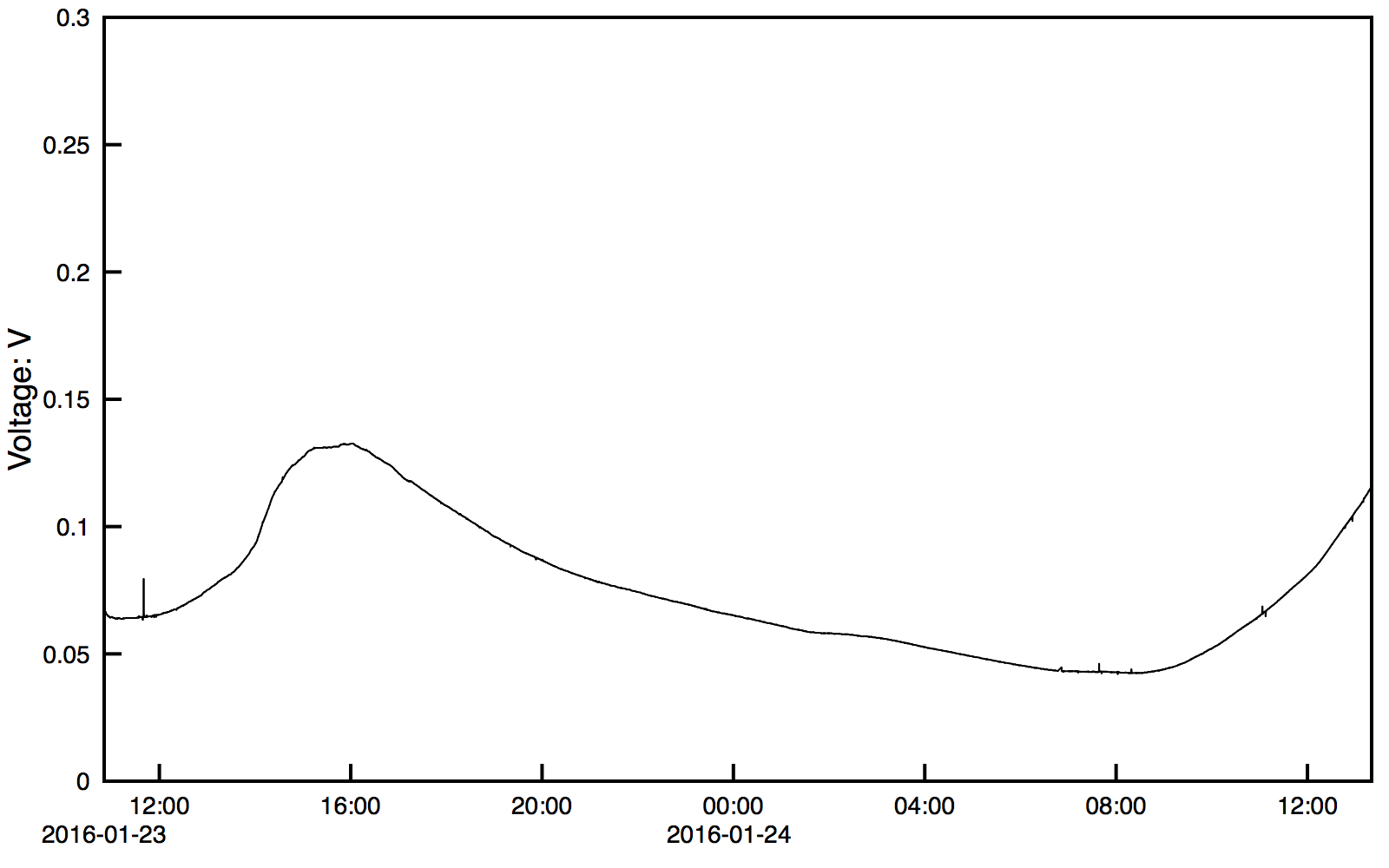 |
| 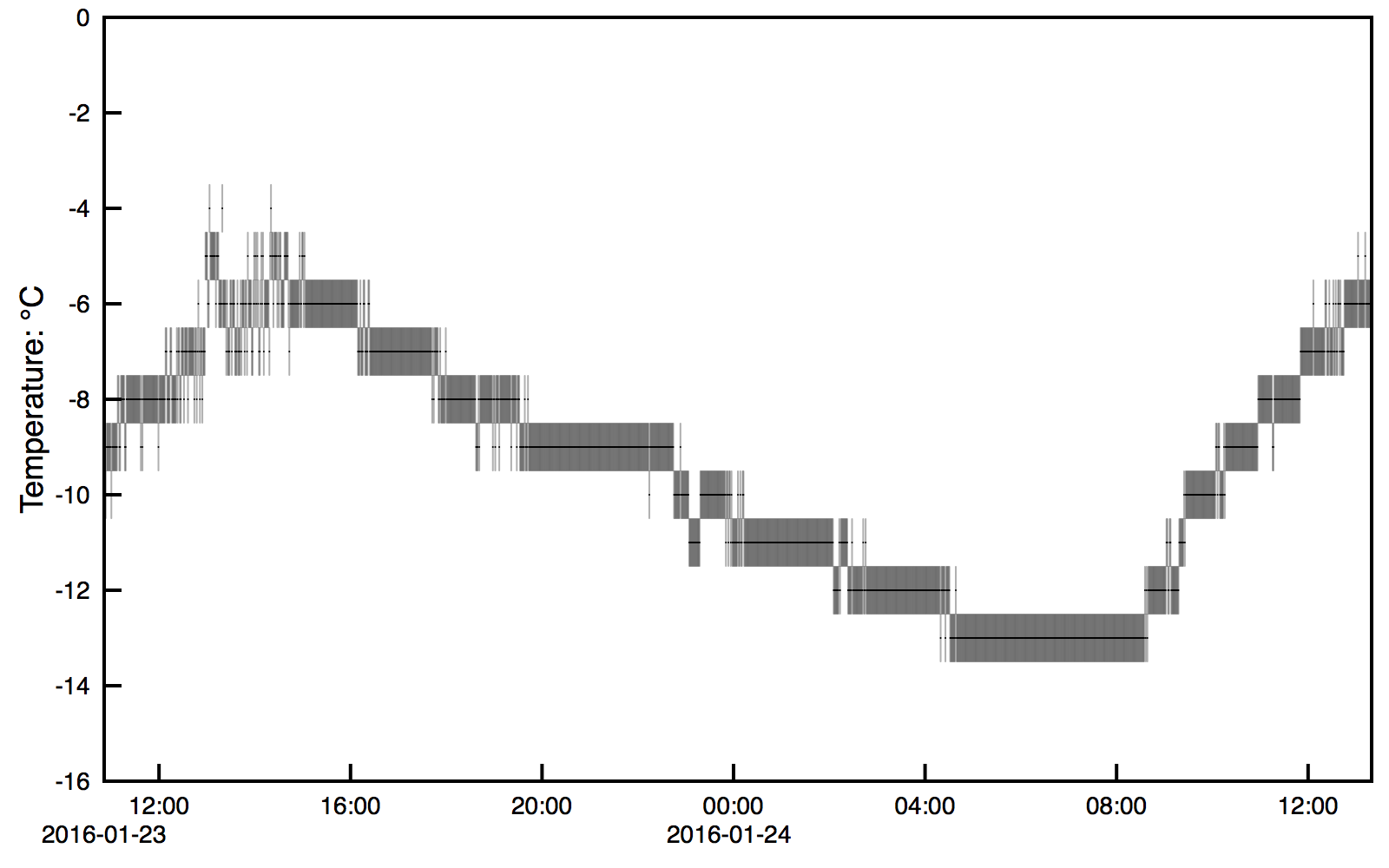 |
| 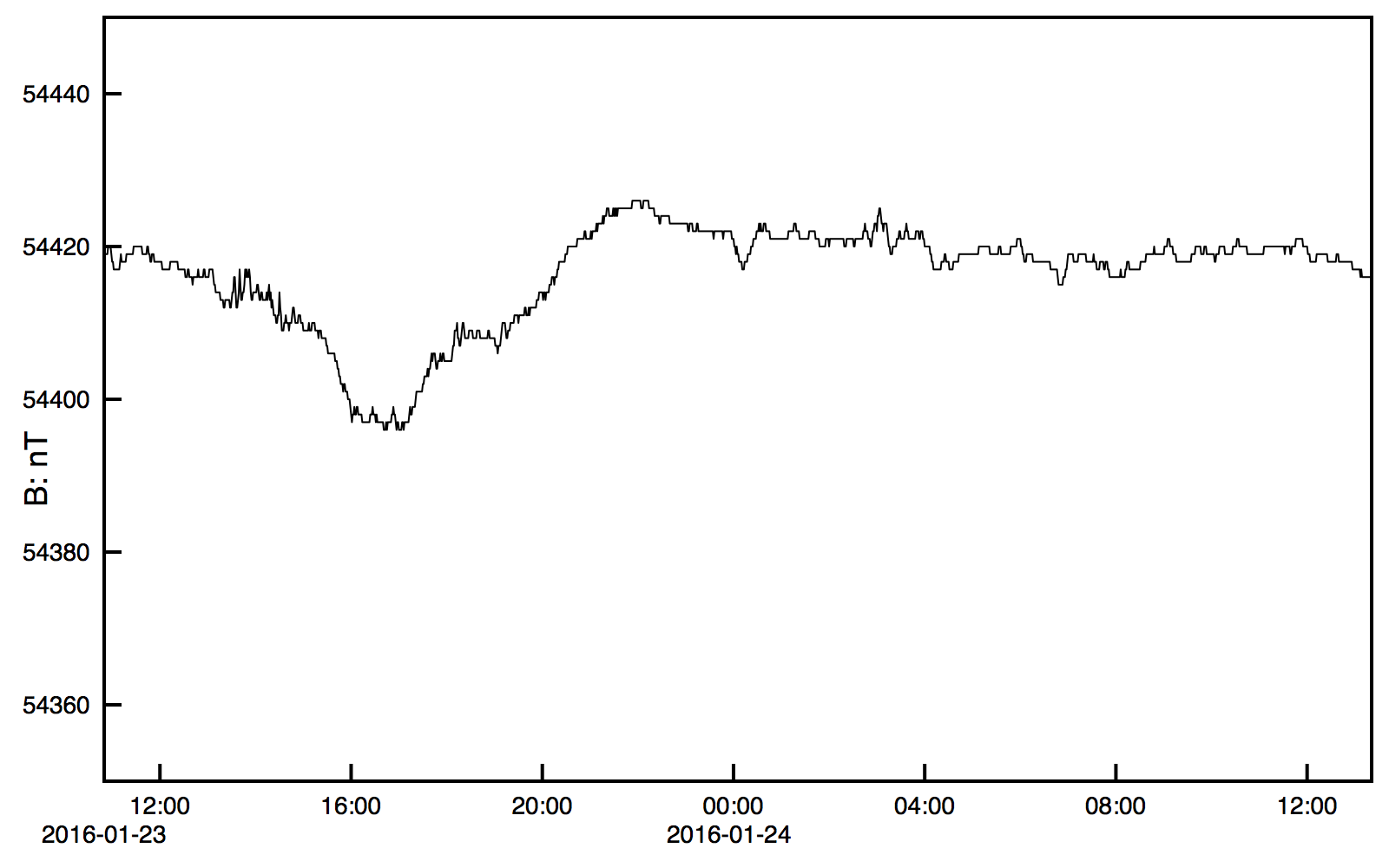 |
| 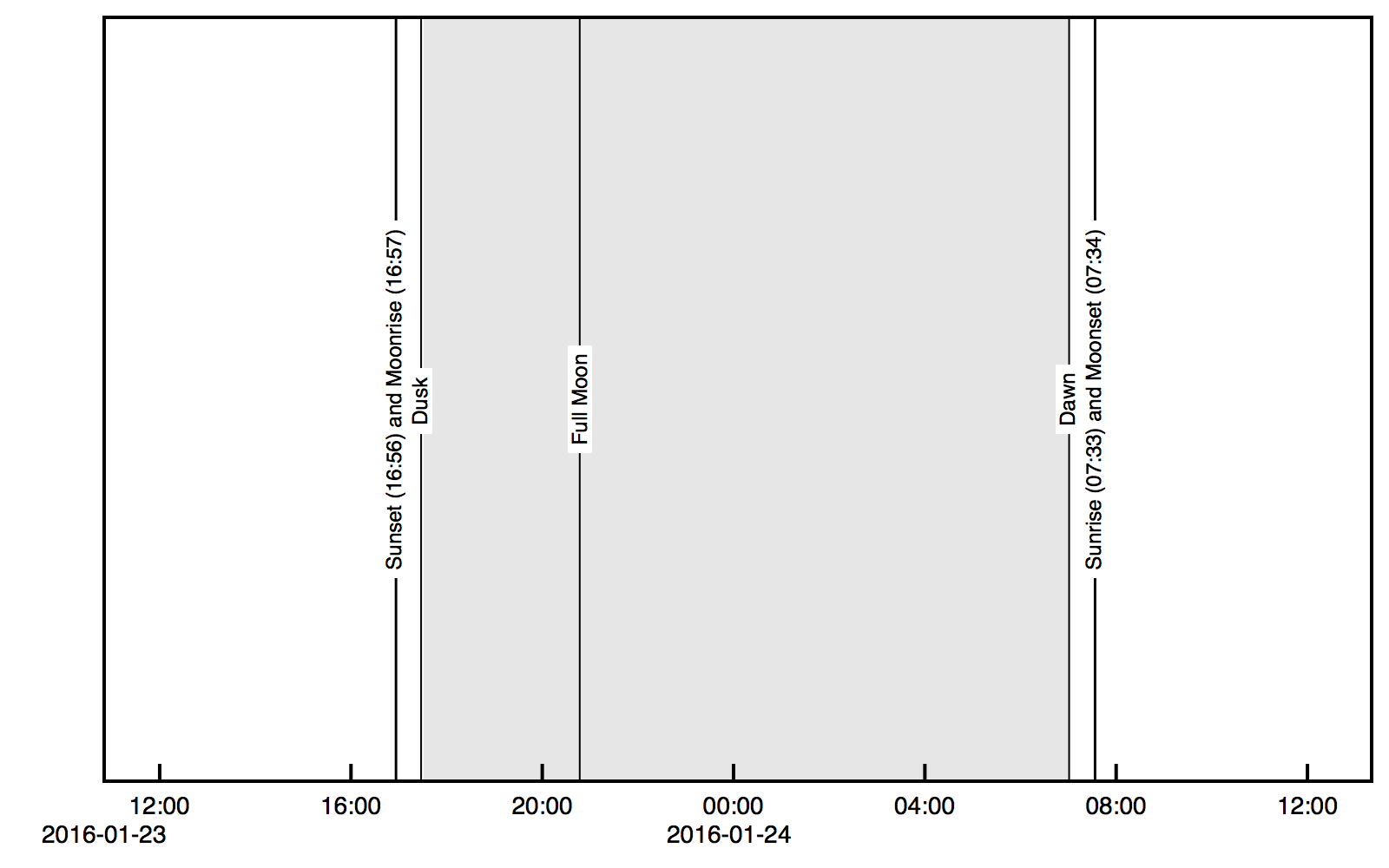 |

**Figure S6.** Data from Year 1 Run 2, 2016-01-23 10:50:22 to 2016-01-24 13:20:31 (Ottawa, ON, Canada, UTC–5). (a) Voltage: Panel A (MKP-water). (b) Voltage: Panel B (water-water). (c) Temperature. (d) Total geomagnetic field strength. (e) Lunisolar data.

For Panel A (MKP-water), initially, voltage started at 0.71 V at 10:50 (Jan. 23) and rose (convex) to 0.74 V at 11:24 (Jan. 23), and then decreased steeply (concave, with two local maxima) to 0.58 V at 16:33, then declined slowly (concave) to 0.57 V until 17:31, and then reached (sinusoid) a slight local peek to 0.58 V at 19:39 before dropping smoothly (sinusoid) to 0.52 V at 09:01 (Jan. 24) and then rose smoothly (sinusoid) again to 0.54 V at the conclusion of the trial at 13:21 (Jan. 24). For greater detail: Voltage magnitude ranged from 0.5196 V (09:01:20 Jan. 24) to 0.7413 V (11:24:02 Jan. 23) with several excursions reaching as low as 0.5116 V (09:00:58 Jan. 24) and with no change in polarity. Excursions were uniformly characterized by a voltage drop of 0.008 V, and no positive excursions were recorded.

For Panel B (water-water), initially, voltage dropped from 0.068 V at 10:50 on Jan. 23 to 0.064 V at 11:04 before rising sinusoidally to a peak of 0.13 V at 15:59. The inflection point was at 14:00 on Jan. 23. From its peak, voltage dropped (concave) to a minimum of 0.042 V at 08:19 on Jan. 24, and then rose (concave) to 0.12 V at the end of the trial at 13:21. For greater detail: Positive voltage ranged from 0.0424 V (08:19:10 Jan. 24) to 0.1326 V (15:58:59 Jan. 23) with several excursions reaching as low as 0.0312 V (11:43:59 Jan. 23), and none reaching higher than peak, and with one change in polarity event to –0.0107 V at 11:30:43 on Jan. 23.

Temperatures started at –10°C (10:51 Jan. 23) and rose steadily to –5°C (12:58) with three excursions up to –4°C (13:03, 13:19, 14:20) and several down as low as –7°C. From 14:56 temperatures fell from –5°C steadily to –13°C at 04:20 (Jan. 24) and remained. Starting at 08:37 temperatures rose steadily (convex) to –6°C at the run’s end at 13:20 with a few excursions to –5°C. Temperatures ranged from –13°C to –4°C. The rise and fall of temperature approximate the rise and fall of voltage in Panel B (water-water).

Total geomagnetic field strength started at ^54^ 419 nT at 10:51 (Jan. 23) and dropped (sinusoid) to its minimum of ^54^ 396 nT at 16:41 before rising (sinusoid) to a maximum of ^54^ 426 at 21:53 and then gradually dropping down (sinusoid) to ^54^ 415 at the end of Run 2 at 13:21 (Jan. 24). The magnitude of the total geomagnetic field ranged from ^54^ 396 to ^54^ 426 nT.

Sunset occurred at 16:56 (Jan. 23) and moonrise happened one minute later at 16:57, with dusk coming at 17:28. The full moon here was on this day at 20:47. Dawn occurred at 07:01 (Jan. 24) with sunrise 32 minutes later at 07:33, and moonset one minute later at 07:34.

Power Output, Year 1 Run 2

Initial resistance measurements were taken before the voltage data collection. The DMM did not give a stable resistance reading, but after 90 minutes, measurements tended towards 18 kΩ (Panel A, MKP-water), and 3.5 MΩ (Panel B, water-water). For Panel A, with measured voltage = 0.7 V, the initial calculated current for Panel A (I = V/R) is about 40 µA and calculated power (P = VI) is about 30 µW. For Panel B, with measured voltage = 0.07 V, the initial calculated current for Panel B (I = V/R) is about 20 nA and calculated power (P = VI) is about 1 nW.

Additional Observations, Year 1 Run 2

Dawn was breaking at 06:30 on Jan. 23, and skies were sunny, cool and clear at 08:30. At 14:00 there were clear skies and direct sunlight on the panels.

Pitting and loss of transparency in the surface of Panel A and surface bulging of Panel B continued throughout this run. See Figure S7.

| 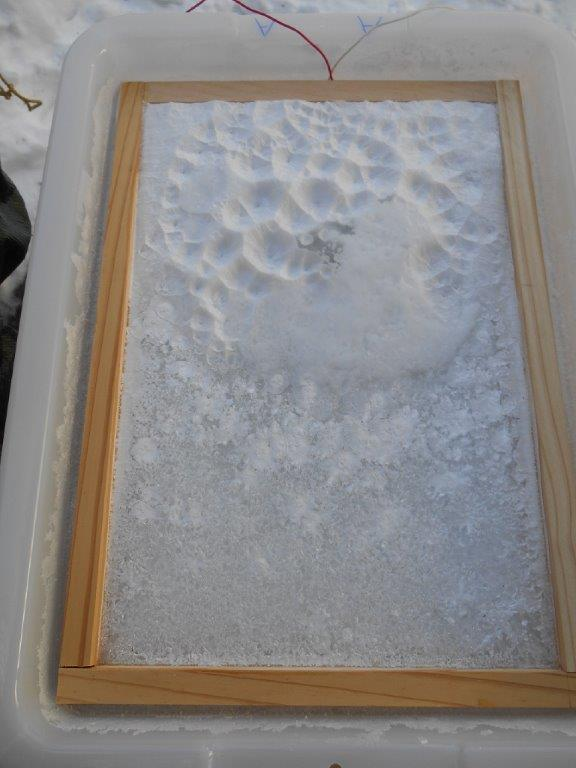 | 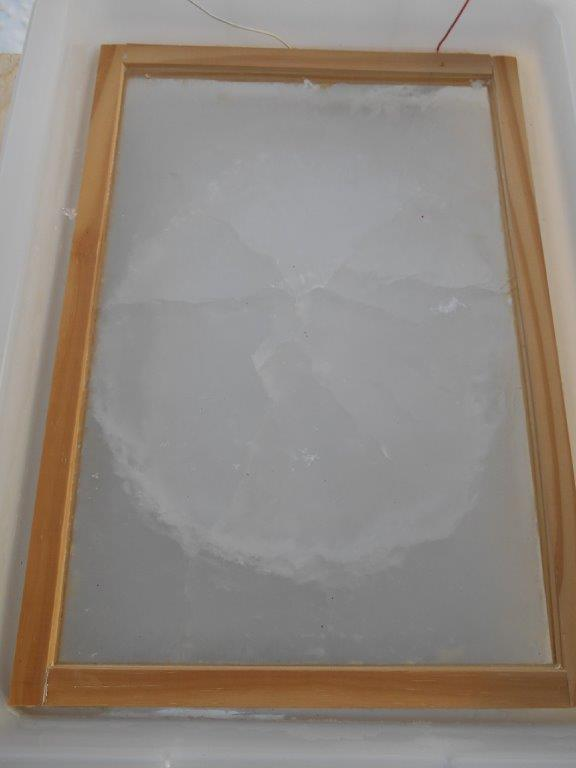 |
| --- | --- |

**Figure S7.** (a) Surface pitting of Panel A (MKP-water) after operating overnight. (b) Bulge in Panel B (water-water) after operating overnight. Note also the loss of transparency. These pictures are of the same panels used in Year 1 Run 1 and the loss of transparency is notable when compared to Figure S5.

• Year 1 Run 3: rochelle salt-water and water-water prototypes, 4-6 February 2016.

Results from Year 1 Run 3 (Panel A: rochelle salt-water. Panel B: water-water).

The panels were prepared on Feb. 4 and Feb. 5, for a start on Feb. 6. The temperature overnight (Feb. 5) was frequently above the freezing point of the rochelle salt-water panel (about –1.4°C for 10 g/100 ml). On Feb. 6, the weather warmed above 0°C for an extended period of time, and the run was abandoned. No data were gathered.

• Year 1 Run 4: rochelle salt-water and water-water prototypes, 11-15 February 2016.

Results from Year 1 Run 4 (Panel A: rochelle salt-water. Panel B: water-water) are given in Figure S8.

| 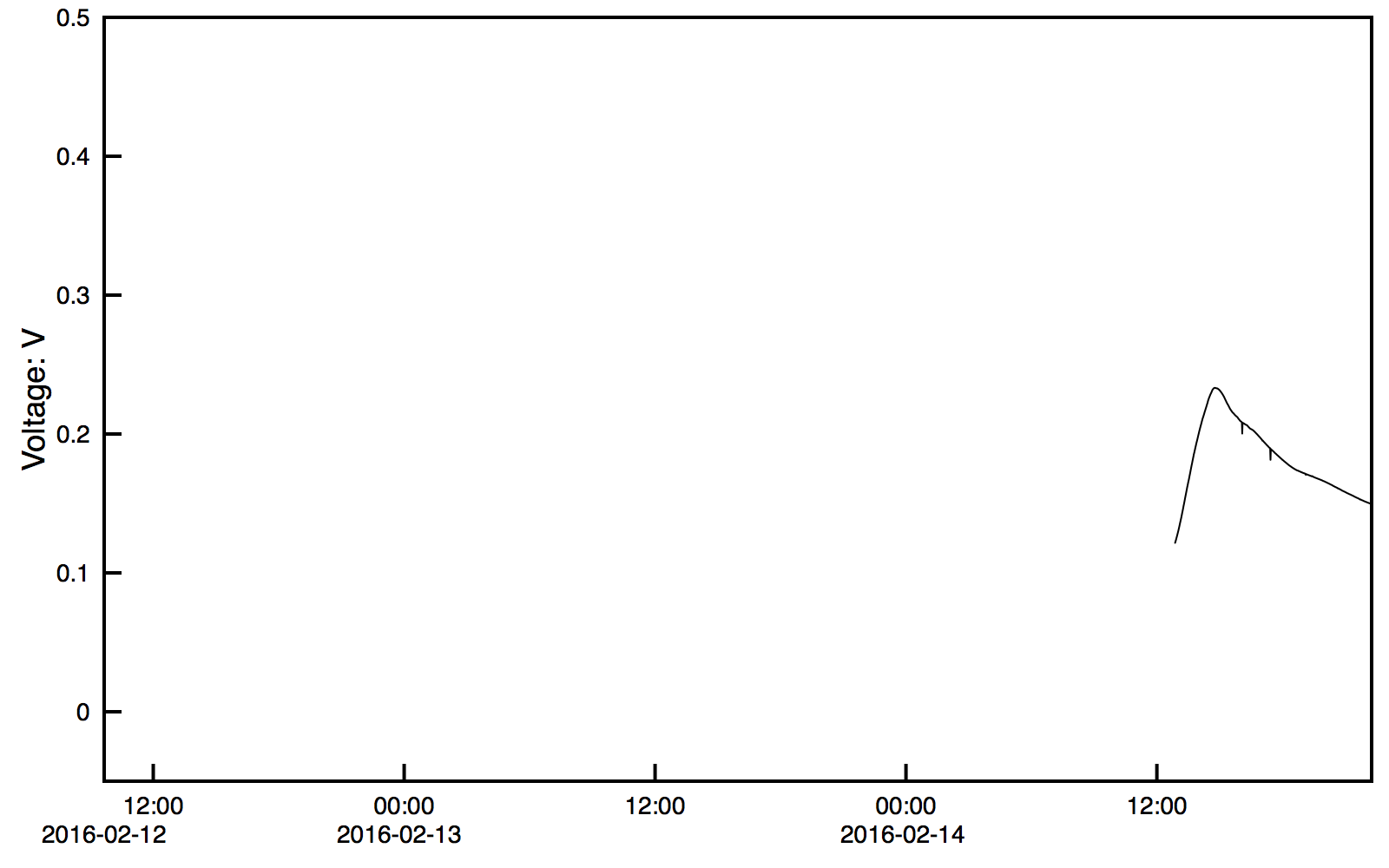 |
| --- |
| 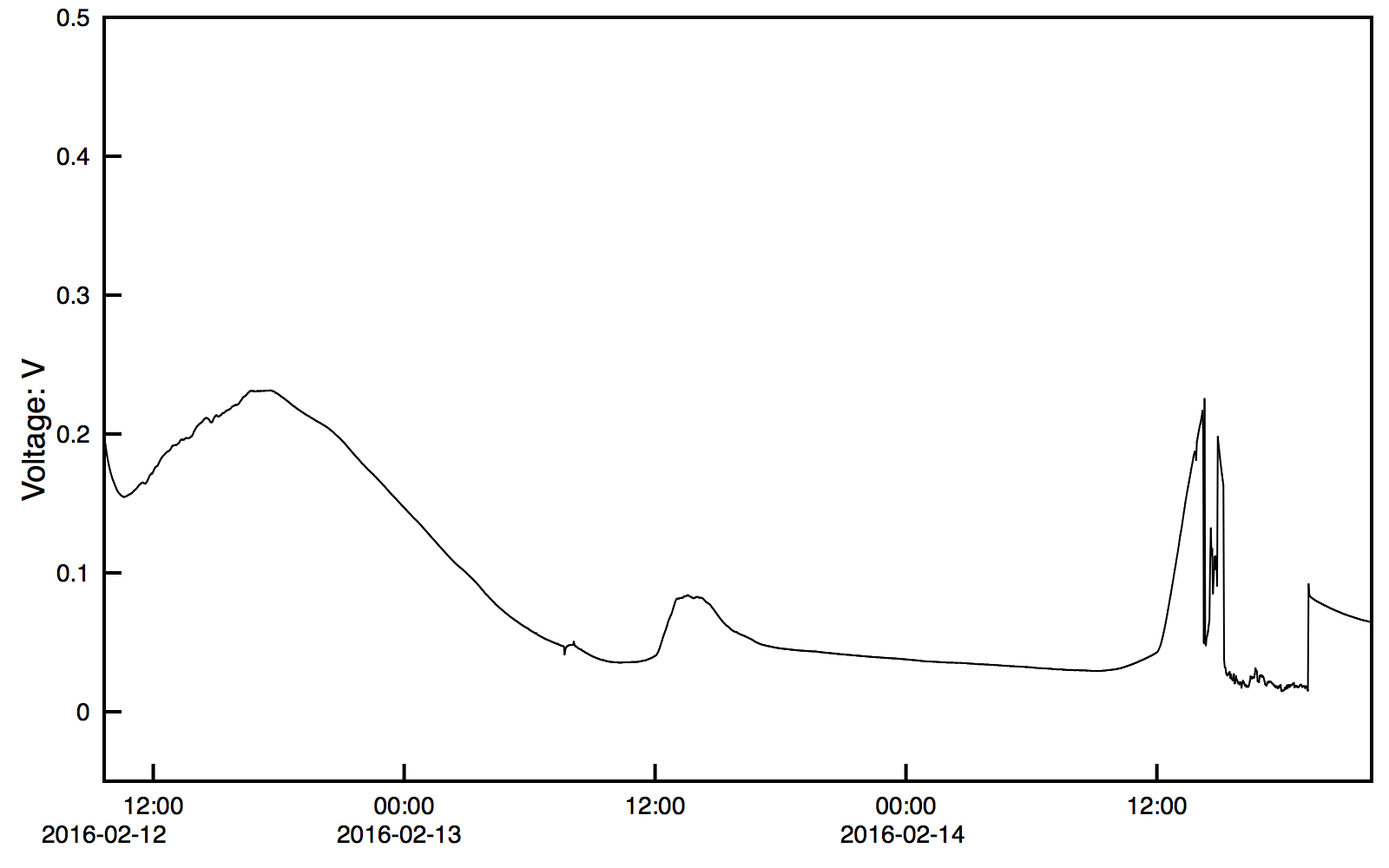 |
| 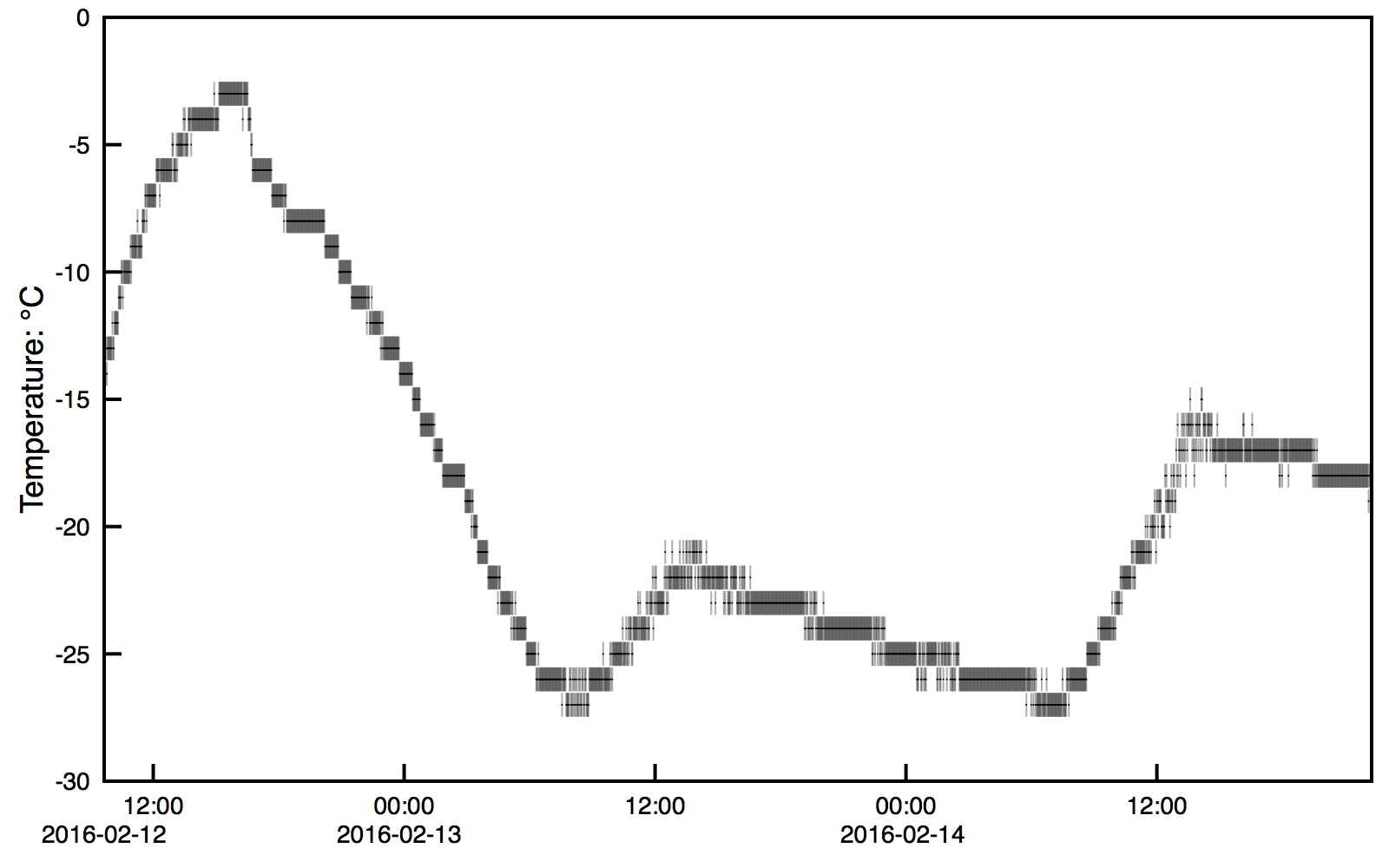 |
| 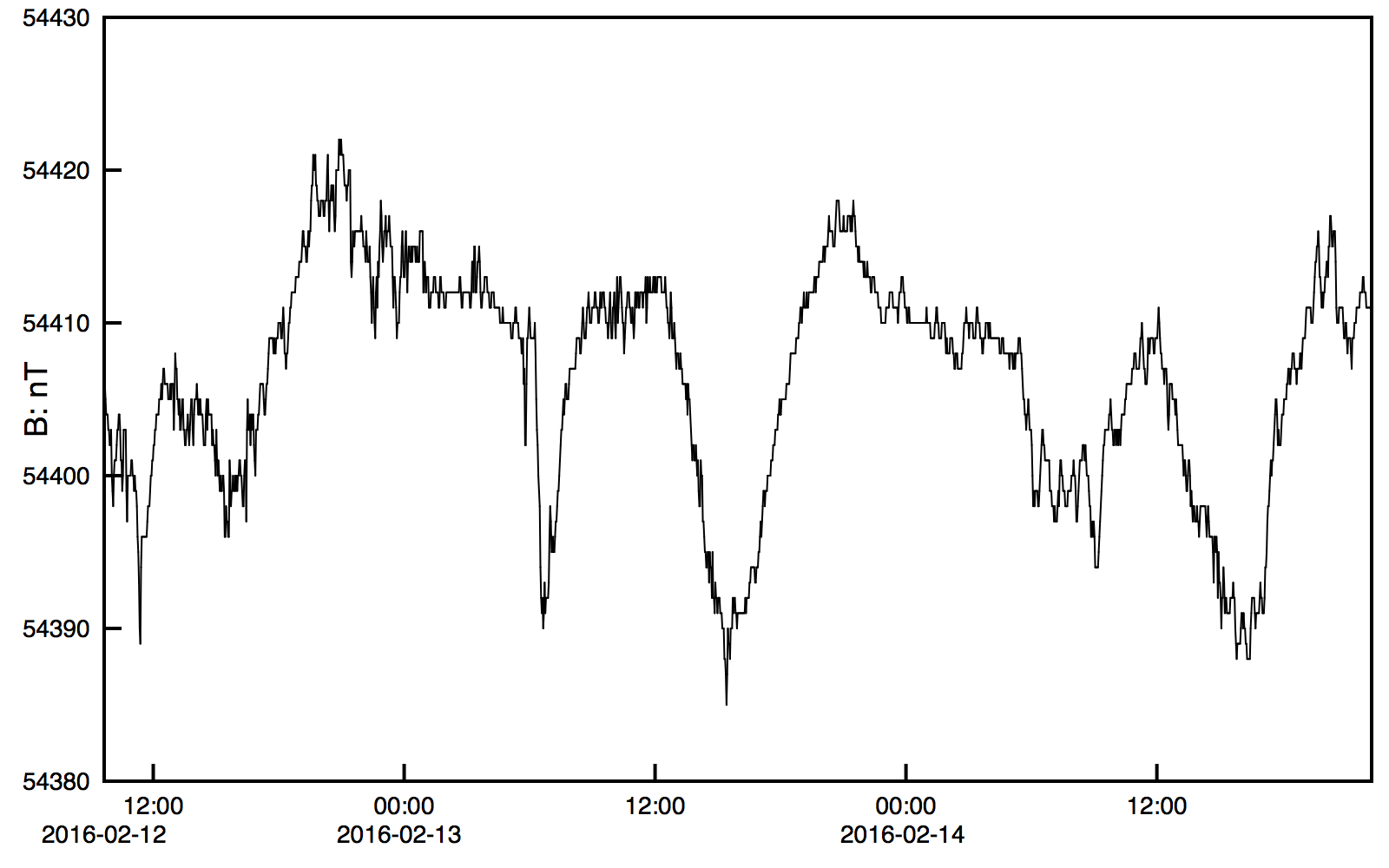 |
| 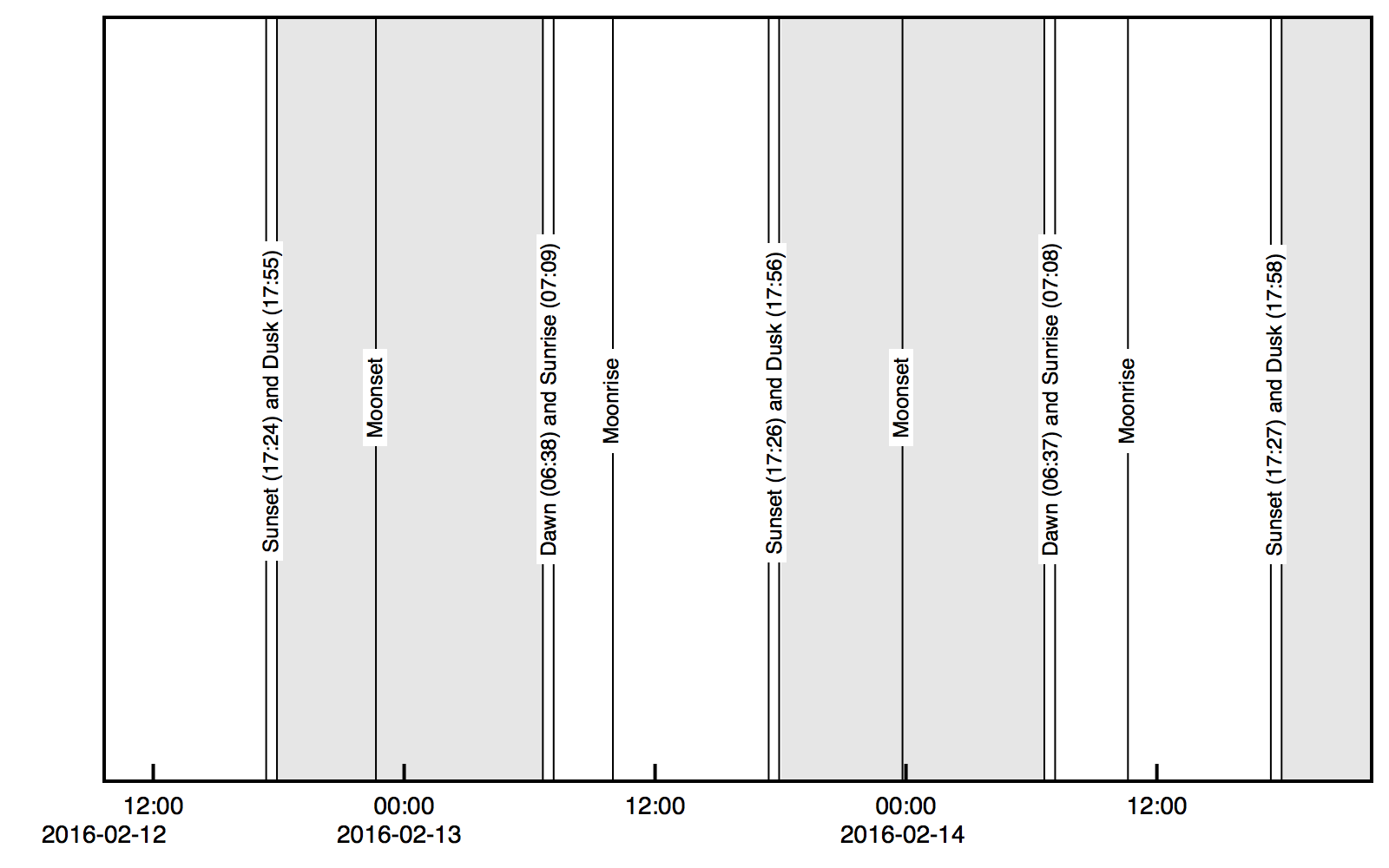 |

**Figure S8.** Data from Year 1 Run 4, 2016-02-12 09:39:08 to 2016-02-14 22:16:15 (Ottawa, ON, Canada, UTC–5). (a) Voltage: Panel A (rochelle salt-water). Note that all data were lost prior to 12:52 on Feb. 14 due to a computer fault. (b) Voltage: Panel B (water-water). (c) Temperature. (d) Total geomagnetic field strength. (e) Lunisolar data.

For Panel A (rochelle salt-water), all voltage data were lost prior to 12:52:13 on Feb. 14 due to a computer fault. After that, voltage started at 0.12 V at 12:52 (Feb. 14) and rose (sinusoid) to 0.23 V at 14:45, and then decreased (concave) to 0.15 V at the conclusion of the trial at 22:16. For more detail: Voltage magnitude ranged from 0.1216 V (12:52:13) to 0.2332 V (14:44:47) with two negative excursions of 0.008 V each (16:04:54, 17:26:01). There was no change in polarity. Note: Seven corrections were made to the data where the DMM had switched units during logging, i.e. isolated peak values were divided by 100 where nearest neighbors matched the new value and there were only two digits recorded instead of four.

For Panel B (water-water), voltage started at 0.20 V at 09:39 (Feb. 12) and fell (concave) to 0.15 V at 10:35, and then rose (sinusoid) to 0.23 V at 16:40 where it stayed until 17:37. Voltage then fell (concave) to 0.04 V at 10:14 (Feb. 13) before rising (concave) to 0.08 V at 13:03 where it stayed until 14:14, and then fell (concave) to 0.03 V at 09:06 (Feb. 14) and then rose (concave) to 0.23 V at 14:17. There was a period of peaks with troughs until 14:54, with peaks of similar magnitudes and troughs as low as 0.05 V. The voltage then dropped (concave) from 0.20 V (14:54) to 0.16 V (15:12). From then until 19:14, voltage varied between 0.03 V and 0.01 V and was noisy. At 19:15, voltage rose (linear) to 0.09 V and then fell smoothly (concave) to 0.06 V at the end of the trial (22:15). For more detail: Voltage magnitude ranged from 0.0137 V (17:58:07 Feb. 14) to 0.2315 V (17:37:12 Feb. 12) with few excursions until 08:10:47 (Feb. 14), none reaching lower than the base nor higher than the peak. The data after that time are noisy. There was no change in polarity.

Temperatures started at –15°C (09:39 Feb. 12) and rose (convex) to –3°C (15:08), and then fell (sinusoid, two cycles) overnight to –27°C at 07:04 (Feb. 13). Temperatures rose to –21°C at 13:11, and then fell again overnight to –27°C at 05:56 (Feb. 14). Temperatures then rose to –16°C at 12:59, with seven brief excursions up to –15°C, and then fell to –18°C at the end of the run at 22:16, with a few brief excursions down to –19°C. Temperatures ranged from –27°C to –3°C. As with Run 2, the rise and fall of temperature approximate the rise and fall of voltage in Panel B (water-water).

Total geomagnetic field strength started at ^54^ 408 nT at 09:39 (Feb. 12) and ended at ^54^ 411 at 22:16 (Feb. 14) and was noisy throughout. There were two periods of peaks and decay: ^54^ 422 to ^54^ 411 from 19:39 (Feb. 12) to 06:14 (Feb. 13); and ^54^ 419 to ^54^ 410 from 20:42 (Feb. 13) to 05:24 (Feb. 14). There were six troughs: 11:15 to 11:27 (Feb. 12) to a local minimum of ^54^ 388 nT; 14:56 to 16:52 to a local minimum of ^54^ 395 nT; 06:22 to 07:30 (Feb. 13) to a local minimum of ^54^ 390 nT; 14:22 to 16:59 to a run minimum of ^54^ 385 nT; 05:40 to 10:28 (Feb. 14) to a local minimum of ^54^ 394 nT; and 13:37 to 17:22 to a local minimum of ^54^ 388 nT. The magnitude of the total geomagnetic field ranged from ^54^ 385 to ^54^ 422 nT.

On Feb. 12, moonrise occurred at 09:24, sunset at 17:24, dusk at 17:55 and moonset at 22:39. On Feb. 13, dawn occurred at 06:38, sunrise at 07:09, moonrise at 09:59, sunset at 17:26, dusk at 17:56, and moonset at 23:50. On Feb. 14, dawn occurred at 06:37, sunrise at 07:08, moonrise at 10:37, and sunset at 17:27.

Power Output, Year 1 Run 4

Initial resistance values for Panel A were in the kΩ range, but were not recorded. Resistance measurements were taken at 15:36 on Feb. 15 after the voltage data were collected. Panel A circuit resistance was 0.279 MΩ and a voltage of –0.0706 V was measured; Panel B circuit resistance was 1.609 MΩ and a voltage of 0.1807 V was measured. For Panel A (rochelle salt-water), with measured voltage = 0.07 V, the initial calculated current for Panel A (I = V/R) is about 0.3 µA and calculated power (P = VI) is about 20 nW. For Panel B (water-water), with measured voltage = 0.18 V, the initial calculated current for Panel B (I = V/R) is about 0.1 µA and calculated power (P = VI) is about 20 nW.

Additional Observations, Year 1 Run 4

Audible fracture of the bottom layer occurred when the top layer was poured in place to freeze for both panels.

As with earlier runs, pitting and loss of surface transparency developed in the panel with the solute added. A crystalline film of exsolved solute was clearly visible on the surface. This film was scraped off at 14:00 on Feb. 14 to improve transparency.

As with earlier runs, a surface bulge developed in the water-water panel.

• Year 1 Run 5: rochelle salt-water and (acetic acid+iron)-ammonia prototypes, 22-23 February 2016.

Results from Year 1 Run 5 (Panel A: rochelle salt-water. Panel B: (acetic acid+iron)-ammonia) are given in Figure S9.

| 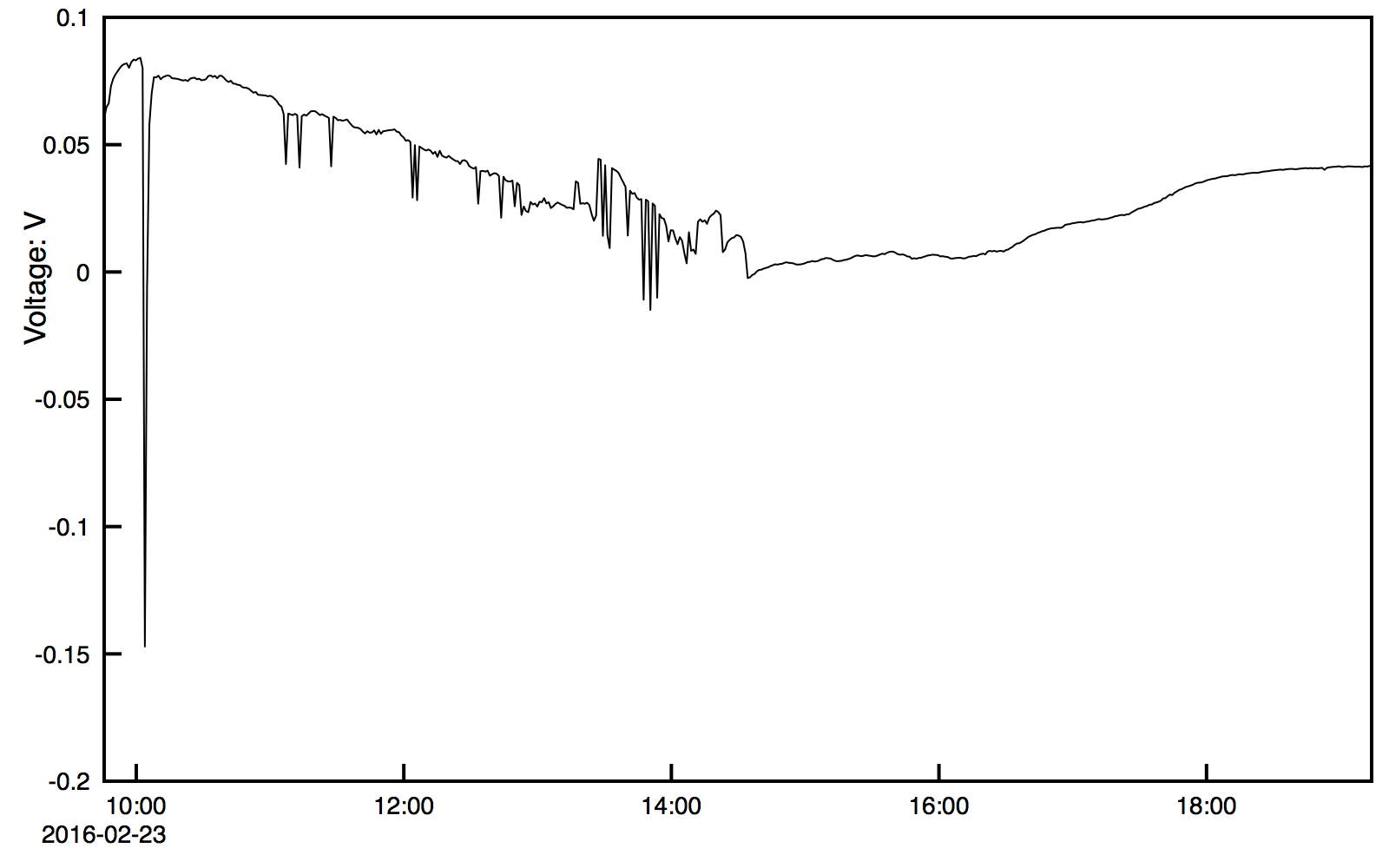 |
| --- |
| 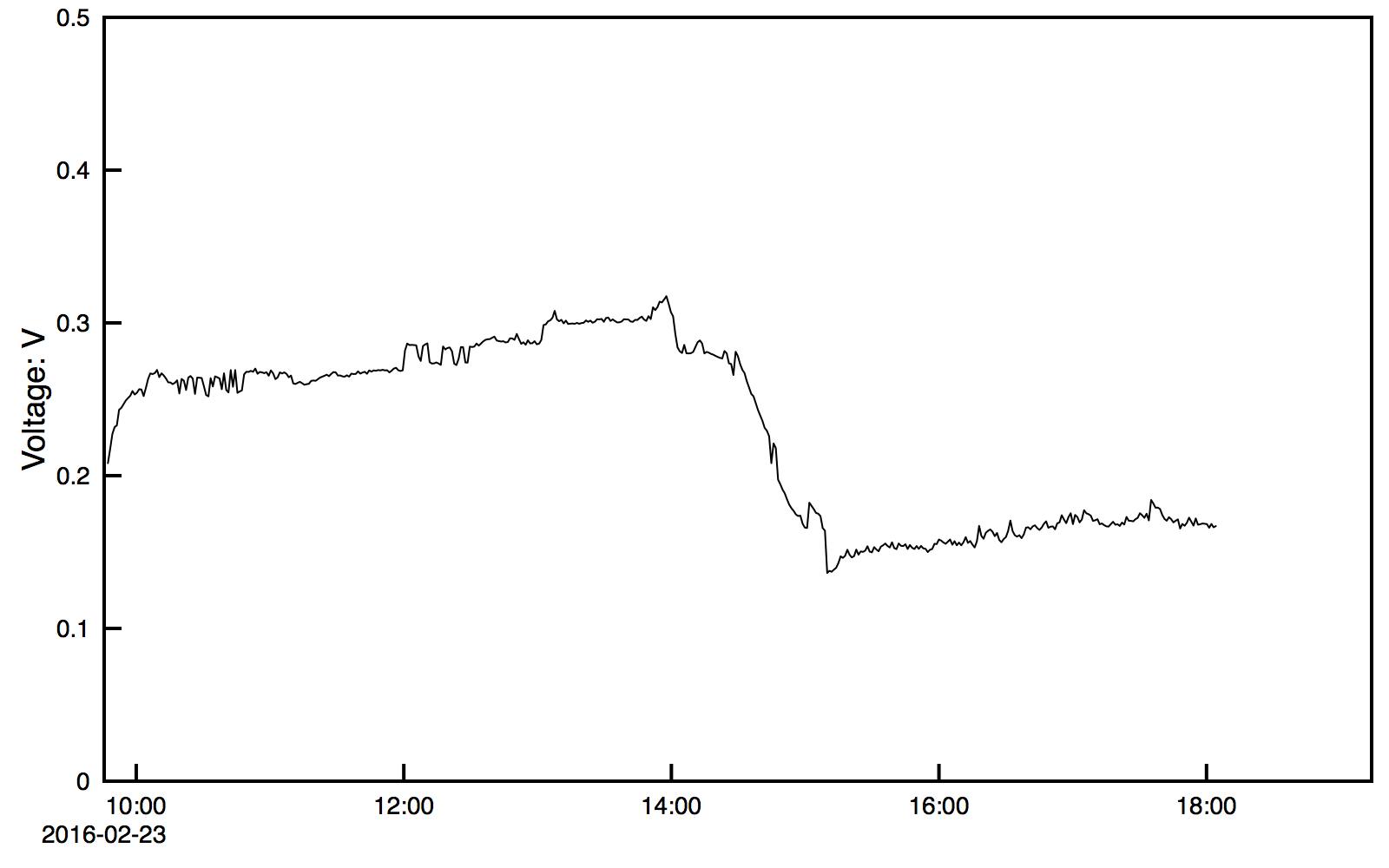 |
| 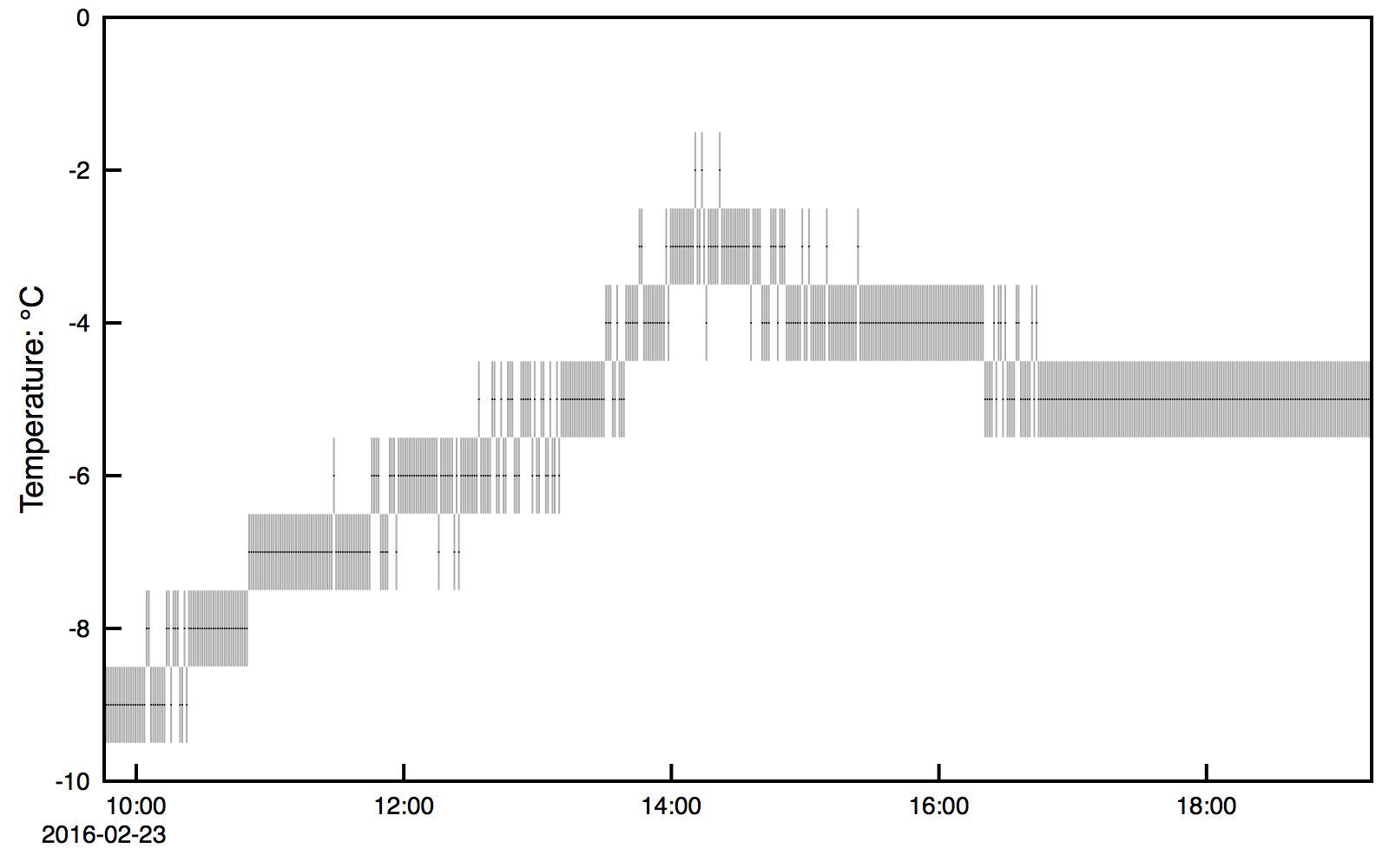 |
| 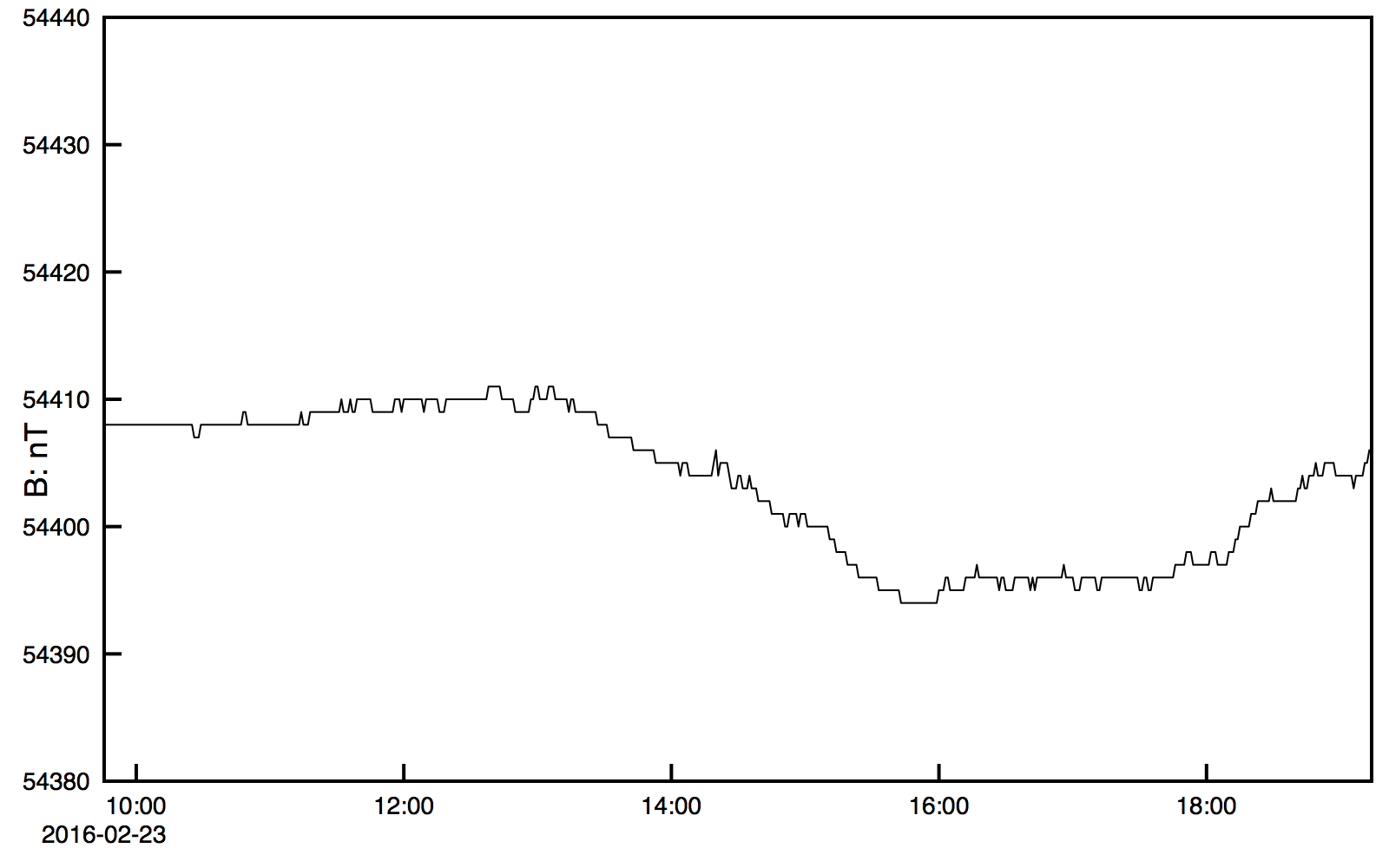 |
| 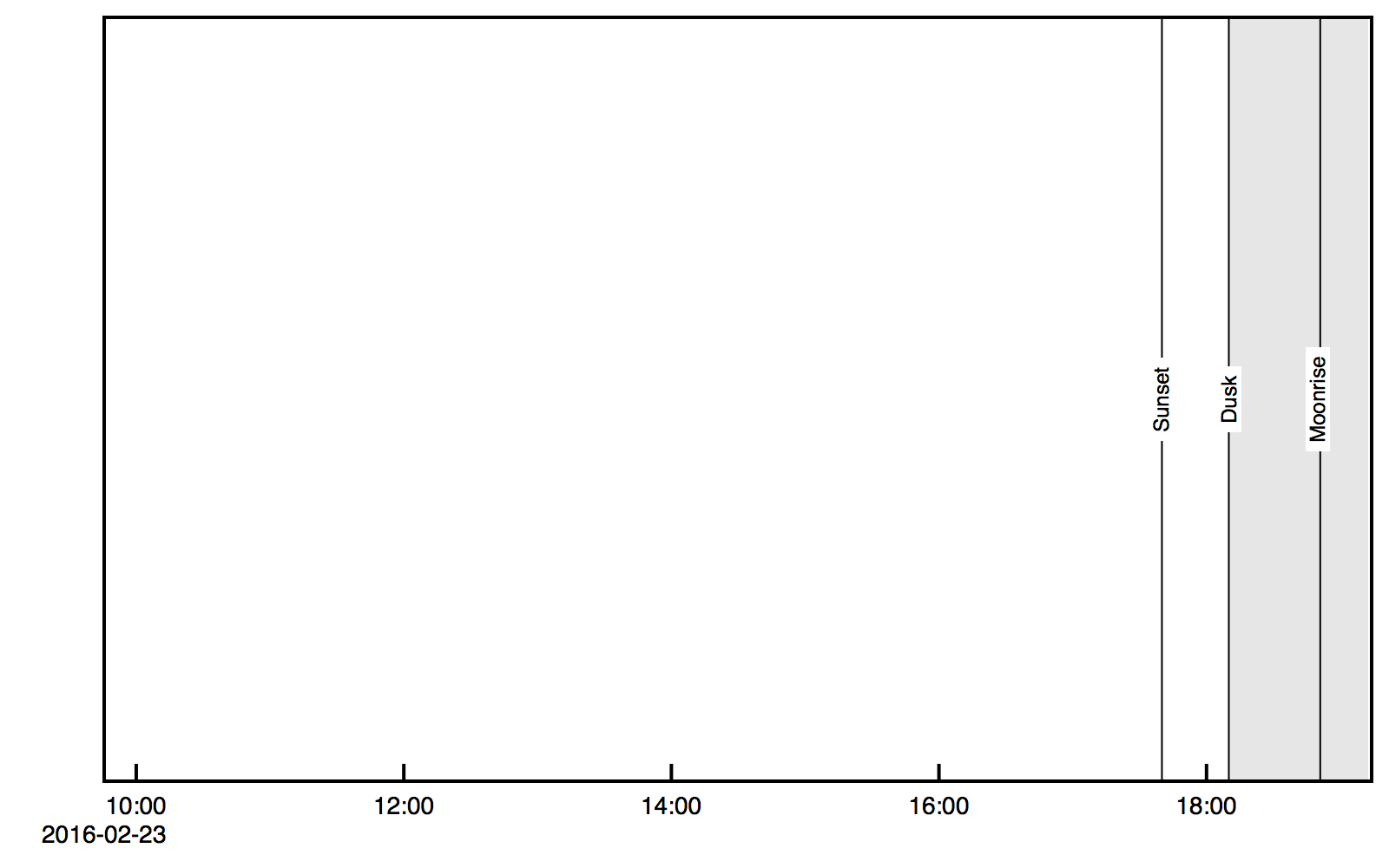 |

**Figure S9.** Data from Year 1 Run 5: 2016-02-23 09:45:37 to 2016-02-23 19:14:03 (Ottawa, ON, Canada, UTC–5). (a) Voltage: Panel A (rochelle salt-water).(b) Voltage: Panel B ((acetic acid+iron)-ammonia). Note that data from Panel B after 18:04:11 were lost due to a computer fault. (c) Temperature. (d) Total geomagnetic field strength. (e) Lunisolar data.

For Panel A (rochelle salt-water), voltage started at 0.06 V at 09:46 (Feb. 23) and rose (convex) until a large negative excursion at 10:04, and then was steady at 0.07 V and 0.08 V until 10:36. Then voltage fell (linear) until reaching 0.03 V at 12:52, and included several negative excursions. Then the voltage dropped to 0.02 V and stayed steady (with noise) until rising to 0.04 V (13:27) and then falling (linear, with negative excursions) until 0.01 V (14:08). Then there was a step up to 0.02 V (14:13) and a step down to 0.01 V (14:24), before a final step down to –0.002 V (14:34:13). Voltage then rose (sinusoid, with three cycles) smoothly to 0.04 V at the conclusion of the trial (19:14). For more detail: Voltage ranged from –0.0024 V (14:34:13) to 0.0841 V (10:01:51) with one large negative excursion that reversed polarity, from 0.0841 V (10:01:51) to –0.1471 V (10:03:52), and several smaller negative excursions until 14:34:14, including three (13:47:33, 13:50:35, 13:53:38) that also reversed polarity (–0.0109, –0.0149, –0.0101). There were no positive excursions recorded. There was also a longer period of negative polarity that started as a drop from 0.0074 V (14:33:13) to –0.0024 V (14:34:13) and continued from 14:34:13 to 14:37:16, rising (convex) steadily.

For Panel B ((acetic acid+iron)-ammonia), voltage was first measured at 0.21 V at 09:47 (Feb. 23) and rose (convex) to 0.26 V (09:58) and then rose (linear) to 0.32 V (13:58). Then there was a step down to 0.28 V, where it stayed from 14:04 to 14:30 before falling (linear) to 0.14 V (15:11). The measured voltage then rose (convex) to 0.17 V at 18:04. Data after 18:04:11 were lost due to a computer fault. For greater detail: Voltage ranged from 0.1363 V (15:09:53) to 0.3176 V (13:57:45) with two periods of frequent excursions of about 0.01 V, both positive and negative (10:19:20 to 10:48:23; and 12:00:32 to 12:29:37). Excursions were less frequent but common throughout. There were no reversals of polarity recorded.

Temperatures started at –9°C (09:46 Feb. 23) and rose (sinusoid) to –3°C (13:58) where they stayed until 14:51, with three excursions up to –2°C (14:11, 14:14, 14:22), and one excursion down to –4°C (14:16). After 14:51, temperatures dropped (concave) to –5°C through the end of the run (19:15). Temperatures ranged from –9°C to –2°C.

Total geomagnetic field strength started at ^54^ 408 nT at 09:47 (Feb. 23) and rose to ^54^ 411 nT (12:38). At 13:07, total field strength started to drop (linear) to ^54^ 394 nT (15:43), with two local maxima (^54^ 406 nT and ^54^ 400 nT) at 14:20 and 15:10, respectively. At 15:43, field strength started to rise (sinusoid, with four cycles) to ^54^ 406 nT through the end of the run at 19:14. The magnitude of the total geomagnetic field ranged from ^54^ 394 nT to ^54^ 411 nT.

On Feb. 23, sunset was at 17:40, and dusk was at 18:10. It was the day after the full moon, and moonrise occurred at 18:51.

Power Output, Year 1 Run 5

Resistance measurements were taken both before and after the voltage time series for each panel. For Panel A (rochelle salt-water), initial resistance values for 22 measurements (09:21:09 to 09:42:27) averaged 104.63 kΩ (standard deviation: 6.03 kΩ), and were increasing from beginning to end. The last value was 112.45 kΩ. Taking the measured voltage of 0.06 V at 09:46, the initial calculated current for Panel A (I = V/R) is about 0.5 µA and calculated power (P = VI) is about 30 nW. Final resistance values for 48 measurements (19:18:10 to 20:05:49) of Panel A averaged 133.83 kΩ (standard deviation: 14.65 kΩ), and were increasing from beginning to end. The first value was 74.72 kΩ. Taking the measured voltage of 0.04 V at 19:14, the final calculated current for Panel A (I = V/R) is about 0.5 µA and calculated power (P = VI) is about 20 nW.

For Panel B ((acetic acid+iron)-ammonia), initial resistance values for 22 measurements (09:21:35 to 09:42:39) averaged 155.00 Ω (standard deviation: 35.70 Ω), and were increasing from beginning to end. The last value was 196.41 Ω. Taking the measured voltage of 0.21 V at 09:47, the initial calculated current for Panel B (I = V/R) is about 1 mA and calculated power (P = VI) is about 0.2 mW. Final resistance values for 62 measurements (20:09:03 to 21:11:12) of Panel B averaged 565.31 Ω (standard deviation: 1.4047 kΩ), and were noisy. The first value was 190.16 Ω. Taking the last measured voltage of 0.17 V at 18:04, the final calculated current for Panel B (I = V/R) is about 0.9 mA and calculated power (P = VI) is about 0.2 mW.

Additional Observations, Year 1 Run 5

The bottom layer of Panel B ((acetic acid+iron)-ammonia) showed some billowing of frozen ammonia near where the lead is connected to the electrode. See Figure S10.

Adding the liquid water and rochelle salt at –12°C caused a fair bit of audible fracture in the distilled water layer below in Panel A. Adding vinegar caused no audible fracture in Panel B, but initially the new liquid was added in just one spot, and it may have melted the ammonia layer there somewhat.

As an attempt was made to tilt up the panels, Panel A (rochelle salt-water) ponded a little. In Panel B ((vinegar+iron)-ammonia) the vinegar layer ponded a lot, with maybe 500ml along the bottom, and was streaming out the drain holes. The panels were laid flat again.

At about 15:00, substantial leakage of vinegar (observable smell and evacuated aspect of the ice) was noted in Panel B. The surface of Panel B was white/opaque. The drain holes were then sealed with duct tape to limit additional losses. At 10% acetic acid the top layer did not freeze up completely. The top layer presented a lattice of ice and intercrystalline spaces filled with unfrozen vinegar.


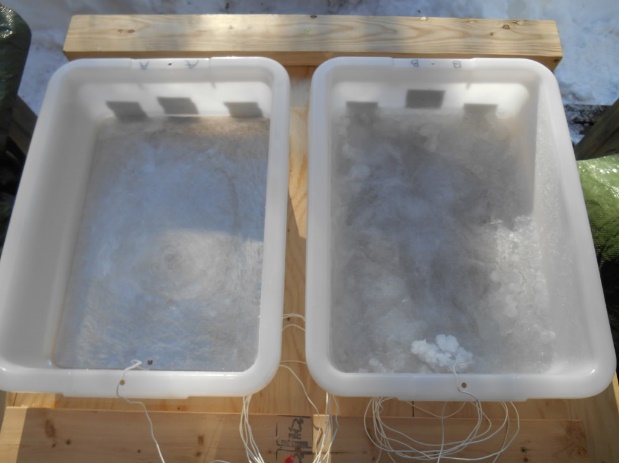


**Figure S10.** Bottom layers of Year 1 Run 5. Billowing of frozen ammonia is visible as a white patch of Panel B (right) near the electrode connection.

• Year 1 Run 6: (Acetic acid+iron)-water prototype, 1-5 March 2016.

Results from Year 1 Run 6 (Panel A: (acetic acid+iron)-water).

A mistake was made in designing the circuit to measure voltage concurrent with resistance. The data gathered thus were not usable.

Observations from Year 1 Run 6

Ammonia/ice again precipitated above the surface of the bottom layer as it was freezing, this time forming around the wire again, and climbing up around the panel edges.

After more than ten hours in preparation, the surface of the top layer was still shiny with liquid, which was an exsolute of more concentrated acetic acid from the freezing process. A large number of bubbles also formed in the top layer.

On March 6, there were clear blue skies and warm at 6°C by 15:15. There was significant panel deterioration, and the top electrode was exposed in part above the panel surface. There were still bubbles extant in the ice underneath. Oxidation of iron was observed in more areas and greater in extent.

S2.2 Year 2

• Year 2 Run 1: (Kaolinite+sucrose)-acetic acid-limestone suspension prototypes. 30 January to 3 February 2017.

Results from Year 2 Run 1 (Panels A and B: (kaolinite+sucrose)-acetic acid-limestone suspension) are given in Figure S11. Note that a 1 kΩ resistor was connected in parallel throughout the voltage measurements in order to calculate current.

| 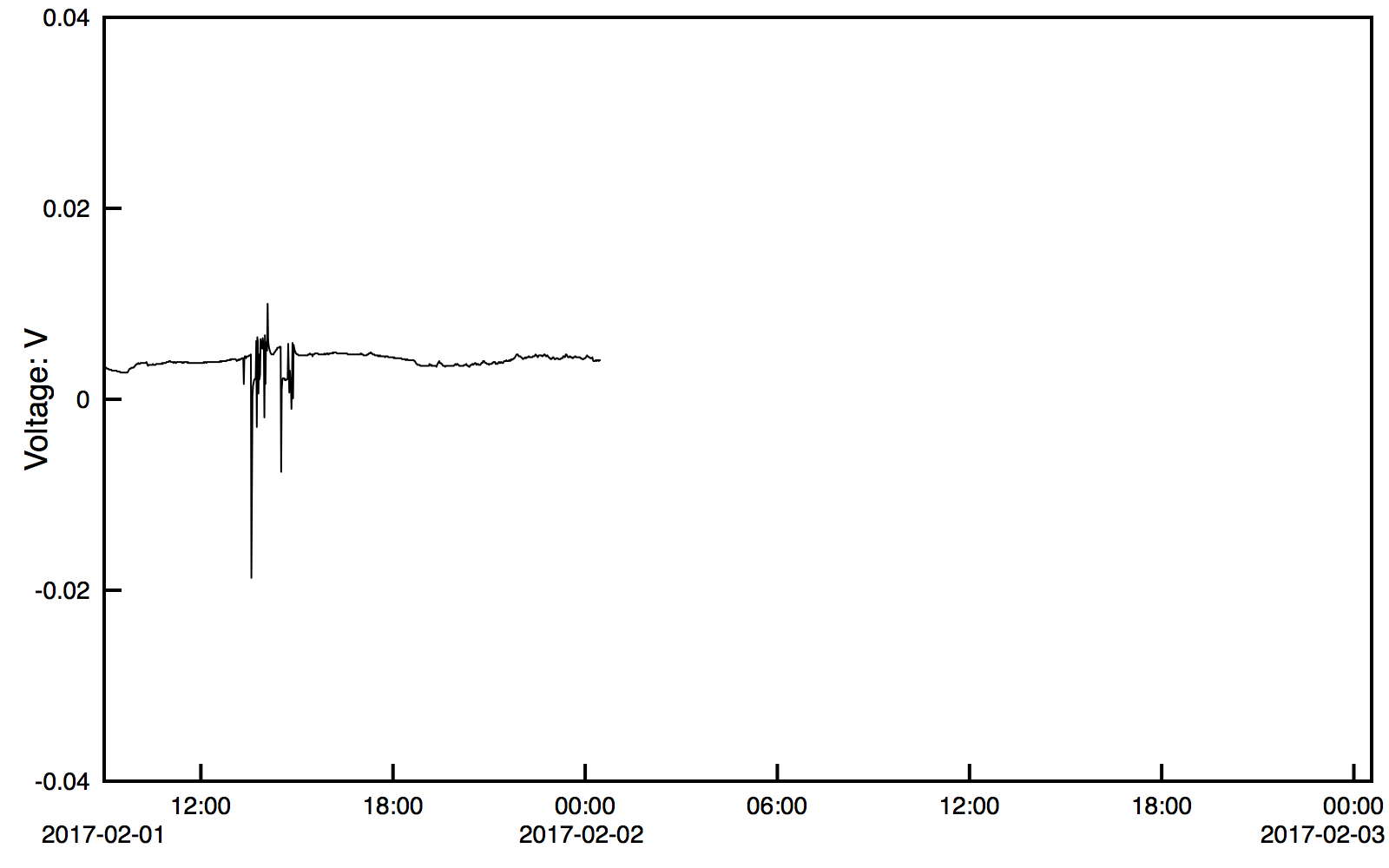 |
| --- |
| 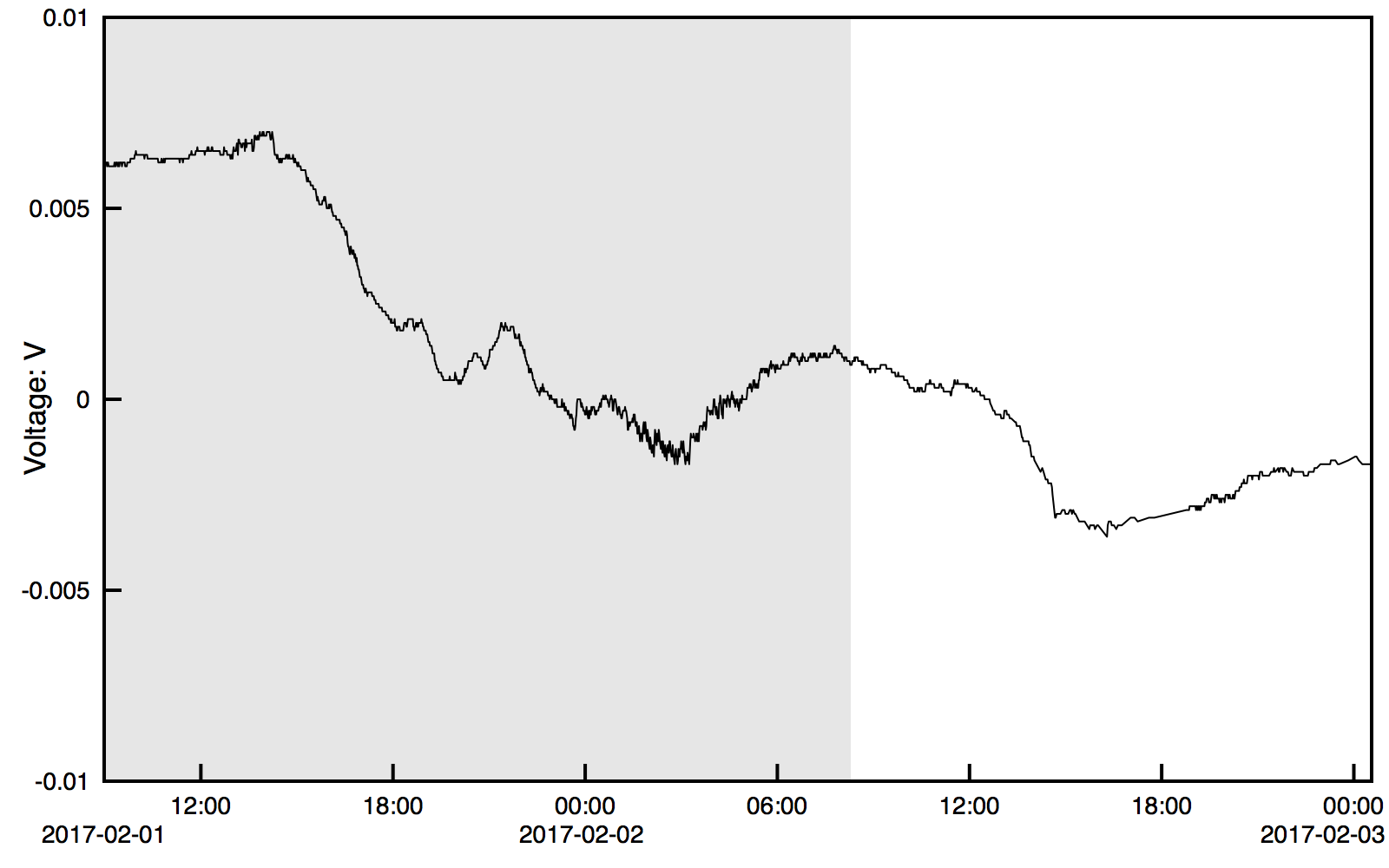 |
| 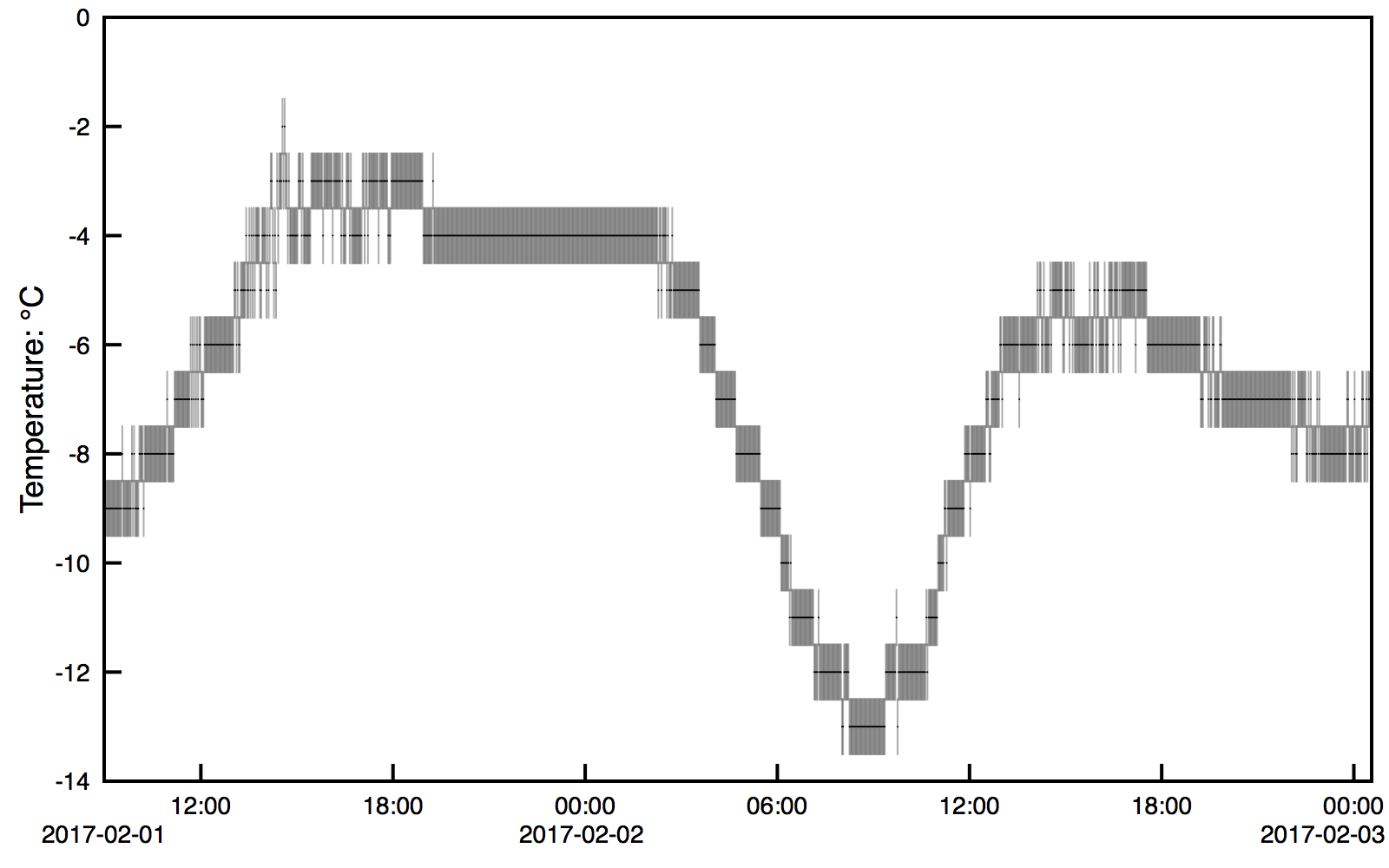 |
| 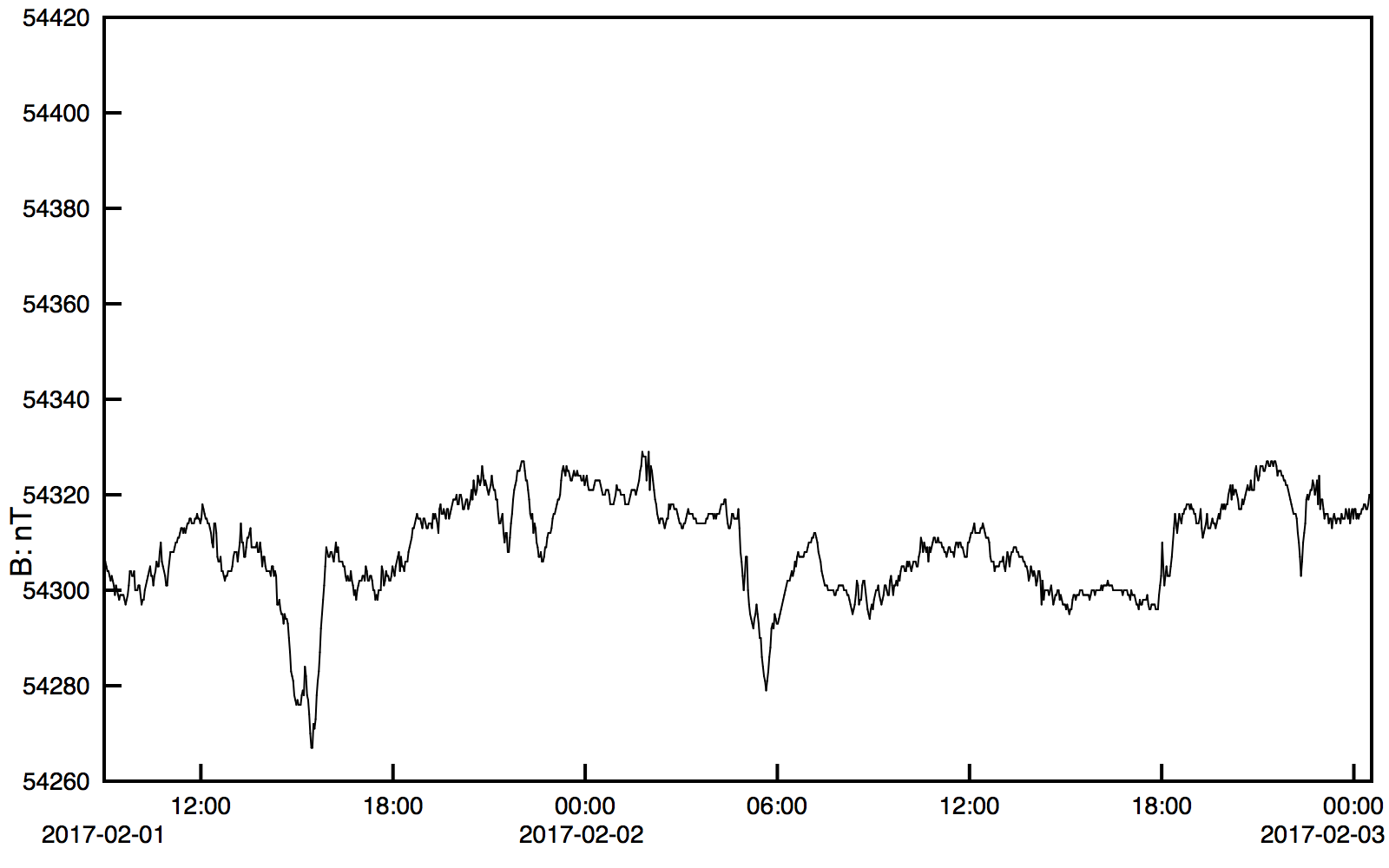 |
| 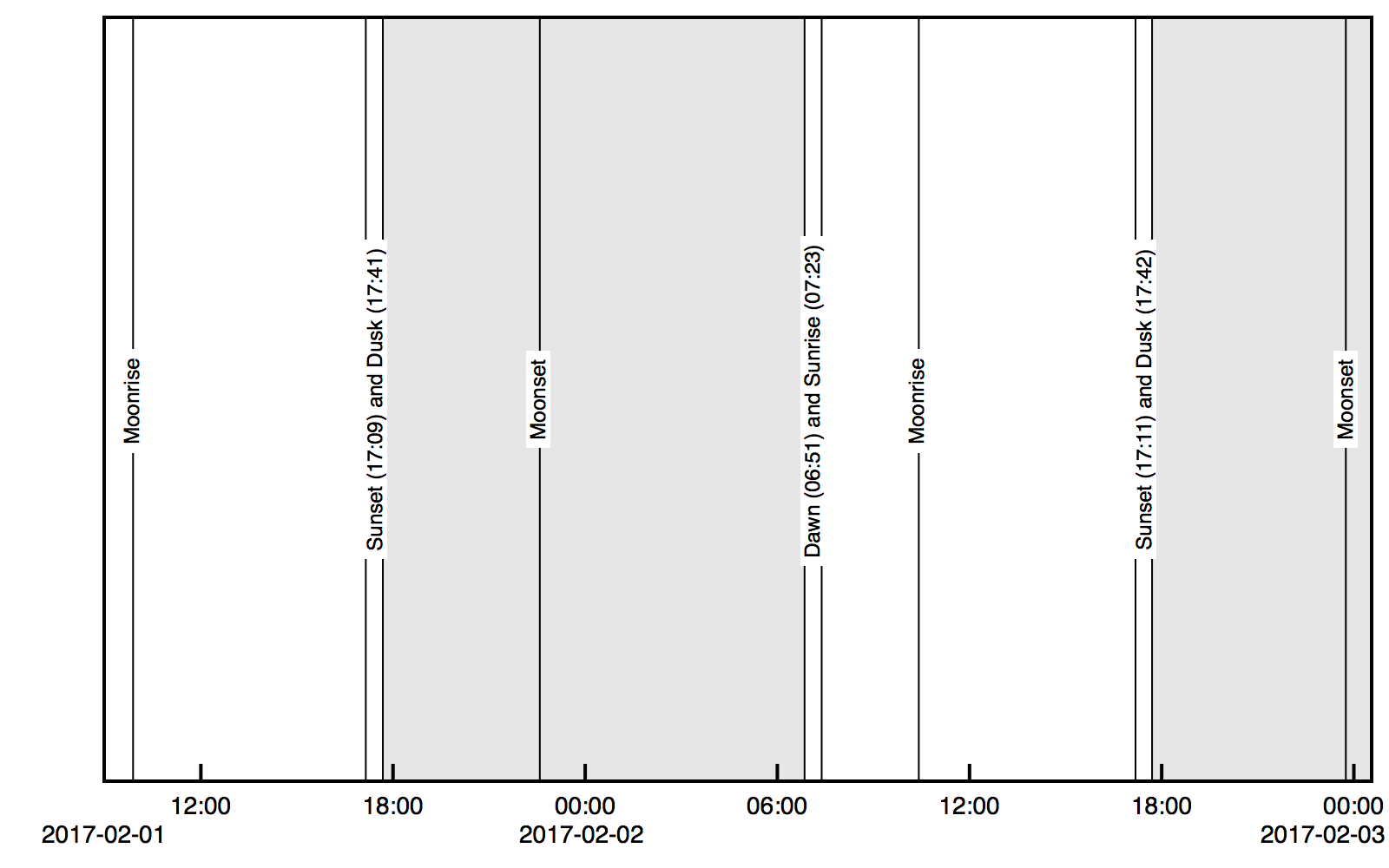 |

**Figure S11.** Data from Year 2 Run 1: 2017-02-01 08:58:53 to 2016-02-03 00:33:20 (Ottawa, ON, Canada, UTC–5). Prototype panels from (kaolinite+sucrose)-acetic acid-limestone suspension, with 1 kΩ resistor connected in parallel to allow for current measurements. (a) Voltage: Panel A, exposed from start to end. (b) Voltage: Panel B, covered at start / exposed 08:00 Feb. 2 until end. Note: Voltage data from Panel A end at 00:27:43 on Feb. 2 due to a battery failure in the DMM. Voltage data from Panel B become less regular (gaps of several minutes up to an hour) starting at 12:00:59 on Feb. 2 due to a battery fault in the DMM. (c) Temperature. (d) Total geomagnetic field strength. (e) Lunisolar data.

For Panel A ((kaolinite+sucrose)-acetic acid-limestone suspension, exposed start), voltage started at 0.004 V at 08:59 (Feb. 1) and fell and rose (sinusoid, four and a half cycles) from 0.003 V (09:29 Feb. 1) to 0.006 V (14:27 Feb. 1), and ended at 0.004 V on 00:28 Feb. 2. There were excursions up to 0.01 V (14:05) and down to –0.02 V (13:35) during a noisy interval from 13:20 to 14:57. For more detail: Voltage ranged from 0.0028 V (09:29:18 Feb. 1) to 0.0055 V (14:27:25 Feb. 1) with an interval of several small negative excursions from 13:20:30 Feb. 1 (0.0016 V) to 14:52:46 Feb. 1 (0.0001 V) including a larger one (–0.0187 V) on 13:34:41 that reversed polarity, plus one positive excursion to 0.0100 V (14:05:06). Note: Seventeen corrections were made to the data where the DMM had switched units during logging, i.e. isolated peak values were divided by 100 where nearest neighbors matched the new value and there were only two digits recorded instead of four.

For Panel B ((kaolinite+sucrose)-acetic acid-limestone suspension, covered start), voltage started at 0.006 V at 08:59 (Feb. 1) and rose (sinusoid, three cycles) to 0.007 V (13:59). Voltages then fell (linear, noisy with a local maximum at 0.002 V at 21:22) to –0.002 V (02:46 Feb. 2). Voltage then rose (convex) to 0.001 V (07:46) and the panel was then uncovered (08:00 Feb. 2). Voltage fell (convex) to –0.004 V (16:18) before rising (sinusoid, three cycles) to end the trial at –0.002 V at 00:33 Feb. 3. For more detail: Voltage ranged from –0.0036 V (16:17:36 Feb. 2) to 0.0070 V (13:50:33 Feb. 1) with a drop in voltage from 0.0070 starting at 14:13:35 Feb. 1 and attaining negative polarity at 23:02:32. Polarity stayed negative until the end of the trial but for a brief period from 00:33:40 Feb. 2 to 00:38:40 Feb. 2 where voltages attained 0.0001 V, and a longer period from 04:12:59 Feb. 2 to 12:27:10 Feb. 2 where voltages rose (convex) to 0.0014 V (07:46:17 Feb. 2) and then fell (convex) to –0.0036 V (16:17:36 Feb. 2). There were no large-magnitude excursions, but the voltage was noisy throughout and especially from 23:51:31 Feb. 1 to 07:46:17 Feb. 2. It was during the longer interval of positive polarity described above that the panel was exposed.

Temperatures started at –9°C (08:59 Feb. 1) and rose (concave) to –3°C (14:11) with two excursions up to –2°C (14:33, 14:37). Temperatures fell (concave) to –4°C, where they stayed from 18:52 until 02:40 Feb. 2, then dropped (linear) to –13°C at 08:01 before rising again (sinusoid) at 09:21 to –5°C at 14:06. Temperatures fell (concave) again starting at 17:32 to –8°C at the run's end at 00:33 Feb. 3. Temperatures ranged from –13°C to –2°C.

Total geomagnetic field strength started at ^54^ 307 nT at 08:59 (Feb. 1) and cycled between intervals as low as ^54^ 294 nT (08:53 Feb. 2) and as high as ^54^ 329 nT (01:47 Feb. 2) with two deep excursions to values as low as ^54^ 267 nT (15:27 Feb. 1) and ^54^ 279 nT (05:39 Feb. 2). The magnitude of the total geomagnetic field ranged from ^54^ 267 nT to ^54^ 329 nT.

On Feb. 1, dawn and sunrise occurred before the start of the run, moonrise was at 09:53, sunset was at 17:09, dusk was at 17:41 and moonset was at 22:35. On Feb. 2, dawn was at 06:51, sunrise was at 07:23, moonrise was at 10:25, sunset was at 17:11, dusk was at 17:42, and moonset was at 23:45. The run ended at 00:33 on Feb. 3.

Power and Energy Output, Year 2 Run 1

During the preliminary interval, Panel A was covered and B was exposed. At 22:00 on Jan. 30, initial readings for Panel B (exposed) were V = 0.01 mV and R = 0.34 Ω. The initial calculated current for Panel B (I = V/R) is about 30 mA and calculated power (P = VI) is about 0.3 nW. The initial resistance of Panel A (covered) was 0.37 Ω but voltage was below the detection limit of the DMM.

The voltage was measured using resistors of known value at 11:50, 13:45 and 20:30 on 2017-01-31. Each set of measurements took approximately 30 minutes to complete. The resistors with which maxima occurred are listed in Table S4. Note the general trend of diminishing values over time. Note also that at 10:50 for Panel A and at 20:30 for Panel B, respectively, voltage values were below the detection limit for 1 Ω resistance.

**Table S8.** Resistors of Output Maxima for (Kaolinite+Sucrose)-Acetic Acid-Limestone Suspension Prototype.

| **Value** | **Output 11:50** | **Resistor** | **Output 13:45** | **Resistor** | **Output 20:30** | **Resistor** |
| --- | --- | --- | --- | --- | --- | --- |
| V_A_ max | 0.1578 V | 1M Ω | 0.1568 V | 1M Ω | 0.1347 V | 1M Ω |
| V_B_ max | 0.4711 V | 1M Ω | 0.3627 V | 1M Ω | 0.2325 V | 1M Ω |
| I_A_ max | 20 μA | 5 Ω | 100 μA | 1 Ω | 100 μA | 1 Ω |
| I_B_ max | 100 μA | 1 Ω | 100 μA | 1 Ω | 20 μA | 5 Ω |
| P_A_ max | 0.206 μW | 100k Ω | 0.139 μW | 100k Ω | 0.100 μW | 100k Ω |
| P_B_ max | 2.81 μW | 1k Ω | 4.52 μW | 1k Ω | 0.548 μW | 1k Ω |

For context: At 11:50 before starting with the resistance box, voltage was below the detection limit on panels A and B. The temperature was –9°C and there was ambient shade due to cloud cover. Measurements were taken from 11:50 to 12:21. Once the resistors were removed, voltage again dropped down to below the detection limit. At 13:45 on Jan. 31, the exposed panel was under direct sunlight. The temperature was –6°C. Measurements were taken from 13:47 to 14:17. At 20:30 on Jan. 31 after dark the temperature was –8°C. Measurements were taken from 20:33 to 21:04.

On Feb. 1 at 08:55, additional voltage measurements were taken with 1 kΩ resistor in parallel. Panel A was exposed and directed toward the Sun. Panel B was covered. For Panel A, with measured voltage = 0.0037 V, the initial calculated current (I = V/R) is about 3.7 μA and calculated power is about 14 nW. For Panel B, with measured voltage = 0.0062 V, the initial calculated current (I = V/R) is about 6.2 μA and calculated power is about 38 nW.

Additional Observations, Year 2 Run 1

Kaolinite and powdered limestone did not stay in suspension. They settled on the bottom of the top and bottom layers, respectively.

In transferring the top panels from the forms to the bus pans, no cracks, crevices or angular surface deformations were apparent. A bit of a trim to round off the corners was needed for fit.

Panels were assembled by around 22:00 on Jan. 30. At 11:30 on Jan. 31, ice was seen to be growing vertically around the perimeters of both panels, and some of this on thawing smelt weakly of acetic acid, i.e. from the middle layer. See Figure S12.

At 13:45 on Jan. 31, the surface of Panel A has a clear frost layer that is fragile to touch and has a mild acetic acid smell. Panel B appears to have regions where fluid has migrated out, leaving a snow-cone aspect in parts, i.e. with higher opacity where there are small air gaps.

| 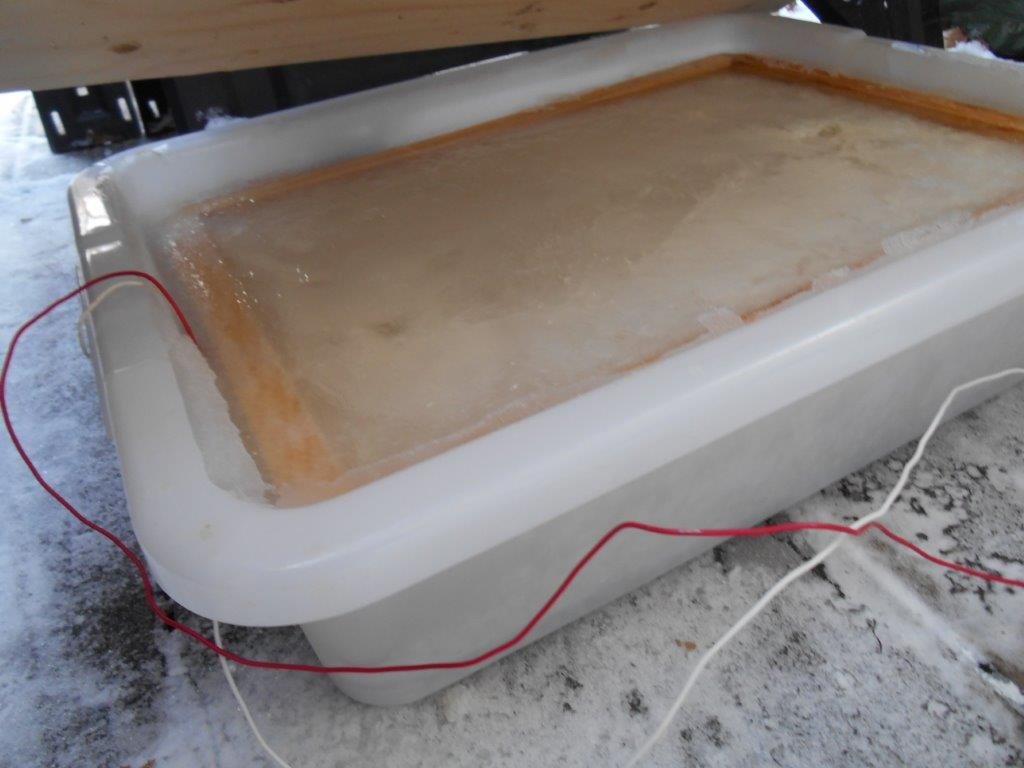 | 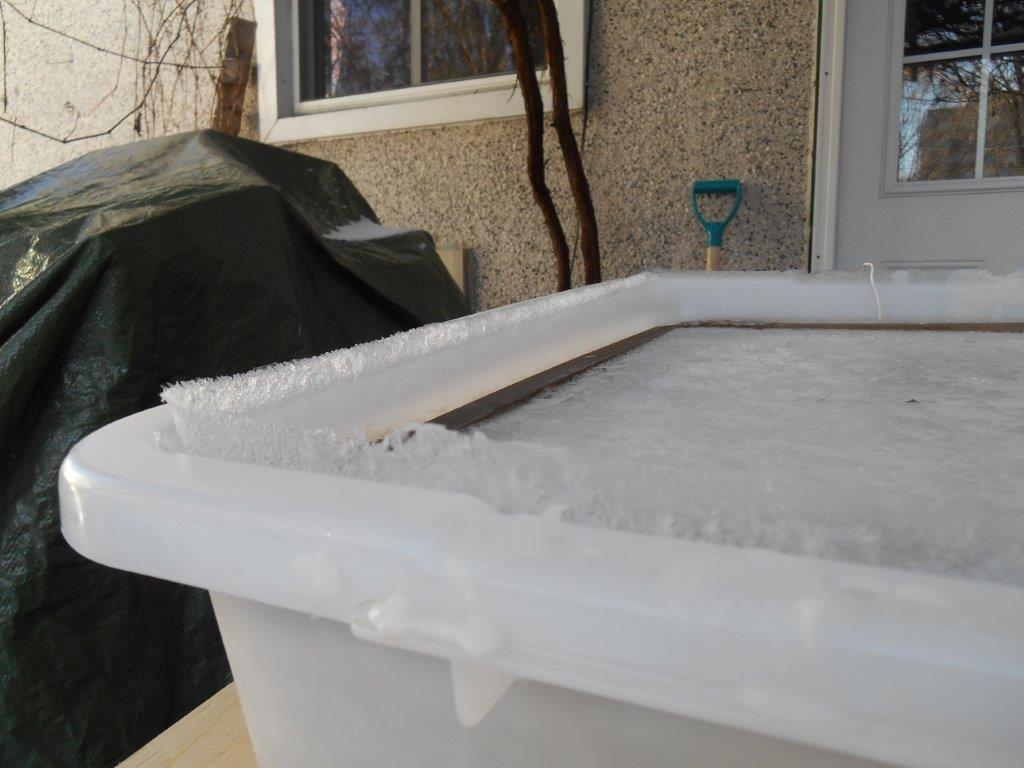 |
| --- | --- |

**Figure S12.** (a) Prototype Panel A ((kaolinite+sucrose)-acetic acid-limestone suspension) at 12:50 on Jan. 31. (b) Prototype Panel B (same composition as A) at 12:49 on Jan. 31. Note the additional ice present at the margins in both panels and especially above the walls of the bus pan at the perimeter in Panel B.

• Year 2 Run 2: (Kaolinite+NaCl)-HCl-MKP prototypes, 3-14 March 2017.

Results from Year 2 Run 2 (Panels A and B: (kaolinite+NaCl)-HCl-MKP) are given in Figure S13. All times given are standard time not daylight savings time. Note that a 1 kΩ resistor was connected in parallel throughout the voltage measurements in order to calculate current.

| 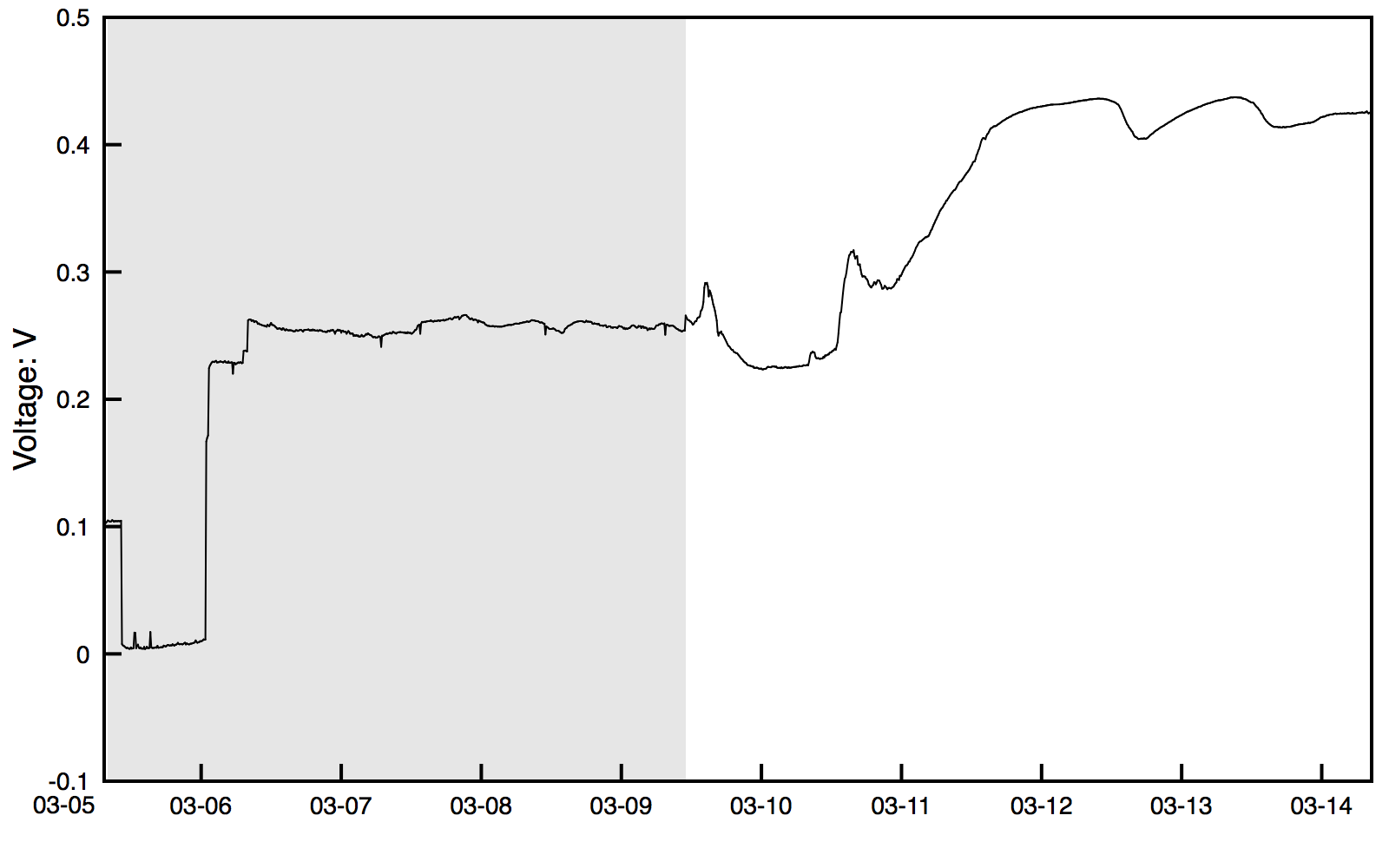 |
| --- |
| 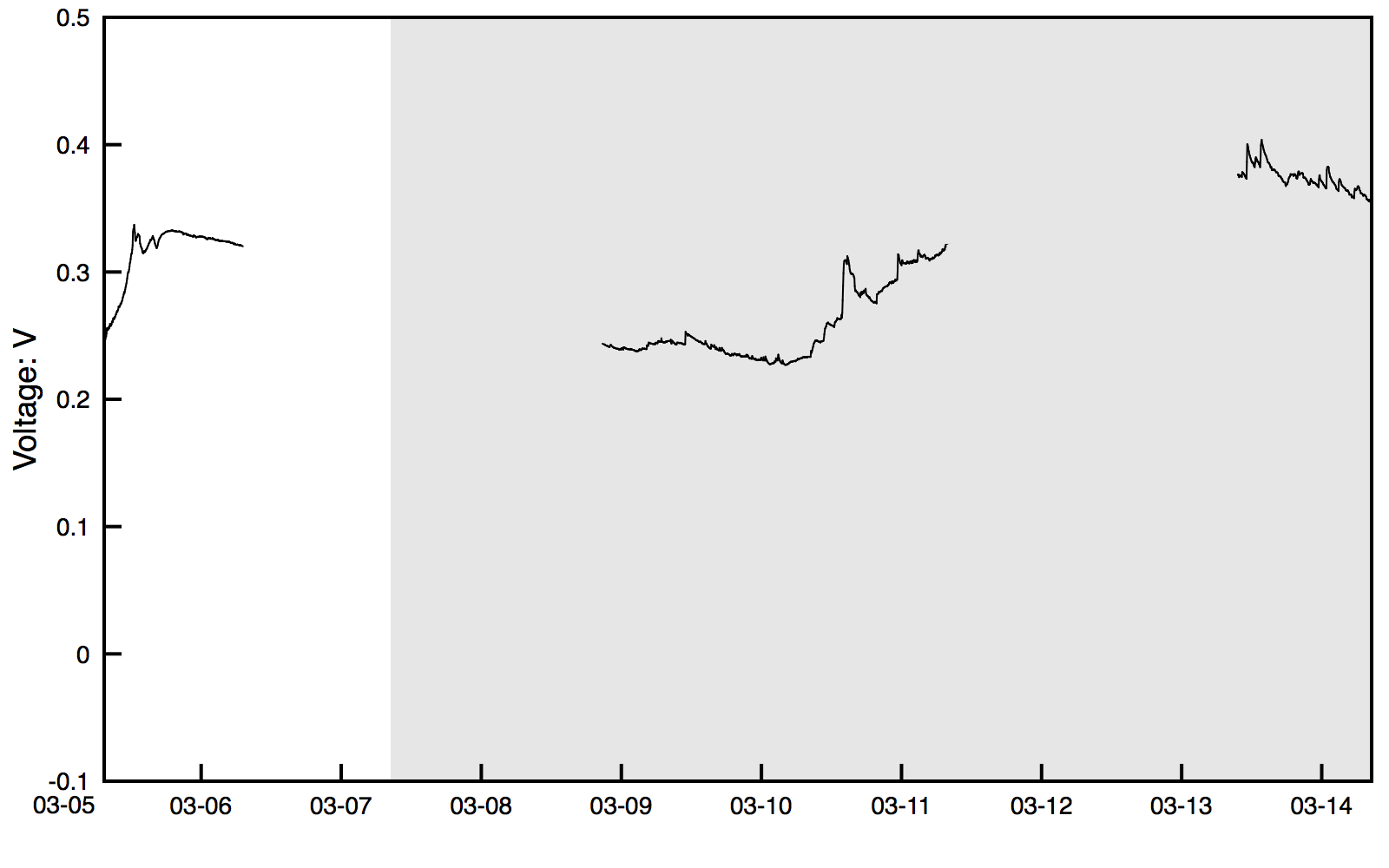 |
| 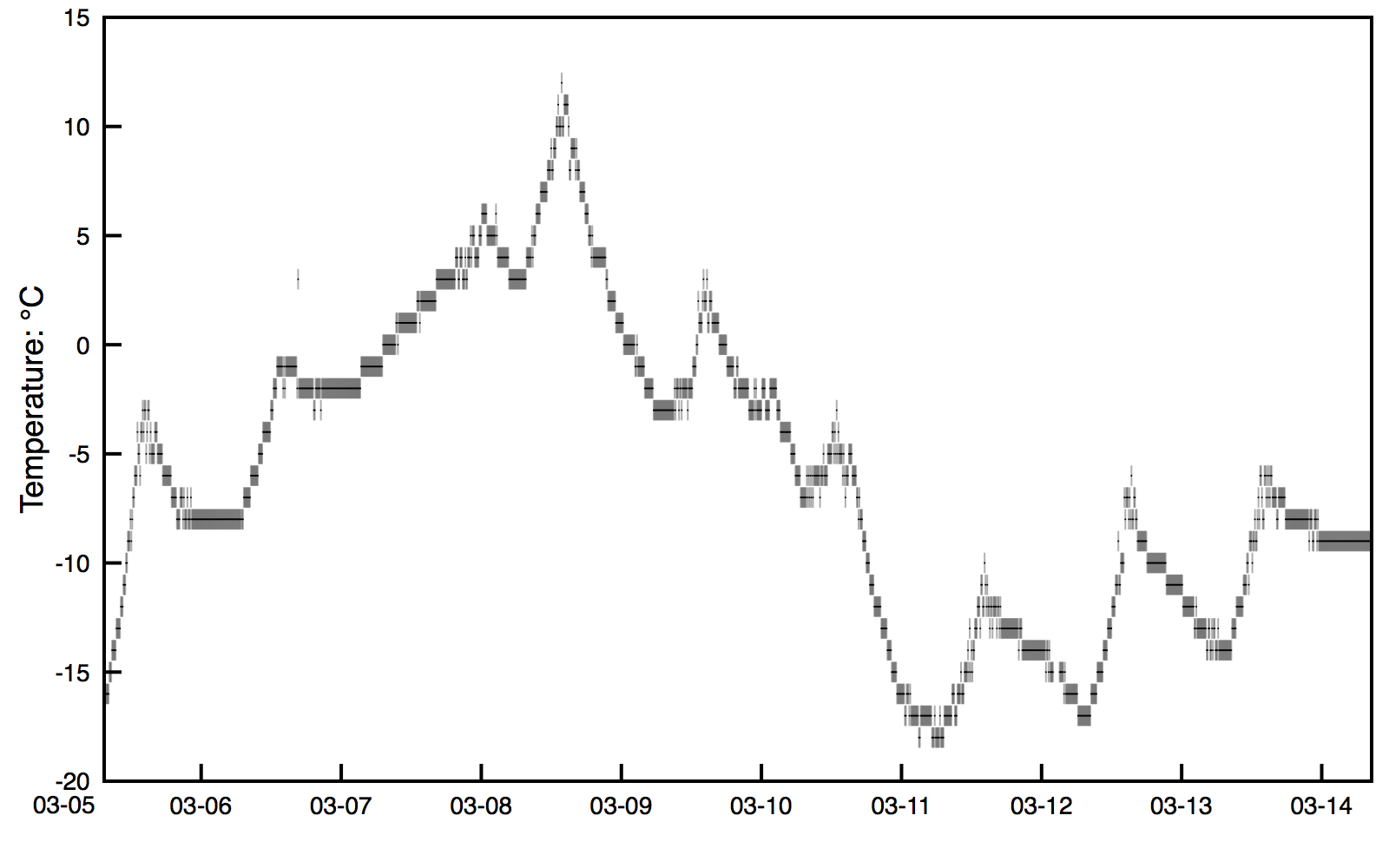 |
| 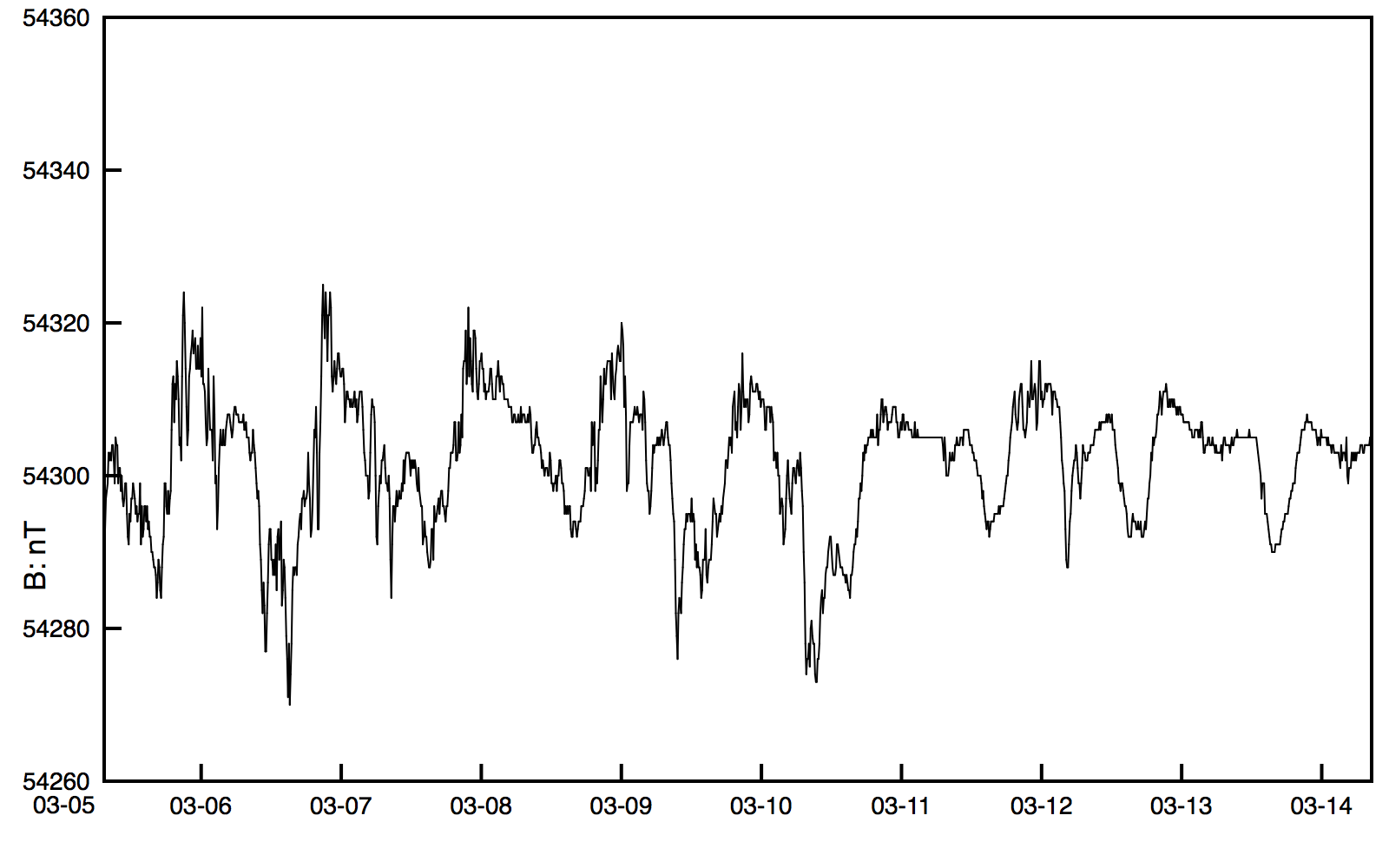 |
| 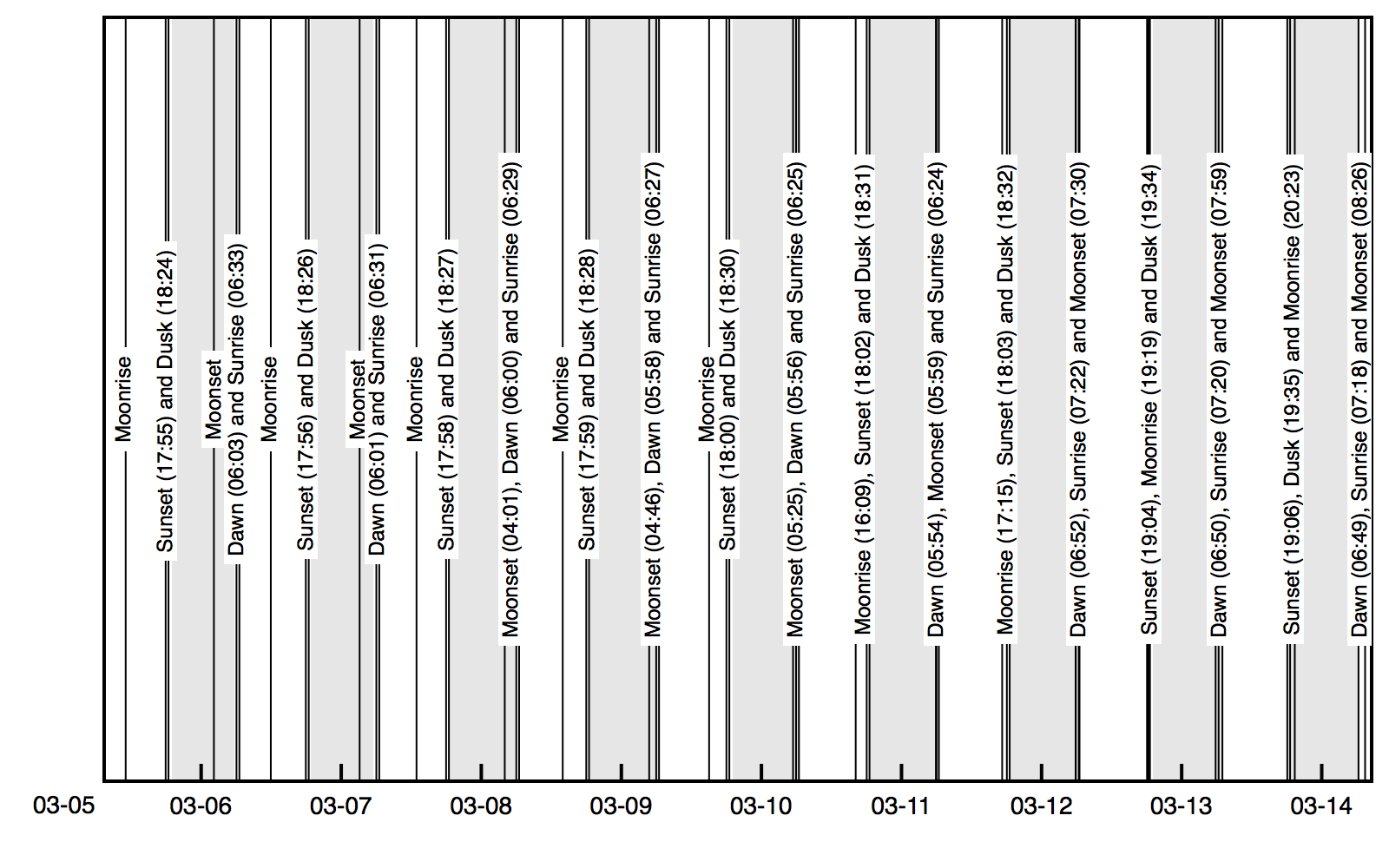 |

**Figure S13.** Data from Year 2 Run 2: 2017-03-05 07:22:52 to 2016-03-14 08:33:08 (Ottawa, ON, Canada, UTC–5. All times are standard time, not daylight savings time). Prototype panels from (kaolinite+NaCl)-HCl-MKP, with 1 kΩ resistor connected in parallel to allow for current and power calculations. (a) Voltage: Panel A (covered at start / exposed 11:00 Mar. 9 until end). (b) Voltage: Panel B (exposed at start / covered 07:10 Mar. 7 until end). Notes: Both panels were covered on Mar. 7 at 07:10 because of precipitation (freezing rain). Data for Panel B are missing from 06:56:03 Mar. 6 to 21:01:45 Mar. 8, and from 08:10:18 Mar. 11 to 09:32:38 Mar. 13 due to a computer fault. (c) Temperature. (d) Total geomagnetic field strength. (e) Lunisolar data.

For Panel A ((kaolinite+NaCl)-HCl-MKP, covered at start, exposed from 11:00 Mar. 9 until end), voltage started at 0.0001 V at 07:23 (Mar. 5) and stepped up (square) to 0.10 V at 07:24, and then stepped down (square) to 0.010 V at 10:19, rising (concave) to 0.013 V at 00:48 Mar. 6. During this interval there were positive and negative excursions up to 0.14 V (18:50) and down to 0.0001 V (19:16). Voltage then stepped up (square) to 0.23 V (01:16) until stepping up again (square) to 0.26 V at 07:55. Voltage then varied (sinusoid, four cycles) from 0.25 V to 0.27 V until 10:58 Mar. 9. The panel was uncovered at 11:00 on Mar. 9. Voltage then rose (concave) to 0.29 V (14:39), fell (concave) to 0.22 V (22:36), rose (concave) to 0.24 V (12:48 Mar. 10), rose (convex) to 0.32 V (15:45), and then fell (concave) to 0.29 V (22:11) before rising (linear) to 0.41 V at 14:59 Mar. 11. Voltage then rose (convex) to two peaks (0.44 V 11:11 Mar. 12; and 0.44 V 09:55 Mar. 13) and fell (sinusoid/convex) to two valleys (0.40 V 16:46 Mar. 12; and 0.41 V 16:37 Mar. 13) before rising (sinusoid) and ending at 0.43 V at 08:33 Mar. 14. For more detail: Voltage ranged from 0.0001 V (07:23:22 Mar. 5) to 0.4373 V (08:56:31 Mar. 13) with small positive and negative excursions occurring especially occurring from 07:23:22 Mar. 5 to 00:44:45 Mar. 6. The square steps occurred during the time that the panel was shaded. There were no reversals of polarity during the trial. Note: Two-hundred-twenty-four corrections were made to the data where the DMM had switched units during logging, i.e. isolated peak values were divided by 100 where nearest neighbors matched the new values and there were only two digits recorded instead of four.

For Panel B ((kaolinite+NaCl)-HCl-MKP, exposed start, covered from 07:10 Mar. 7 until end), data are missing from 06:56:03 Mar. 6 to 21:01:45 Mar. 8, and from 08:10:18 Mar. 11 to 09:32:38 Mar. 13 due to two computer faults. Voltage started at 0.0003 V at 07:23 (Mar. 5) and stepped up (square) to 0.24 V at 07:24, and then rose (concave) to 0.33 V at 12:18 and then descended (concave, with initial noise) to 0.32 V at 06:56 (Mar. 6). Data logging stopped due to a computer fault, and during this time the panel was covered due to freezing rain, and then remained shaded for the rest of the trial. Data logging then resumed at 0.24 V at 21:02 on Mar 8. Voltage then fell (sinusoid, two cycles) to 0.23 V on 04:03 (Mar. 10) and then rose (concave, with one local maximum of 0.31 V at 14:42) to 0.32 V at 08:10 (Mar. 11). Data logging stopped due to a second computer fault, and then resumed at 0.38 V at 09:33 on Mar. 13. Voltage then rose (convex, noisy) to 0.40 V at 13:45, and then fell (concave, noisy) to 0.35 V at 08:32 (Mar. 14). For more detail: Voltage ranged from 0.2269 V (04:03:27 Mar. 10) to 0.4034 V (13:45:00 Mar. 13) with a single lower initial value of 0.0003 V (07:22:52 Mar. 5) at the beginning of the trial. Data were noisy throughout. There were no reversals of polarity during the trial.

Temperatures started at –17°C (07:23 Mar. 5) and rose (concave) to –2°C (13:54) and then fell (concave) to –8°C (19:33) where they stayed (except for one excursion to –9°C at 06:09 Mar. 6) until rising again starting at 07:26. Temperatures then rose (concave) to –1°C (12:47) before falling again to –2°C where they stayed until 03:22 (Mar. 7). Temperatures then rose steadily (convex) to 6°C at 00:06 (Mar. 8) and then dropped (convex) to 3°C (04:22). Starting at 07:36, temperatures rose (convex) to 12°C (13:34) and then fell (concave) to –3°C (05:27 Mar. 9). Temperatures then rose (convex) to 3°C (13:55) before descending (concave, with a local maximum of –3°C at 12:03 Mar. 10) to –18°C at 02:44 Mar. 11. Temperatures then rose through three peaks (–10°C at 13:43 Mar. 11; –6°C at 15:07 Mar. 12; and –6°C at 13:28 Mar. 13) and fell through three valleys (–17°C at 05:55 Mar. 12; –14°C at 04:10 Mar. 13; and –9°C at 20:21 Mar. 13). The temperature was still at –9°C at 08:33 Mar. 14 when the run ended. During the run, there were two intervals with positive temperatures: 09:20 Mar. 7 to 00:25 Mar. 9; and 12:58 Mar. 9 to 16:44 Mar. 9. In all, temperatures ranged from –18°C to 12°C.

Total geomagnetic field strength started at ^54^ 296 nT at 07:23 (Mar. 5) and cycled ten times between intervals as low as ^54^ 270 nT (15:11 Mar. 6) and as high as ^54^ 328 nT (21:53 Mar. 6). Ranges of values were at their more extreme from the start of the run until 17:20 (Mar. 10). At the end of the run, the total field strength value was ^54^ 304 nT (08:33 Mar. 14). The magnitude of the total geomagnetic field ranged from ^54^ 270 nT to ^54^ 328 nT.

On Mar. 5, moonrise was at 11:04, sunset was at 17:55, and dusk was at 18:24. On Mar. 6, moonset was at 02:11, dawn was at 06:03, sunrise was at 06:33, moonrise was at 11:56, sunset was at 17:56, and dusk was at 18:26. On Mar. 7, moonset was at 03:09, dawn was at 06:01, sunrise was at 06:31, moonrise was at 12:54. sunset was at 17:58, and dusk was at 18:27. On Mar. 8, moonset was at 04:01, dawn was at 06:00, sunrise was at 06:29, moonrise was at 13:57, sunset was at 17:59, and dusk was at 18:28. On Mar. 9, moonset was at 04:46, dawn was at 05:58, sunrise was at 06:27, moonrise was at 15:02, sunset was at 18:00, and dusk was at 18:30. On Mar. 10, moonset was at 05:25, dawn was at 05:56, sunrise was at 06:25, moonrise was at 16:09, sunset was at 18:02, and dusk was at 18:31. On Mar. 11, dawn was at 05:54, moonset was at 05:59, sunrise was at 06:24, moonrise was at 17:15, sunset was at 18:03, and dusk was at 18:32. Note, daylight savings time (DST) started in Canada on Mar. 12, but for consistency with the experiment, all times listed here for Mar. 12-14 are standard time, not DST. On Mar. 12, dawn was at 05:52, sunrise was at 06:22, moonset was at 06:30, sunset was at 18:04, moonrise was at 18:19, and dusk was at 18:34. The full moon also occurred on Mar. 12 at 09:55, but had not yet risen at that time. On Mar. 13, dawn was at 05:50, sunrise was at 06:20, moonset was at 06:59, sunset was at 18:06, dusk was at 18:35, and moonrise was at 19:23. On Mar. 14, dawn was at 05:49, sunrise was at 06:18, and moonset was at 07:26. The run ended on Mar. 14 at 08:33.

Power and Energy Output, Year 2 Run 2

The voltage was measured using resistors of known value at 10:55, 13:00, 17:10 and 19:50 on 2017-03-04. Panel A was covered, and Panel B was exposed with direct sun at 13:00, and cloudy otherwise. Each set of measurements took approximately 15 minutes to complete. The resistors with which maxima occurred are listed in Table S9. Current scale (amperes) for Panel B is 10x the scale for Panel A. Note the general trend of increasing voltage over time, and the mid-resistance peaks in voltage, current and power at 10:55 in Panel B and for both panels at 13:00.

**Table S9.** Resistors of Output Maxima for (Kaolinite+NaCl)-HCl-MKP Prototype.

| **Value** | **Output 10:55** | **Resistor** | **Output 13:00** | **Resistor** | **Output 17:10** | **Resistor** | **Output 19:50** | **Resistor** |
| --- | --- | --- | --- | --- | --- | --- | --- | --- |
| V_A_ max | 0.2049 V | 1M Ω | 0.2440 V | 1M Ω | 0.2521 V | 1M Ω | 0.2601 V | 1M Ω |
| V_B_ max | 0.2239 V | 1M Ω | 0.2535 V | 1M Ω | 0.2727 V | 1M Ω | 0.2984 V | 1M Ω |
| I_A_ max | 0.1 mA | 1 Ω | 1.05 mA | 100 Ω | 0.2 mA | 1 Ω | 0.2 mA | 1 Ω |
| I_B_ max | 13.2 mA | 10 Ω | 20.51 mA | 10 Ω | 3.9 mA | 1 Ω | 3.5 mA | 1 Ω |
| P_A_ max | 3.19 μW | 1k Ω | 110 μW | 100 Ω | 8.48 μW | 1k Ω | 8.56 μW | 1k Ω |
| P_B_ max | 1740 μW | 10 Ω | 4207 μW | 10 Ω | 194.6 μW | 100 Ω | 193.8 μW | 100 Ω |

For additional detail: At 10:55 on Mar. 4 with Panel A covered and Panel B in sunlight (indirect), the temperature was –18°C. Initial voltage readings without any resistor attached were Panel A = 0.049 V and Panel B = 0.18 V. Measurements were taken from 10:56 to 11:09. On removal of resistors, measured voltage was undetectable for Panel A, and 0.0004 V for Panel B. At 13:00 on Mar. 4 with Panel A covered and Panel B in sunlight (direct), the temperature was –12°C. Measurements were taken from 13:01 to 13:15. At 17:10 on Mar. 4 with Panel A covered and Panel B in sunlight (indirect), the temperature was –12°C. Measurements were taken from 17:10 to 17:24. At 19:50 on Mar. 4, the temperature was –12°C and dark from nightfall. Measurements were taken from 19:52 to 20:04.

Additional Observations, Year 2 Run 2

The middle layer with HCl was not a slurry like with acetic acid but was firmer.

On both Mar. 4 and 5, the weather was sunny and clear, with direct sun at about 13:00. On Mar. 6, there was grey, freezing rain starting around 16:00. On Mar. 8, the panels survived warm weather with only minimal melting around the margins; the freezing rain residue was still visible afterwards on Panel B, as shown in Figure S14. Panels are not frozen solid: the top layer can move side-to-side. On Mar. 9 at 11:00, the weather was mostly sunny. On Mar. 10, the weather was cloudy. The surface of Panel A had refrozen with an uneven, irregular texture and aspect, as shown in Figure S15. On Mar. 11 to 13, the weather was sunny and cold.


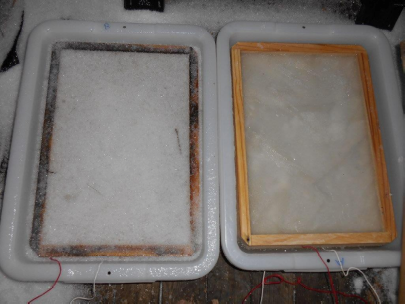


**Figure S14.** Prototype Panels A and B on March 8, 2017. Freezing rain residue is visible on Panel B.


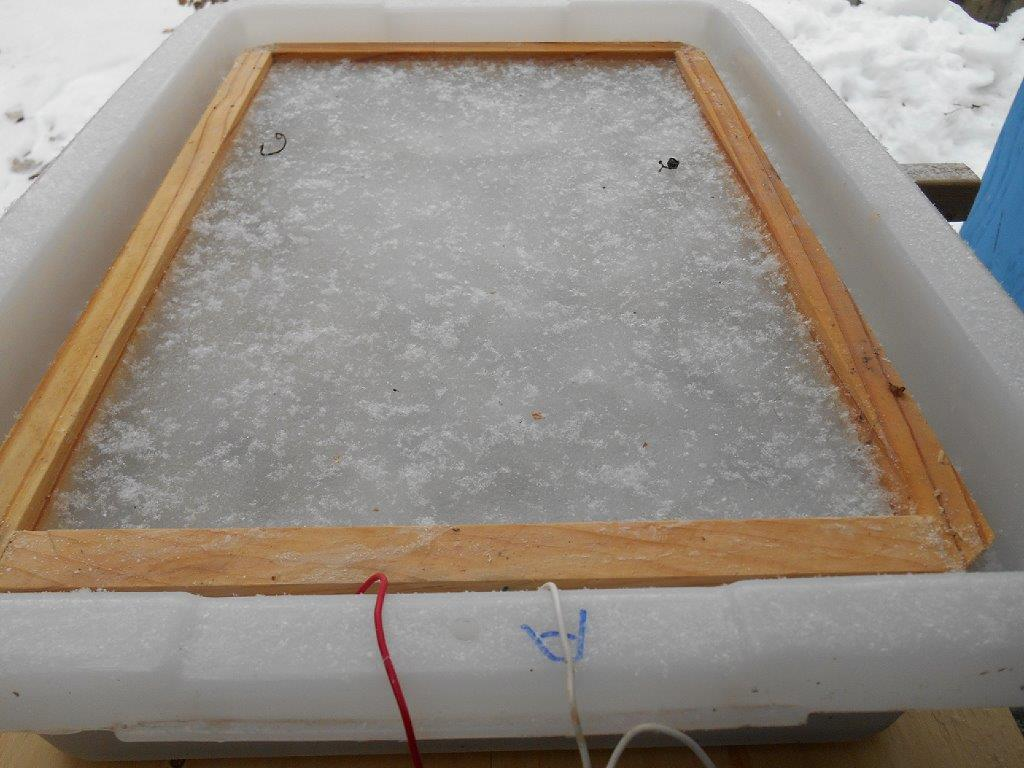


**Figure S15.** Panel A on 10 Mar. 2017 showing uneven, irregular top surface.

Table S10 provides a summary of the voltage, current and power data recorded or calculated in the first of two winter seasons, showing Experiments 1-4. The highest power outputs were in the first (ice-ice) panel and the last ((acetic acid+iron)-ice) panel. Voltage data are given as mean and median data over the entire dataset. Note that the current and power are calculated on one set of voltage and resistance measurement (E1, E2, E3T1), or in the case of the final run (E3T2, E4) on the mean of the two power values calculated from the initial and final voltage and resistance measurements. Initial resistance measurements were taken approximately one minute before voltage readings of the panels started, and final resistance measurements approximately one minute after. Table S11 lists the measured resistances and initial/final voltages. The formulas for power calculations are

*I* = *V*/*R* (1)

and

*P* = *VI* (2)

where *R*, *V*, *I* and *P* are resistance (Ω), voltage (V), current (A) and power (W).

**Table S10.** Voltage, Current and Power Data from Experimental Season 1.

| **Expt** | **Trial** | **Panel Composition** | ***V*_mean_** | **sd** | ***V*_median_** | ***I*** | ***P*** | **Comments** |
| --- | --- | --- | --- | --- | --- | --- | --- | --- |
| 1 | 1 | H_2_O-H_2_O | 0.54 | 0.36 | 0.38 | 1 mA | 0.2 mW | Noisy |
|  | 2 | H_2_O-H_2_O | 0.08 | 0.03 | 0.07 | 20 nA | 1 nW |  |
|  | 3 | H_2_O-H_2_O | 0.10 | 0.07 | 0.07 | 0.1 µA | 20 nW | Small (+) excursions, noisy |
| 2 | 1 | (H_2_O + MKP)-H_2_O | 0.58 | 0.07 | 0.57 | 40 µA | 30 µW | Small (–) excursions |
| 3 | 1 | (H_2_O + rochelle salt)-H_2_O | 0.41 | 2.01 | 0.18 | 0.3 µA | 20 nW | Small (–) excursions |
|  | 2 | (H_2_O + rochelle salt)-H_2_O | 0.03 | 0.02 | 0.03 | 0.5 µA | 25 nW | Small (–) excursions |
| 4 | 1 | (H_2_O + HC_2_H_3_O_2_ + Fe)-(H_2_O + NH_3_) | 0.23 | 0.06 | 0.26 | 1 mA | 0.2 mW | Noisy |

Note: All voltages are listed in volts (V), The sd column lists standard deviation of the measured voltages. The sign of the charge is given in the comments column if there were voltage excursions present in the data.

**Table S11.** Resistance and Voltage Data from Experimental Season 1.

| **Expt** | **Trial** | ***R*_initial_** | ***V*_initial_** | ***R*_final_** | ***V*_final_** |
| --- | --- | --- | --- | --- | --- |
| 1 | 1 | 205 Ω | 0.20 V | - | 0.13 V |
|  | 2 | 3.5 MΩ | 0.068 V | - | 0.12 V |
|  | 3 | - | 0.20 V | 1.6 MΩ | 0.18 V |
| 2 | 1 | 18 kΩ | 0.71 V | - | 0.54 V |
| 3 | 1 | - | - | 279 kΩ | 0.071 V |
|  | 2 | 112 kΩ | 0.061 V | 75 kΩ | 0.042 V |
| 4 | 1 | 196 Ω | 0.21 V | 190 Ω | 0.17 V |

Note: For *Experiment 3 Trial 1*, initial data were lost. Initial R values were in the kΩ range.

Experiments 5-6 consisted of two trial panels each with 1 kΩ resistors attached in parallel. Table S12 summarizes the voltage, current, power and energy data gathered for both trials from both experiments, and presents data from each trial when the panels were covered and uncovered separately, and then for each trial in total. For Experiment 5 (kaolinite+sucrose)-acetic acid-limestone suspension panels, median power was 0.02 μW for Trial 1 and 0.001 μW for Trial 2. Over the course of about 15 hours until a computer fault stopped the data logging process, The E5T1 panel produced 0.0003 mWh of electricity. Over the course of about 40 hours, and the E5T2 panel produced 0.0004 mWh of electricity. Panel energy was on the order of 250 to 400 nWh of electricity per day at 0.001 to 0.01 V.

For Experiment 6 (kaolinite+NaCl)-HCl-MKP panels, median power was 0.1 mW for Trials 1 and 2. Over the course of 9 days, the E6T1 panel produced 20 mWh of electricity. Much of the E6T2 data were lost due to two computer faults, but for the 4.5 days of recorded operation it produced 10 mWh of electricity. Roughly, each panel produced 2 mWh of electricity per day at 0.3 V during Experiment 6.

**Table S12.** Voltage, Current and Power Data from Experiments 5 and 6 with 1 kΩ load.

| **Expt** | **Trial** | ***V*_mean_** | **sd** | ***V*_median_** | ***I*_median_** | ***P*_median_** | ***P*_mean_** | ***E*_total_** | **Comments** |
| --- | --- | --- | --- | --- | --- | --- | --- | --- | --- |
| 5 | 1 exposed (all) | 0.012 V | 0.059 V | 0.0041 V | 4.1 μA | 17 nW | 18 nW | 0.27 μWh | Small (+/–) excursions |
|  | 2 covered | 0.0024 V | 0.0028 V | 0.00125 V | 1.3 μA | 1.5 nW | 14 nW | 0.32 μWh | Noisy |
|  | 2 exposed | –0.0009 V | 0.0015 V | –0.0007 V | 0.7 μA | 0.5 nW | 3 nW | 0.05 μWh |  |
|  | 2 all | 0.0014 V | 0.0029 V | 0.0007 V | 0.7 μA | 0.5 nW | 11 nW | 0.37 μWh | Noisy |
| 6 | 1 covered | 0.214 V | 0.090 V | 0.2548 V | 0.255 mA | 64.9 μW | 53.8 μW | 5.36 mWh | Noisy |
|  | 1 exposed | 0.362 V | 0.079 V | 0.4135 V | 0.414 mA | 171 μW | 137 μW | 16.1 mWh |  |
|  | 1 all | 0.294 V | 0.112 V | 0.2610 V | 0.261 mA | 68.1 μW | 98.7 μW | 21.4 mWh | Noisy |
|  | 2 exposed | 0.315 V | 0.024 V | 0.3250 V | 0.325 mA | 106 μW | 100 μW | 2.34 mWh | Noisy |
|  | 2 covered | 0.291 V | 0.058 V | 0.2760 V | 0.276 mA | 76.2 μW | 87.9 μW | 7.22 mWh | Noisy |
|  | 2 all | 0.296 V | 0.053 V | 0.2984 V | 0.298 mA | 89.0 μW | 90.6 μW | 9.56 mWh | Noisy |

For Experiments 5-6, standardized resistors were replaced successively three and four times, respectively during each trial, to measure the performance characteristics of the panels. The power and energy output varied depending on the value of the resistor, as shown in Figures S16 and S17. Each set of measurements took approximately 30 minutes to complete for Experiment 5 and 15 minutes for Experiment 6. Table S13 shows the resistor value that produced voltage, current and power maxima during these measurements. One sees that these resistances are the same or vary by at most one order of magnitude within each panel for each of voltage, current and power except one. The 100Ω maxima for current and power for Experiment 6 Trial 1 all occurred during a transient period of direct sun when the panel was covered.

| 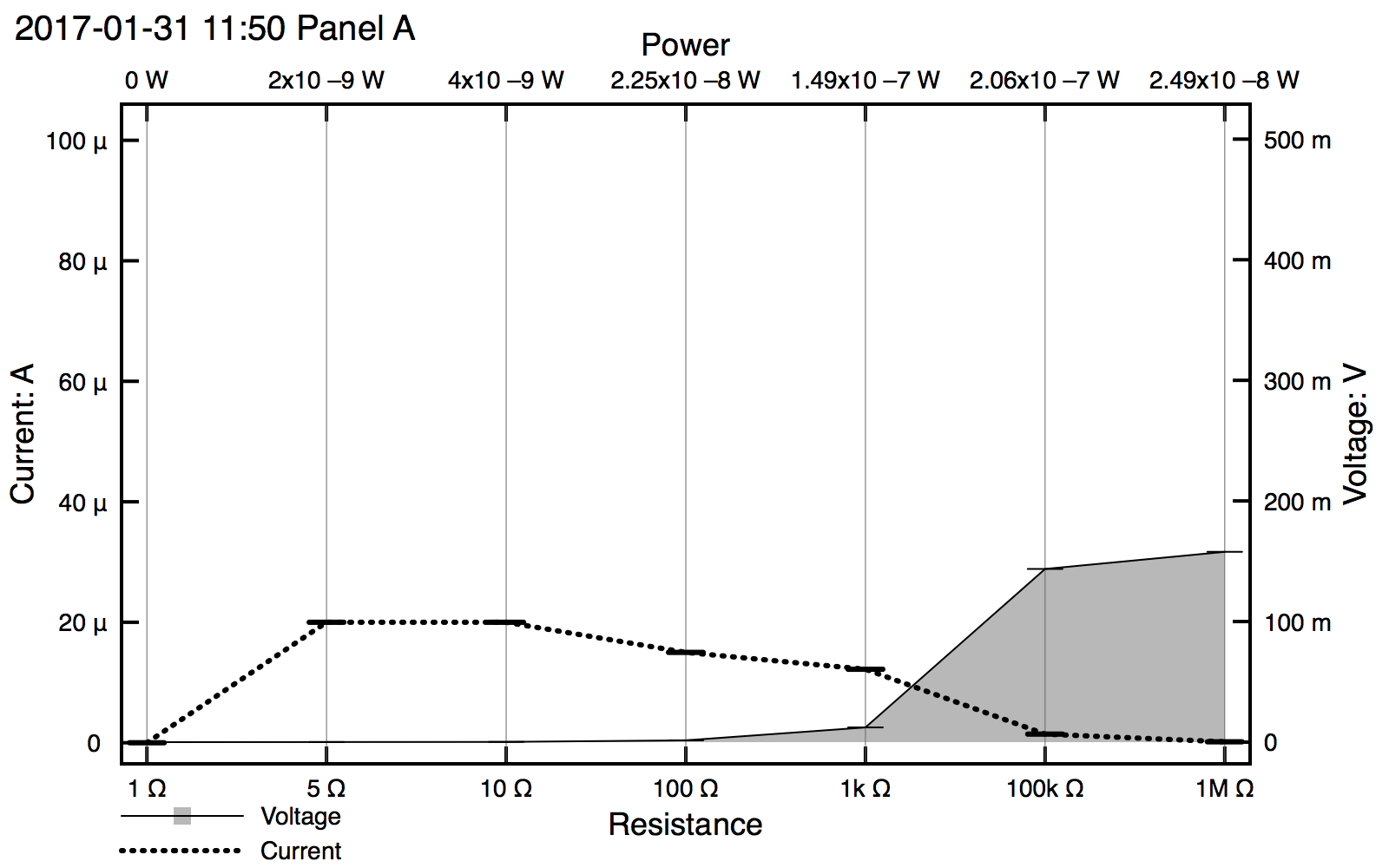 | 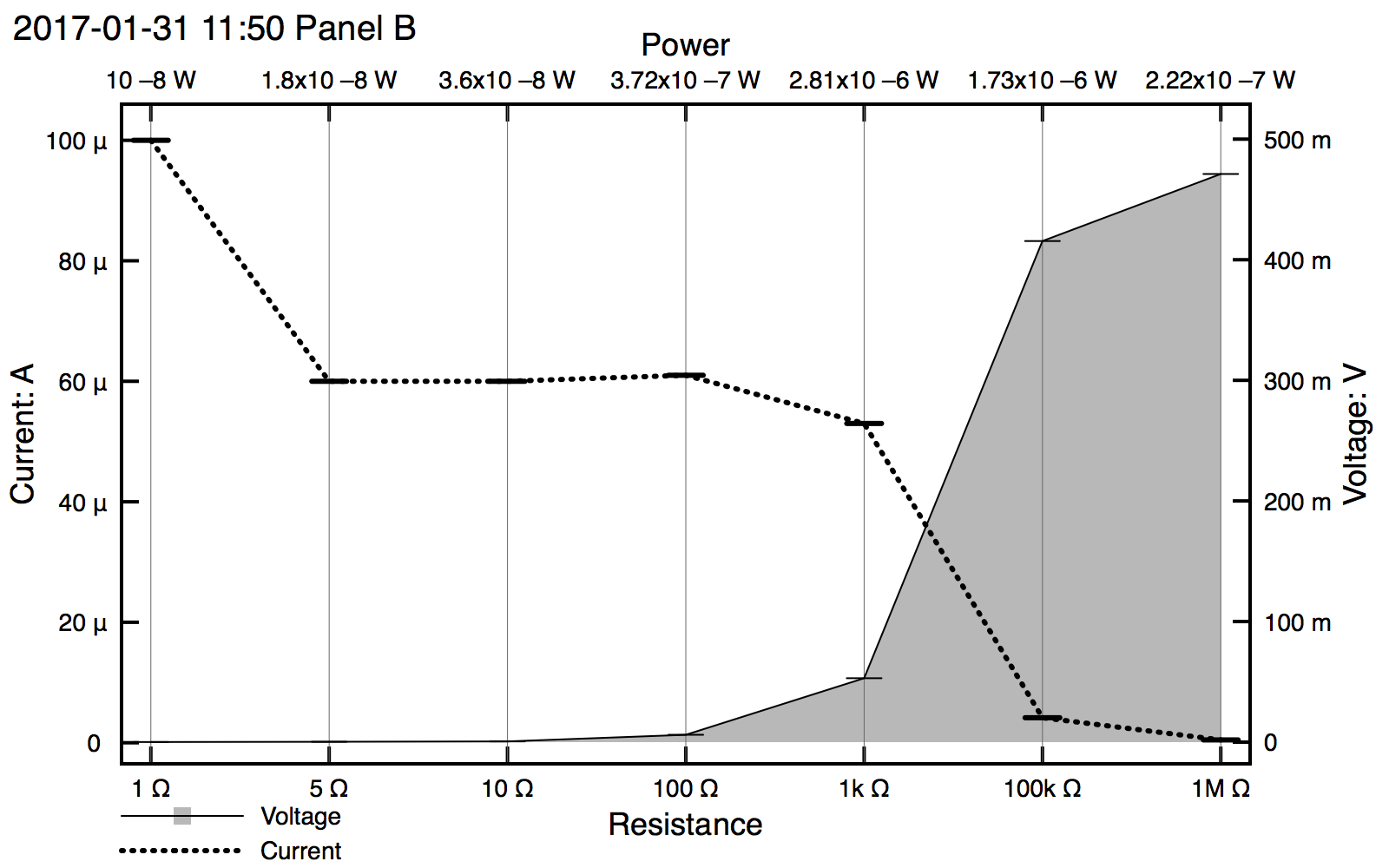 |
| --- | --- |
| 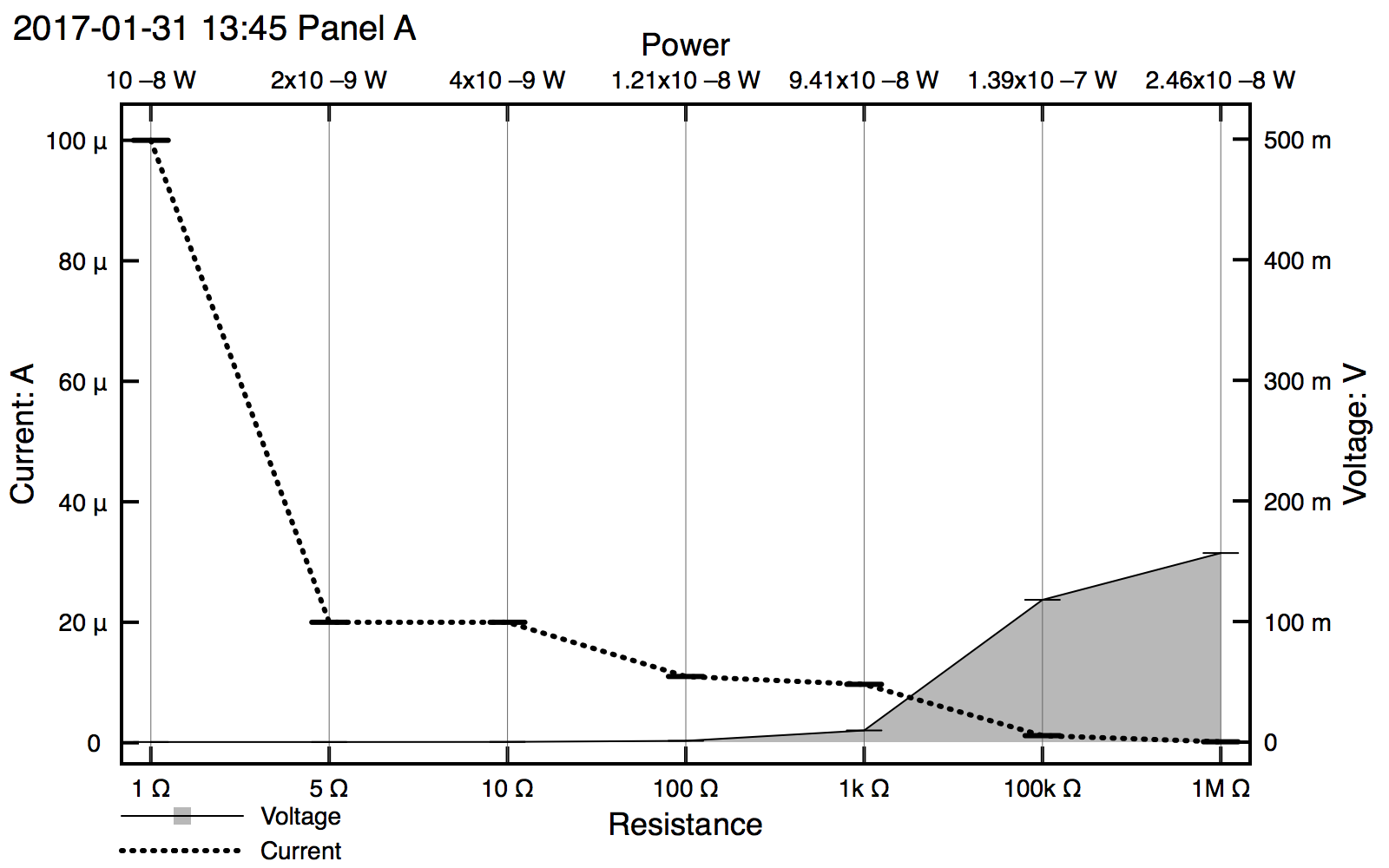 | 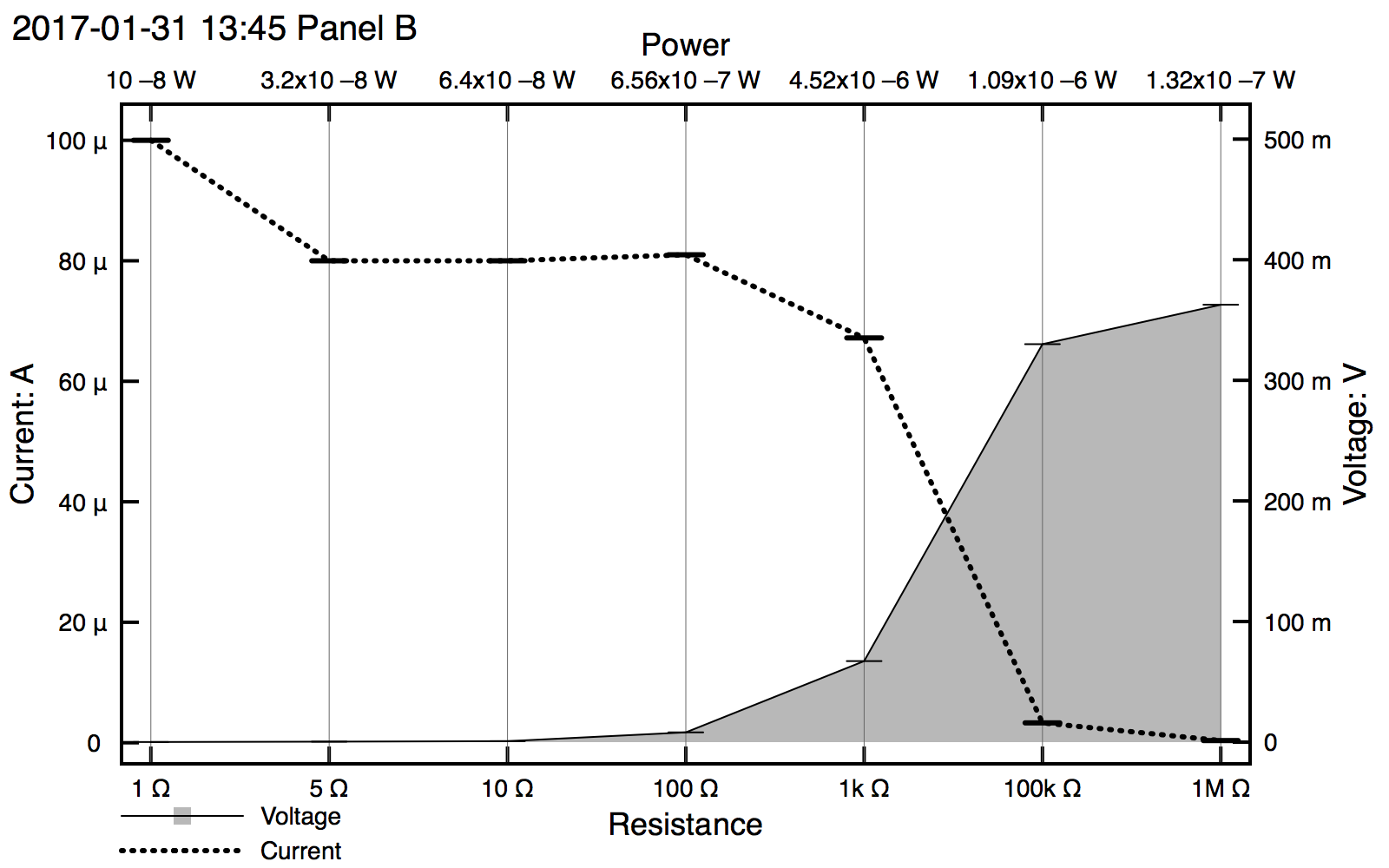 |
| 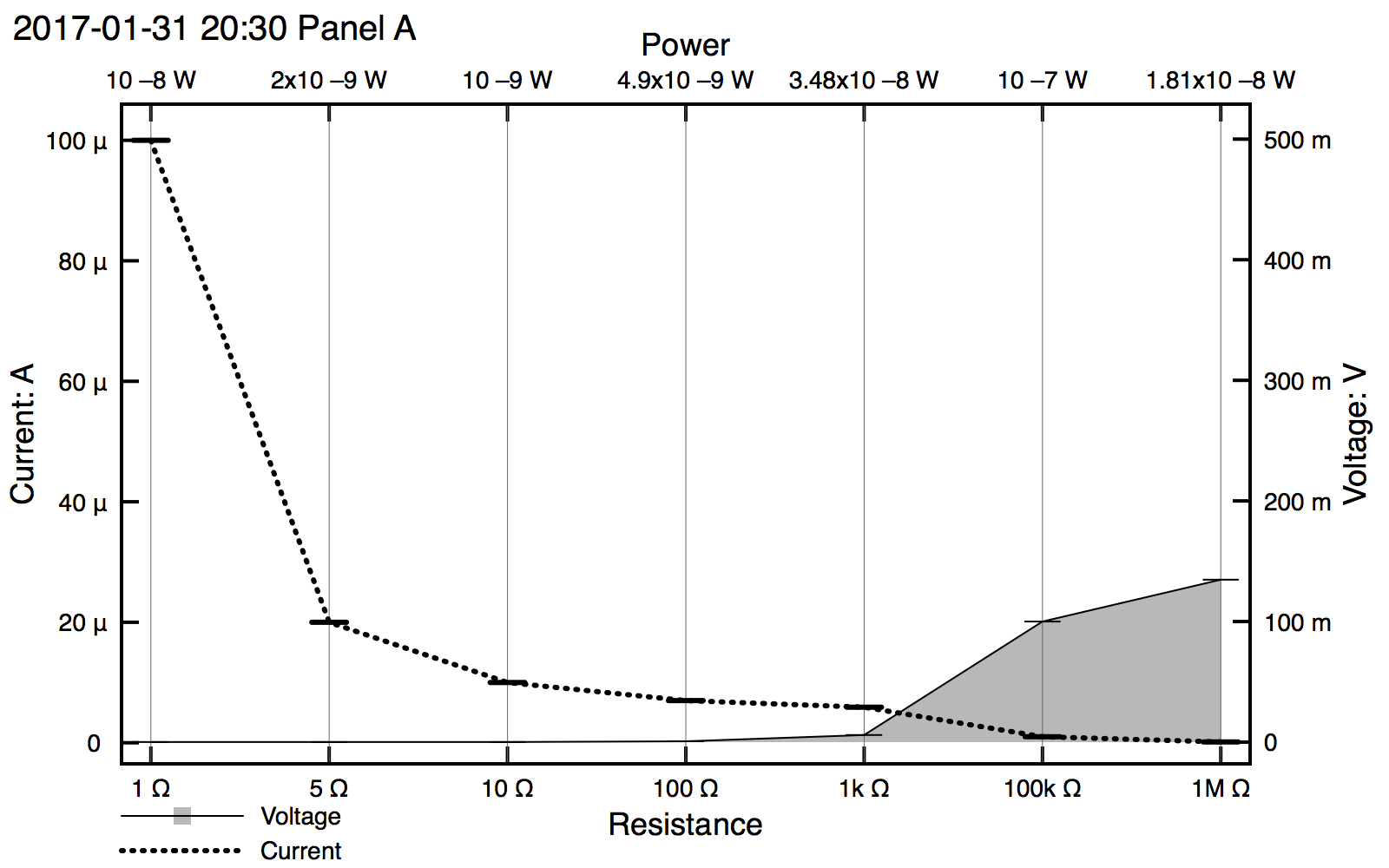 | 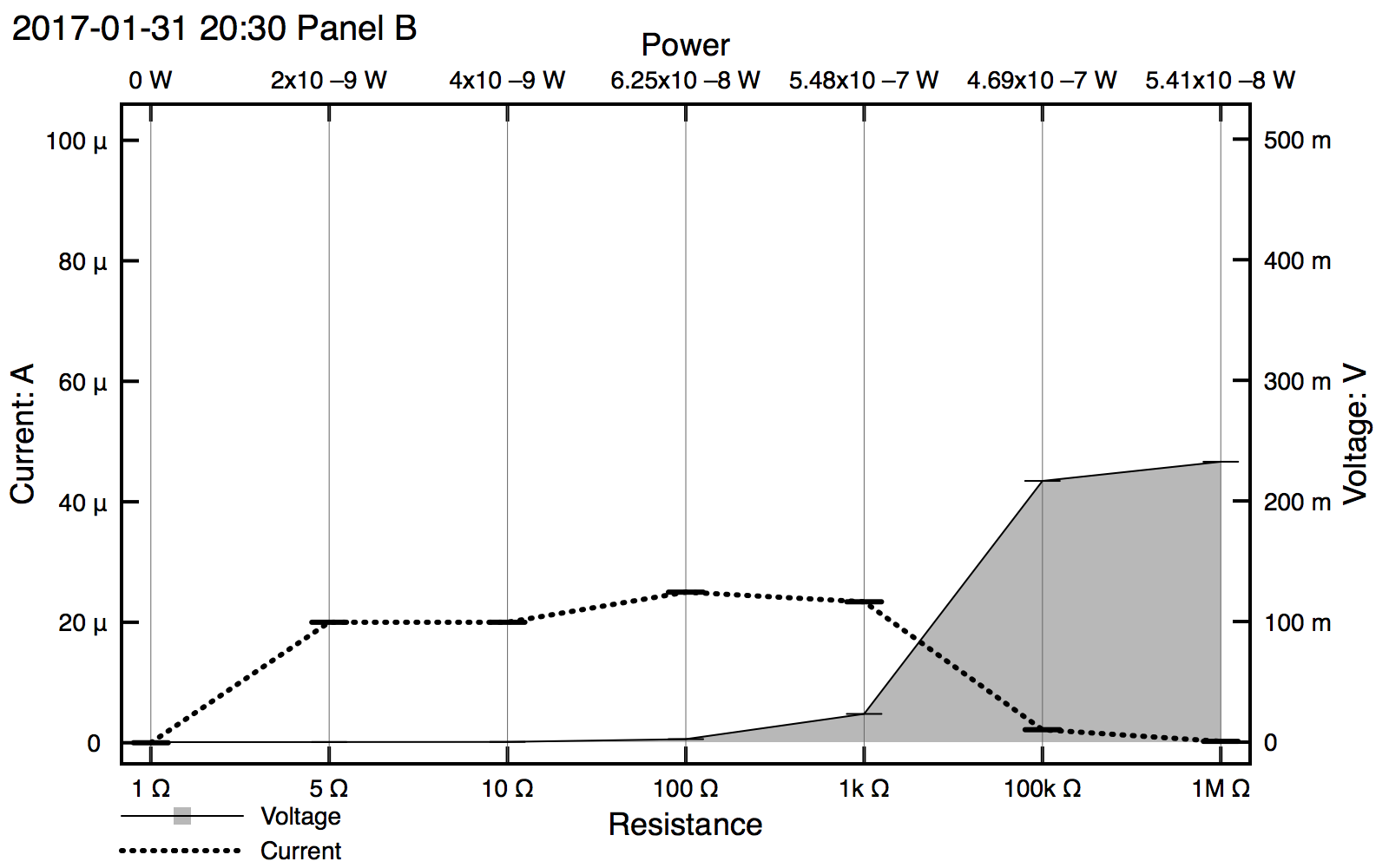 |

**Figure S16.** *Experiment 5*. Measured voltage and calculated current and power for prototype panels from (kaolinite+sucrose)-acetic acid-limestone suspension, with resistors of known value connected in parallel. Orders of magnitude for the power values are listed in-line for ease of reading but should be read as superscripts, e.g. the top left power value of the top right panel should be read as 10^–8^ W. The shaded plot on the right of each graph shows power production. Panel A refers to Trial 1; Panel B refers to Trial 2. During these measurements, Panel A was covered and Panel B was exposed. Each set of measurements took approximately 30 minutes to complete.

| 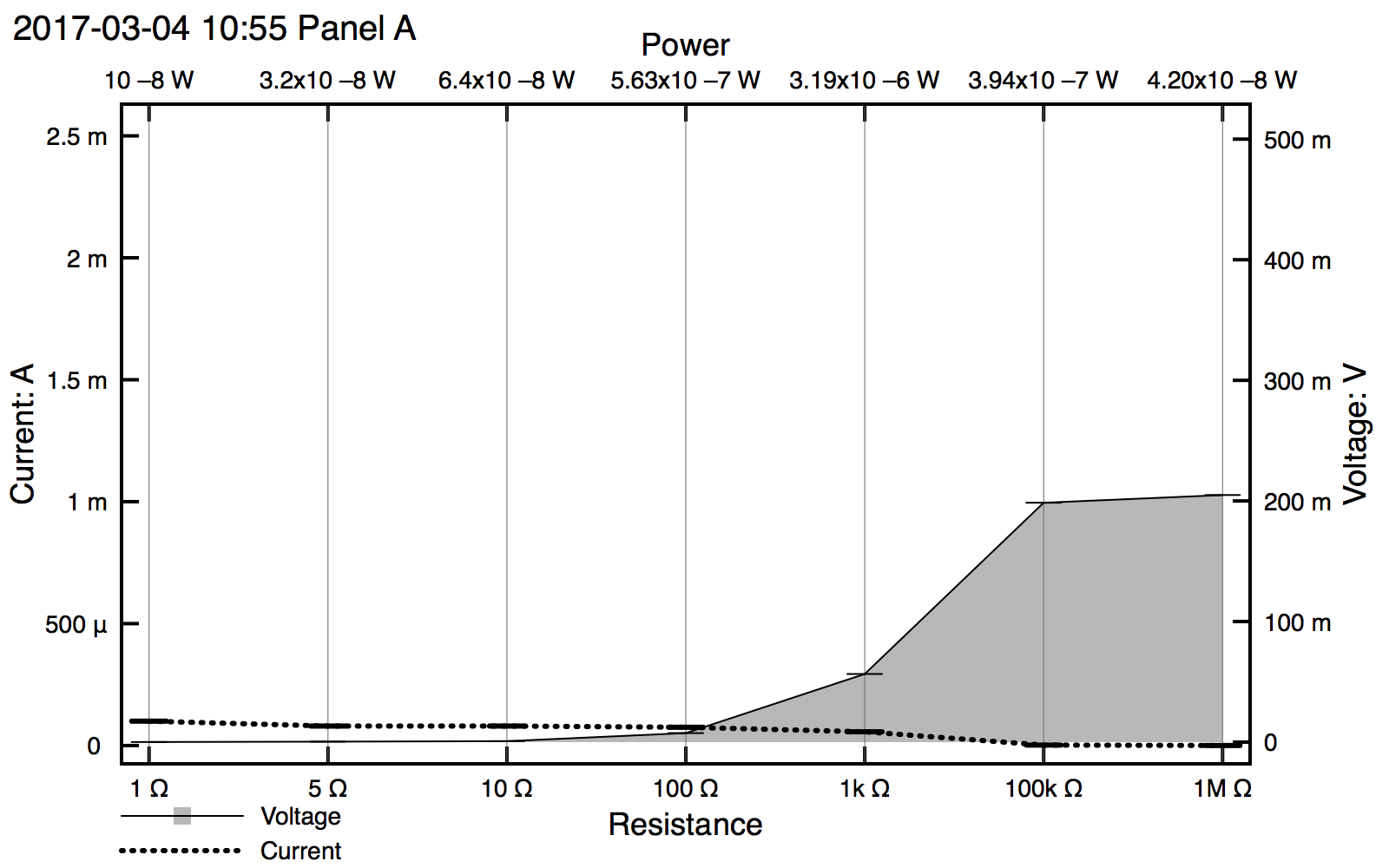 | 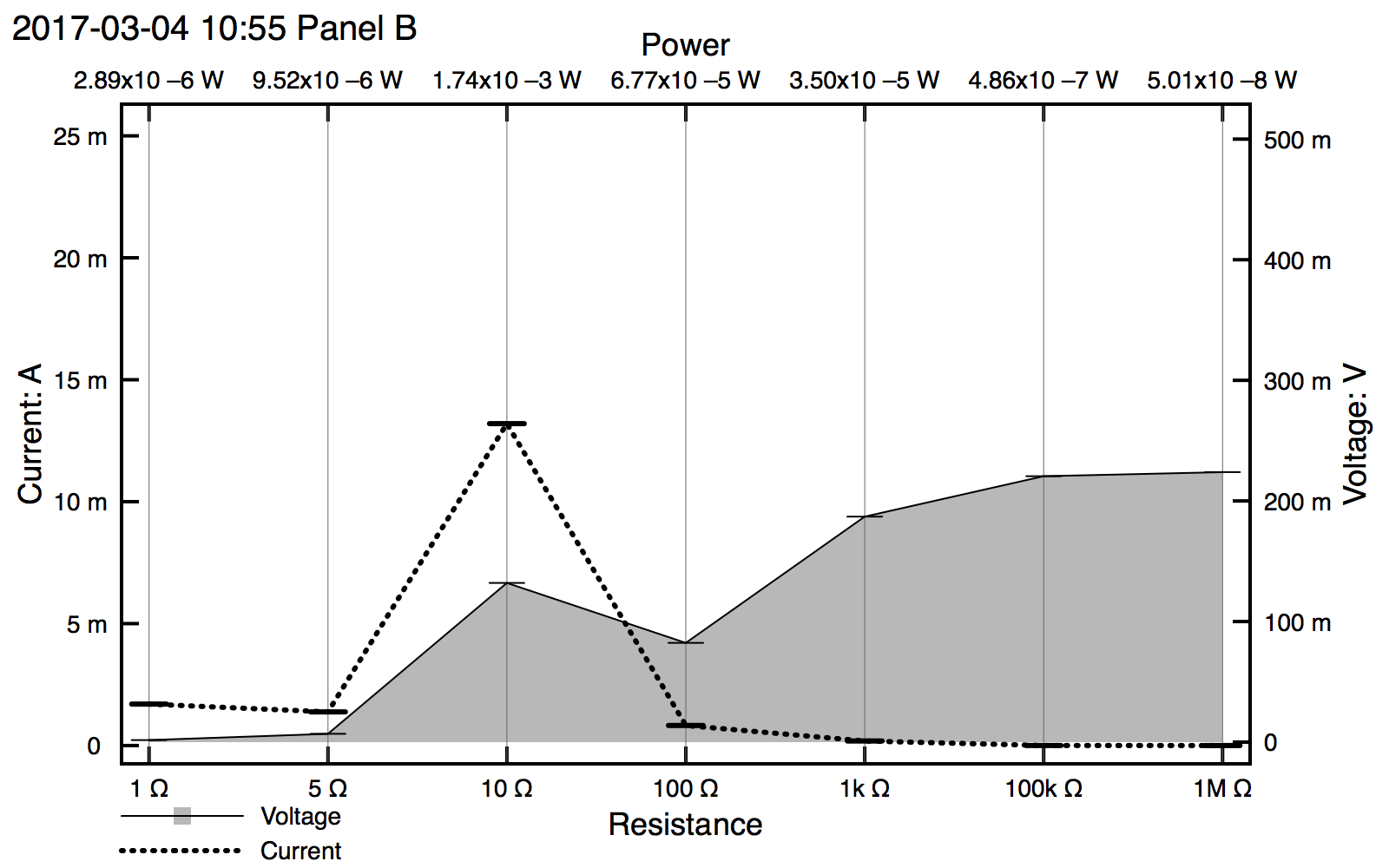 |
| --- | --- |
| 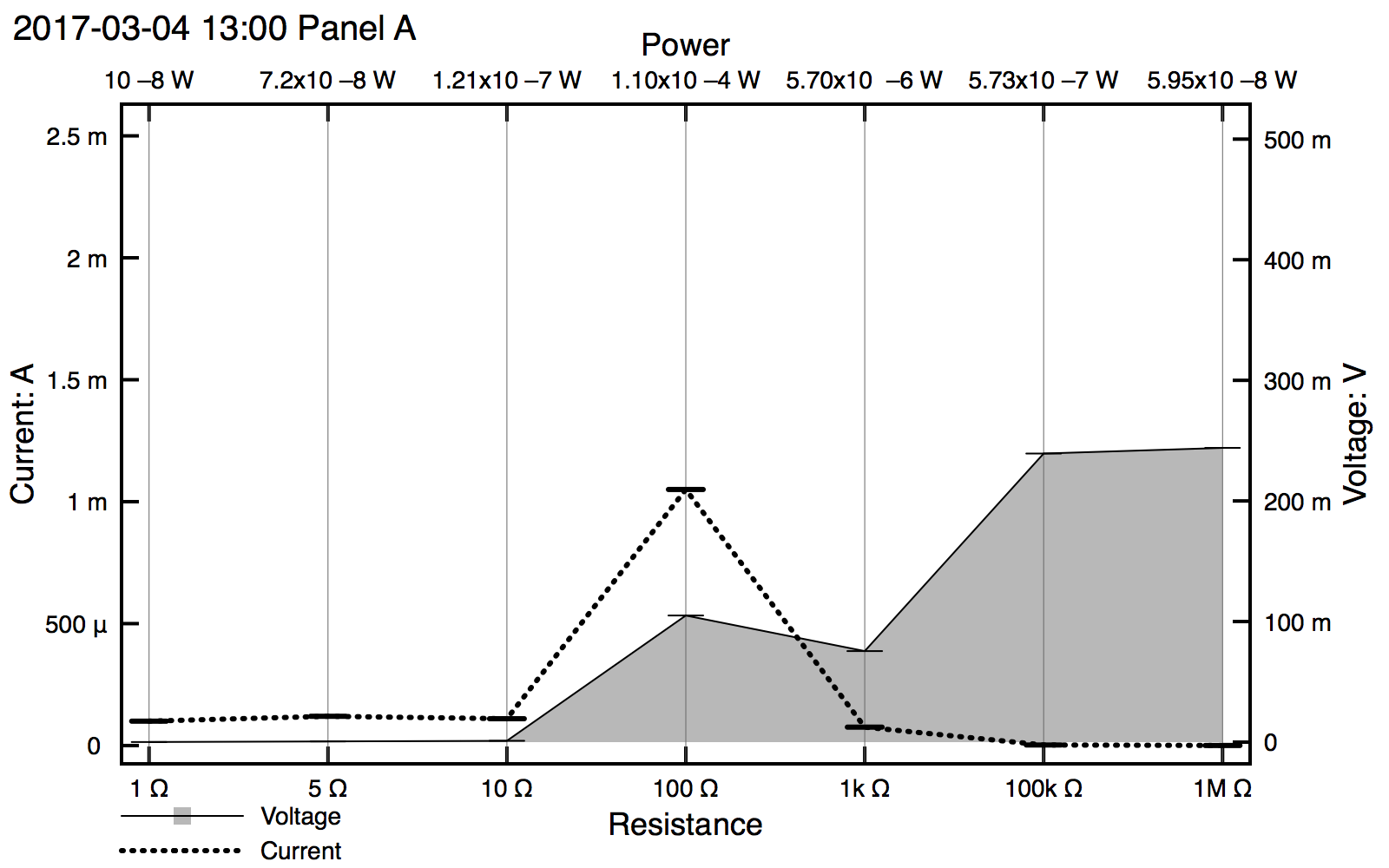 | 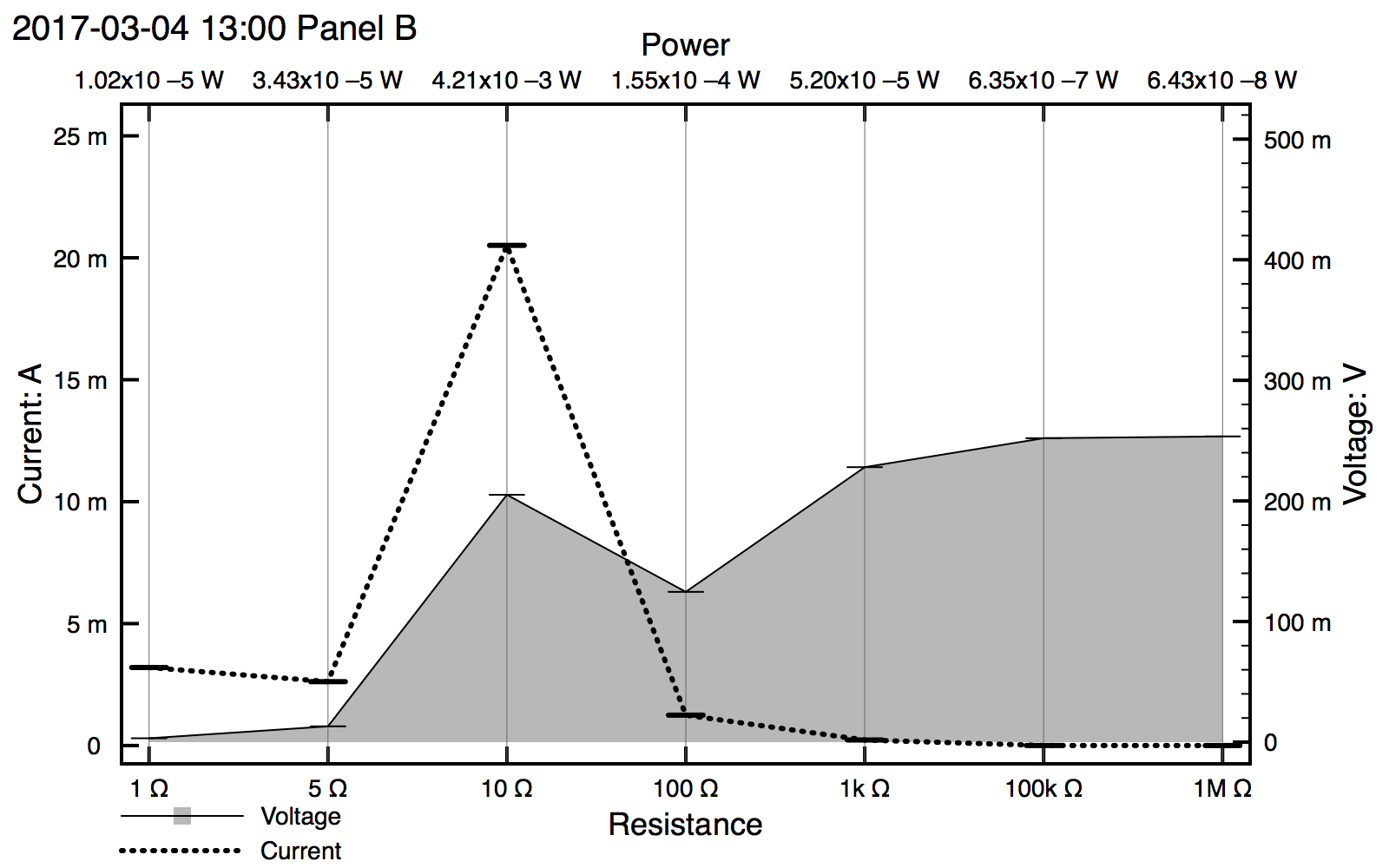 |
| 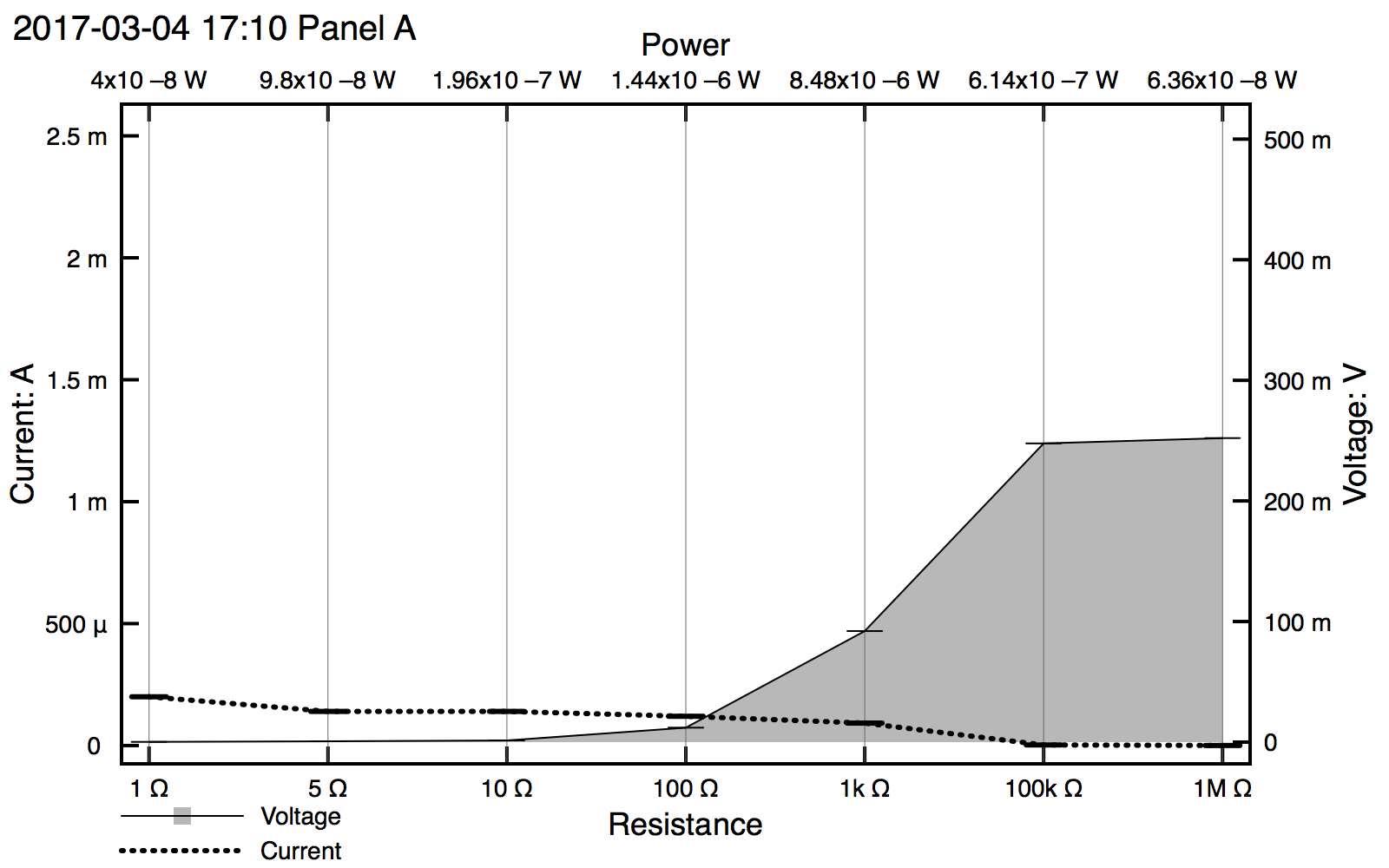 | 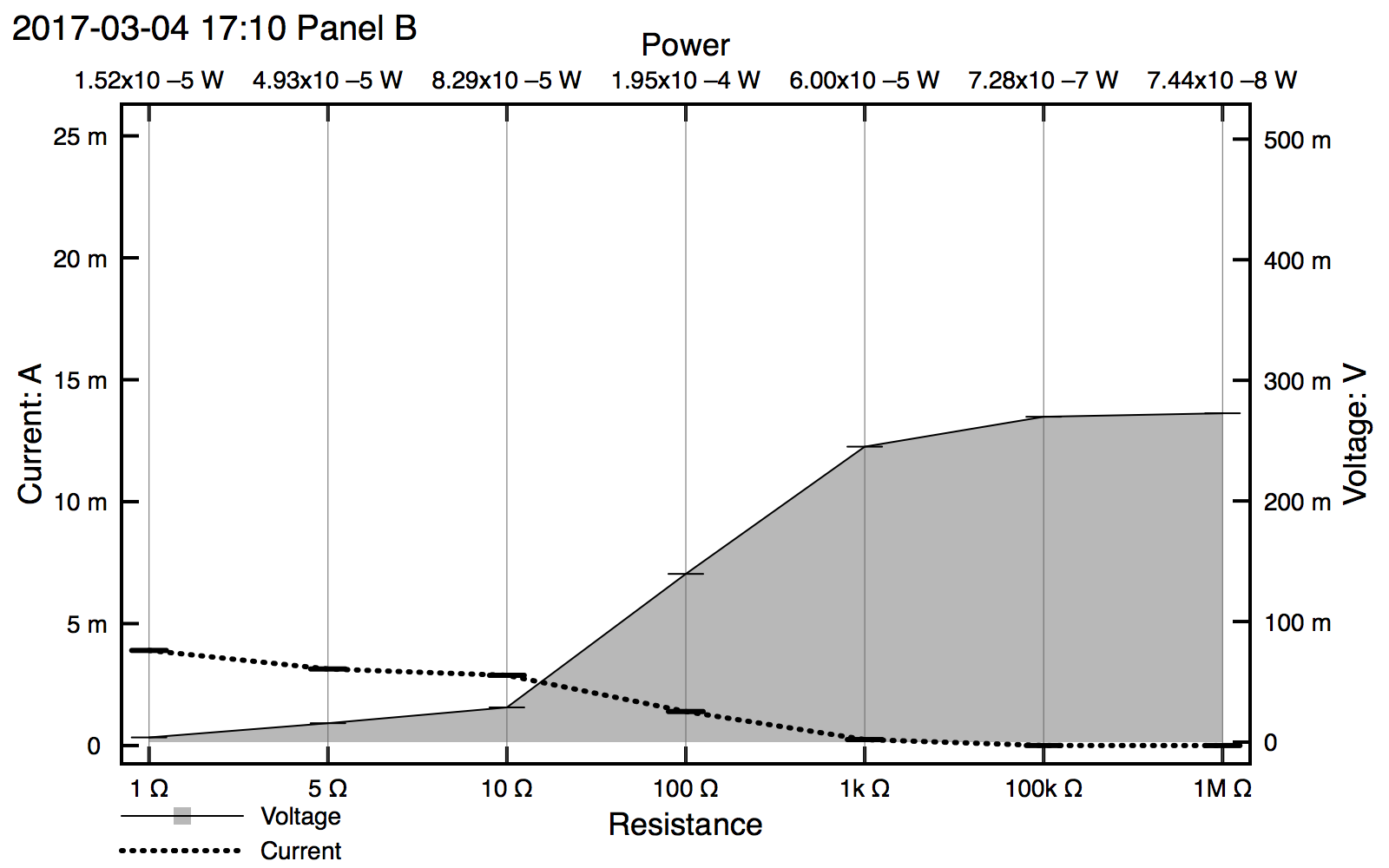 |
| 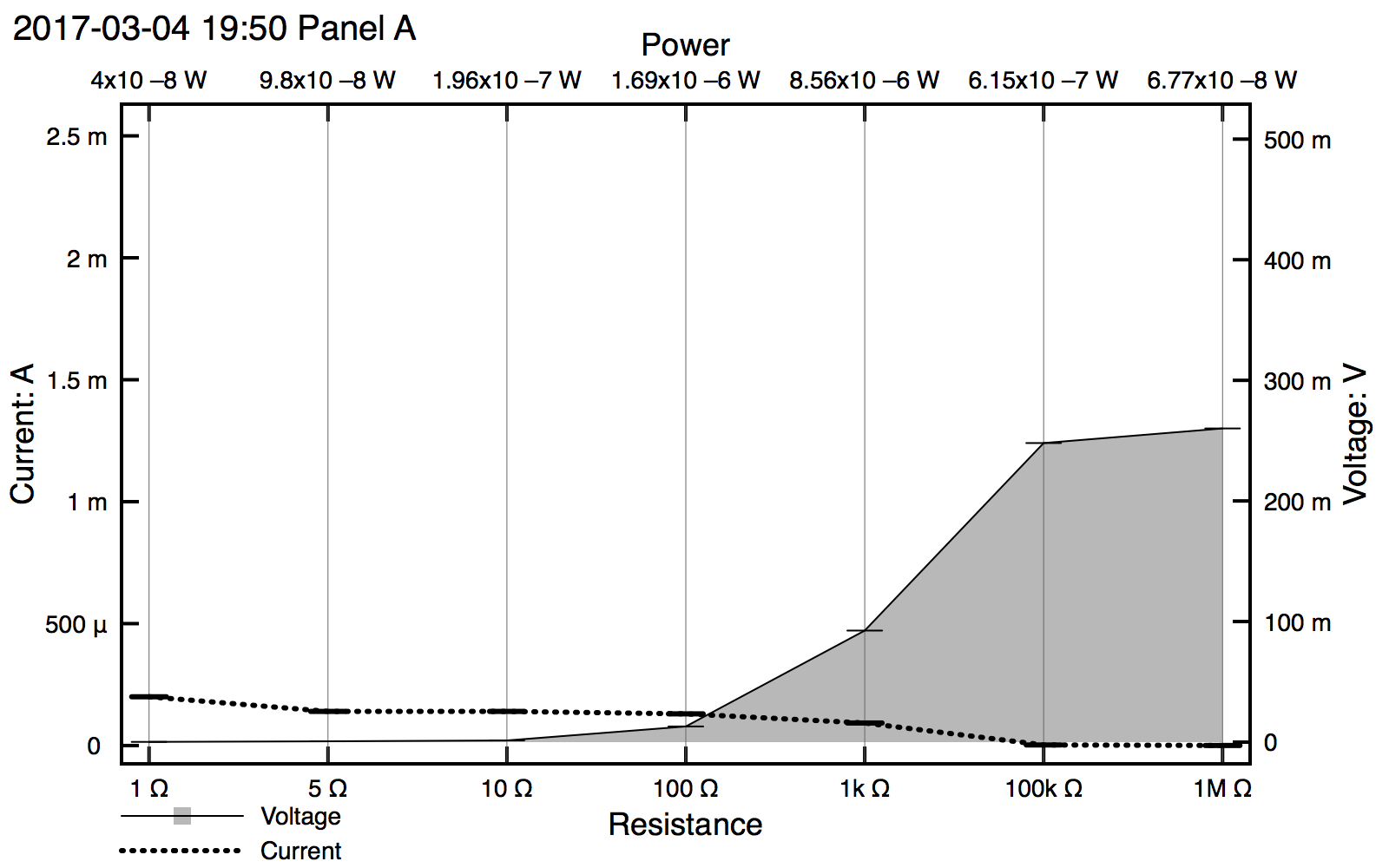 | 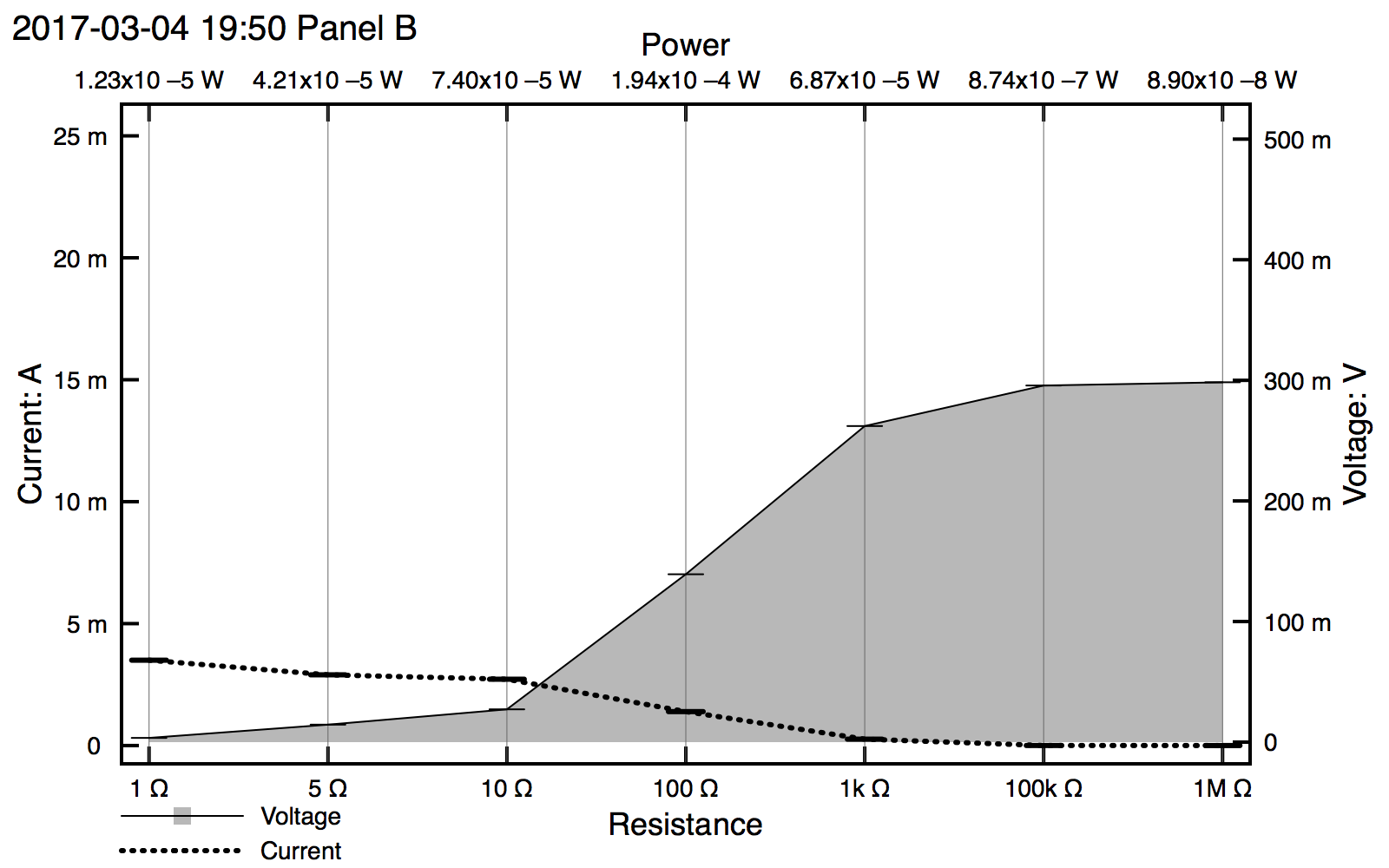 |

**Figure S17.** *Experiment 6.* Measured voltage and calculated current and power for prototype panels from (kaolinite+NaCl)-HCl-MKP, with resistors of known value connected in parallel. Orders of magnitude for the power values are listed in-line for ease of reading but should be read as superscripts, e.g. the top left power value of the top left panel should be read as 10^–8^ W. The shaded plot on the right of each graph shows power production. Panel A refers to Trial 1; Panel B refers to Trial 2. During these measurements, Panel A was covered and Panel B was exposed. Each set of measurements took approximately 15 minutes to complete. Current scale (amperes) for Panel B is 10x the scale for Panel A.

**Table S13.** Resistance Associated with Maximal Voltage, Current and Power for Experiments 5 and 6.

| **Expt** | **Value** | **Resistor t_1_** | **Resistor t_2_** | **Resistor t_3_** | **Resistor t_4_** |
| --- | --- | --- | --- | --- | --- |
| 5 | *V*_1_ max | 1 MΩ | 1 MΩ | 1 MΩ |  |
|  | *V*_2_ max | 1 MΩ | 1 MΩ | 1 MΩ |  |
|  | *I*_1_ max | 5 Ω | 1 Ω | 1 Ω |  |
|  | *I*_2_ max | 1 Ω | 1 Ω | 5 Ω |  |
|  | *P*_1_ max | 100 kΩ | 100 kΩ | 100 kΩ |  |
|  | *P*_2_ max | 1 kΩ | 1 kΩ | 1 kΩ |  |
| 6 | *V*_1_ max | 1 MΩ | 1 MΩ | 1 MΩ | 1 MΩ |
|  | *V*_2_ max | 1 MΩ | 1 MΩ | 1 MΩ | 1 MΩ |
|  | *I*_1_ max | 1 Ω | 100 Ω | 1 Ω | 1 Ω |
|  | *I*_2_ max | 10 Ω | 10 Ω | 1 Ω | 1 Ω |
|  | *P*_1_ max | 1 kΩ | 100 Ω | 1 kΩ | 1 kΩ |
|  | *P*_2_ max | 10 Ω | 10 Ω | 100 Ω | 100 Ω |

Note: Subscripts for voltage (*V*), current (*I*) and power (*P*) refer to different trials, and these ran concurrently during each experiment. Subscripts for the time (t) refer to each of three or four times that different values of resistors were successively replaced in each circuit and measurements taken. Resistance values were taken three times for Experiment 5 and four times for Experiment 6.

Figures S18 and S19 show features of the voltage data for Experiments 1-4 and 5-6, respectively. The association of voltage with environmental features is not consistent. For example, the noisy peak and trough dominating the right half in E1T1 occurred during a period of falling temperatures after a very brief positive temperature excursion at night. In contrast the peak on the left side in E1T2 occurred immediately antecedent to sunset.

| 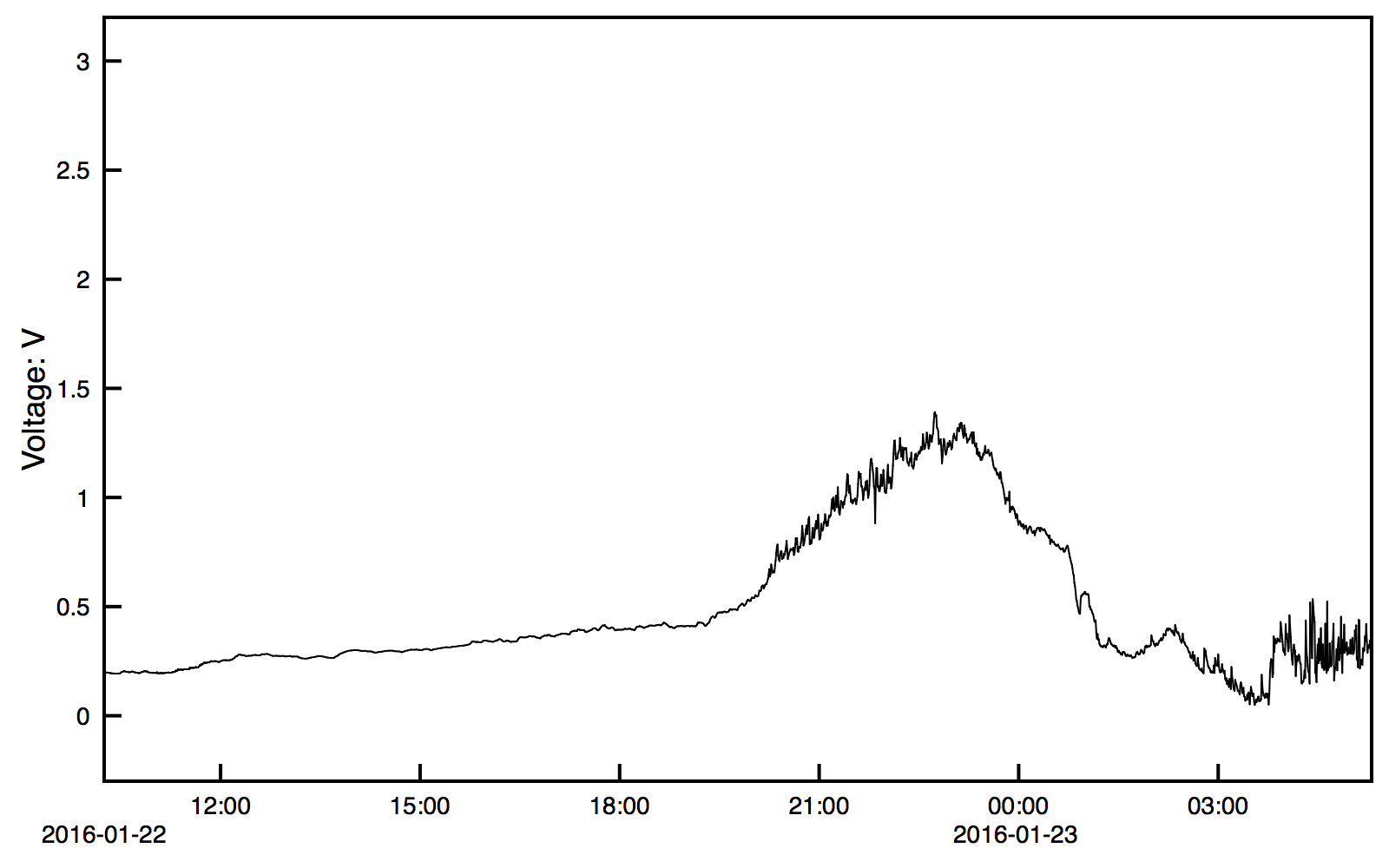 | 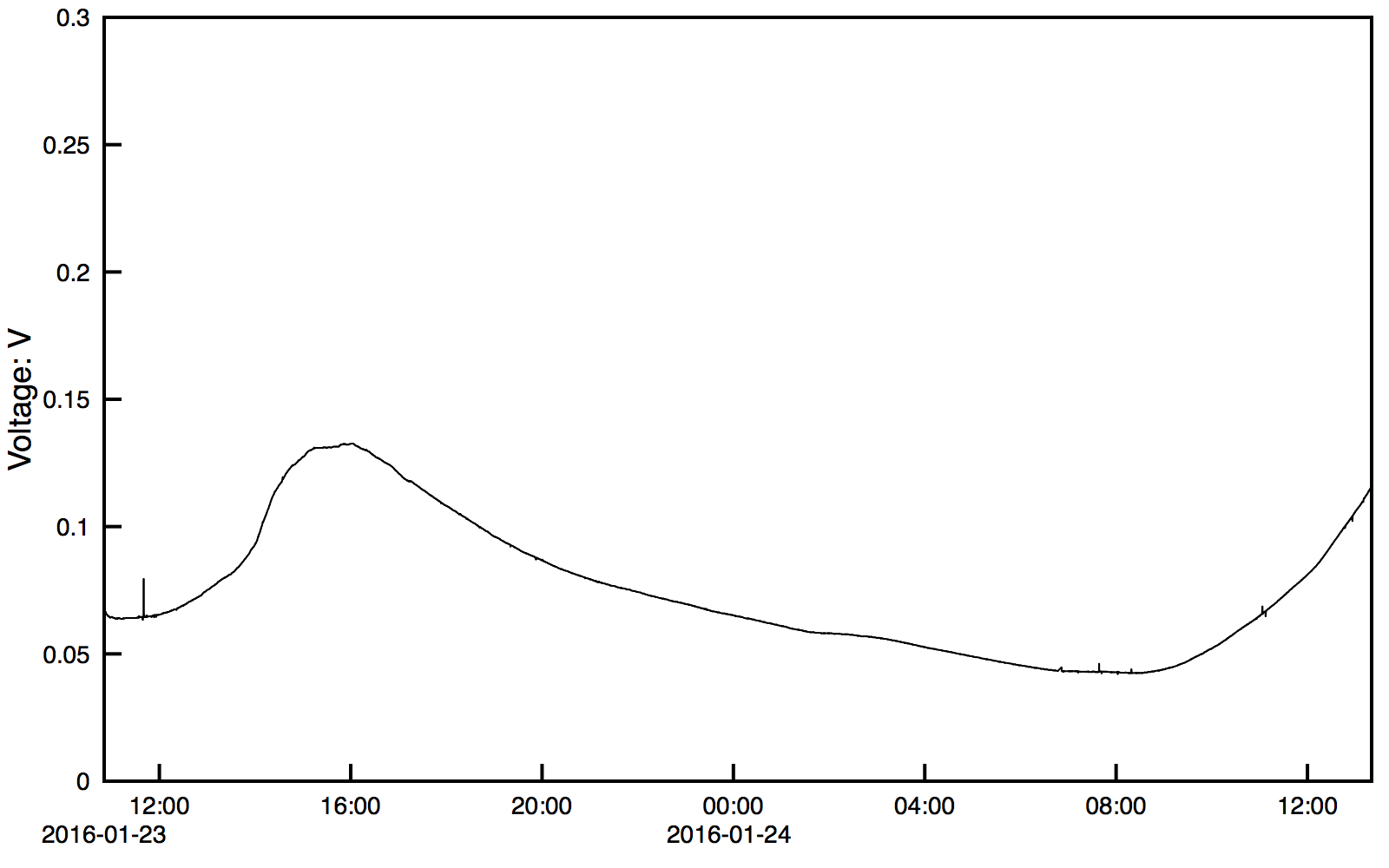 |
| --- | --- |
| 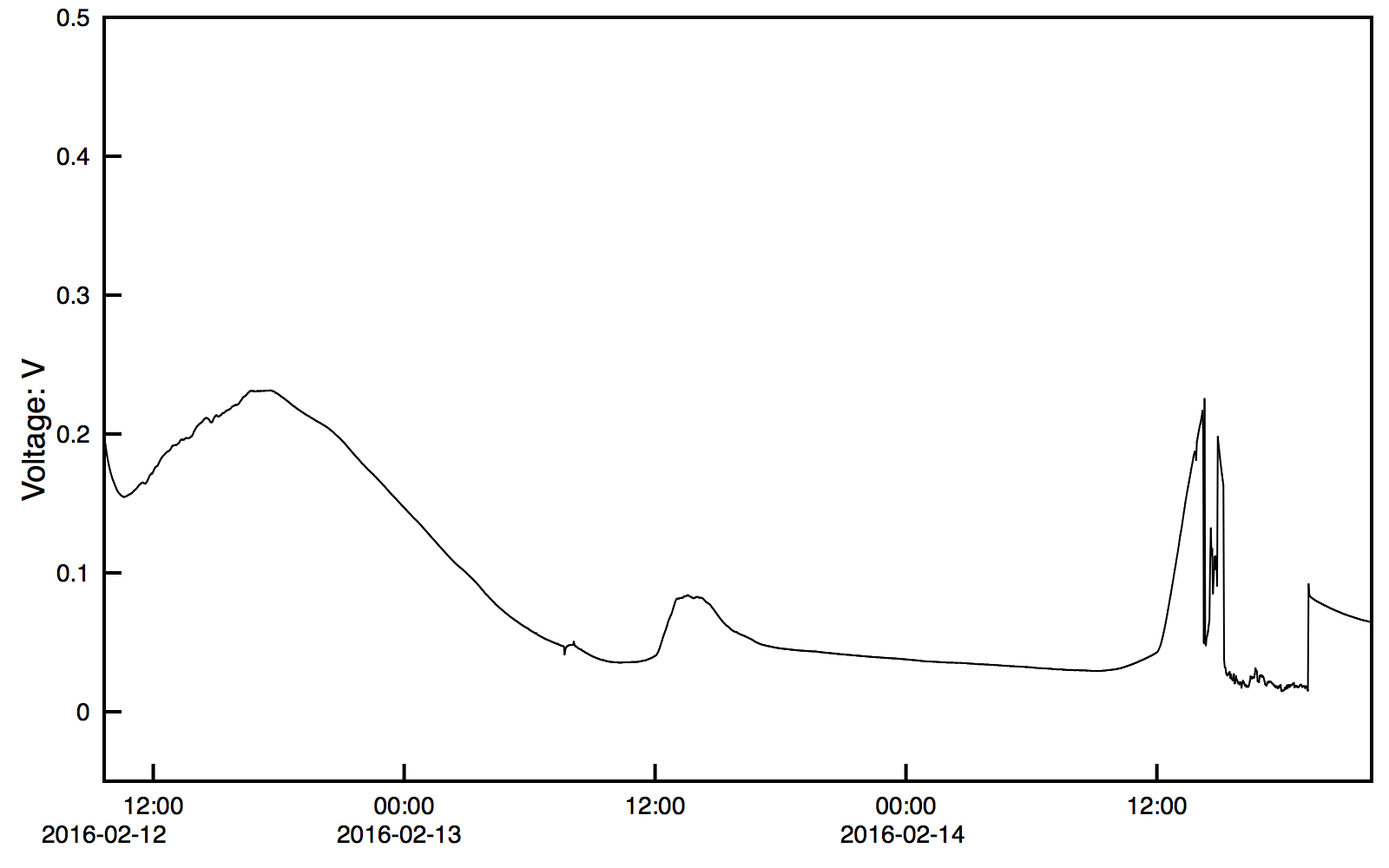 | 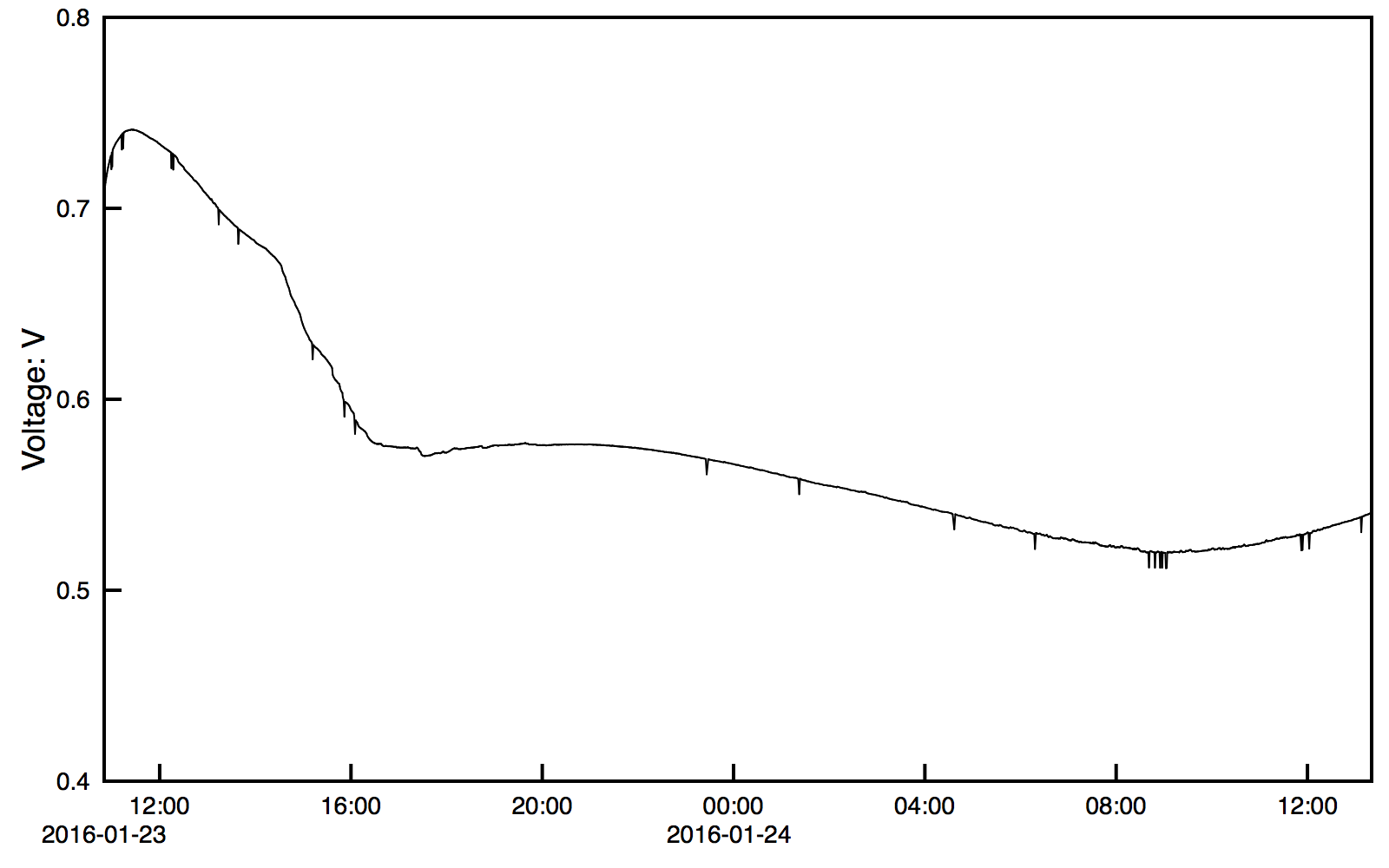 |
| 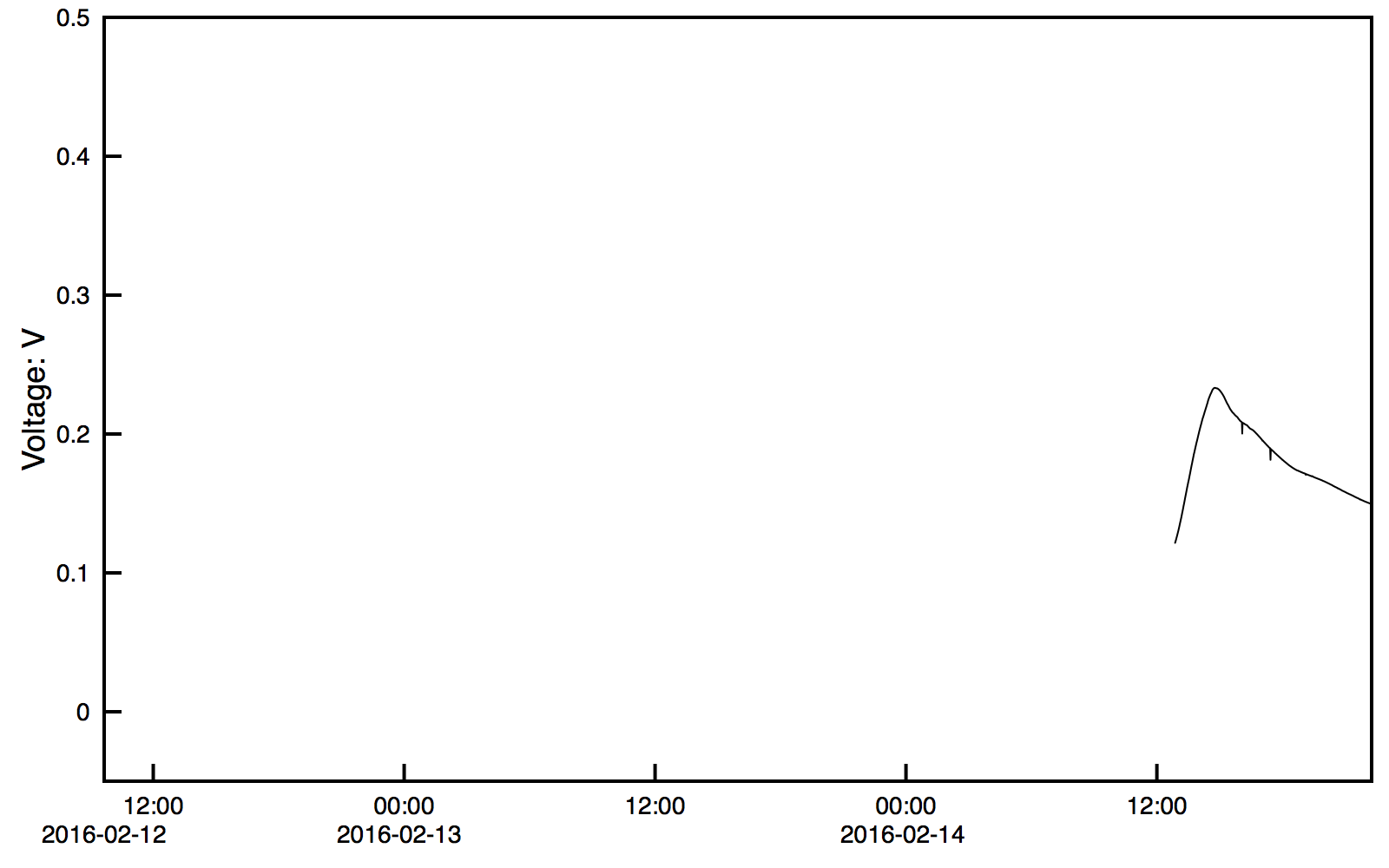 | 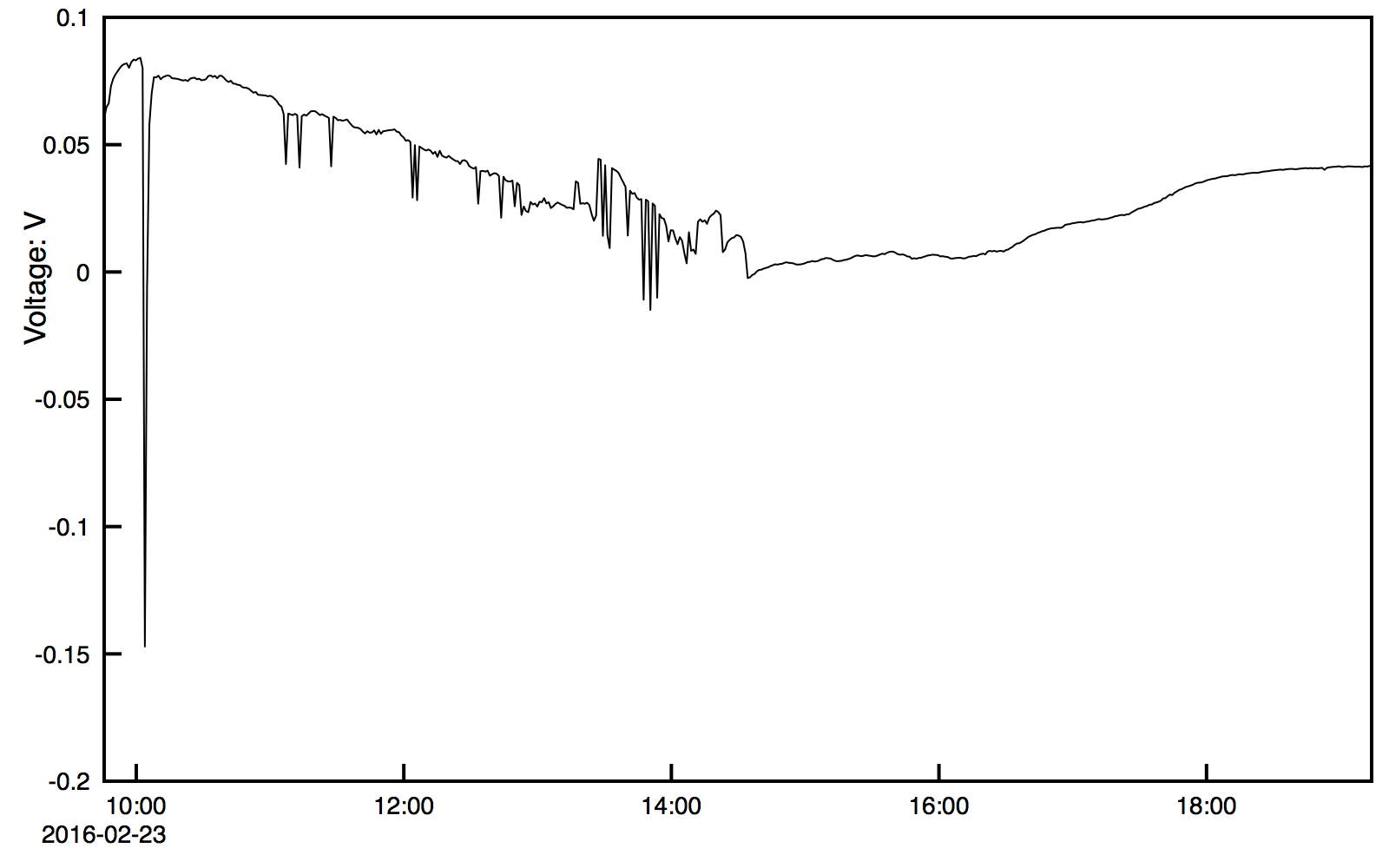 |
| 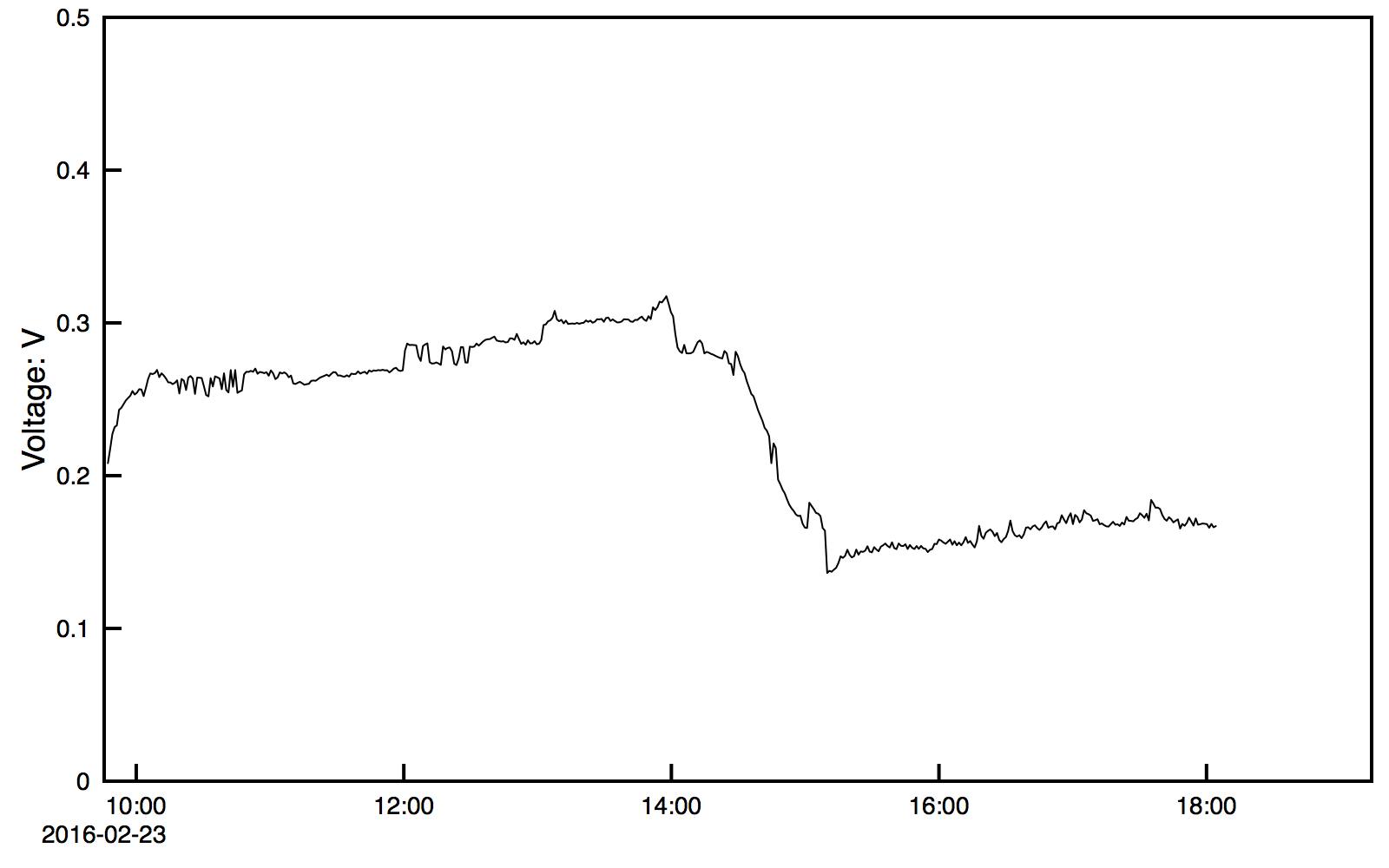 |  |

**Figure S18.** 1-minute voltage data showing observed plot features. Two-layer ice panels. (top row) E1T1, E1T2. (second row) E1T3, E2T1. (third row) E3T1, E3T2. (bottom row) E4T1. Missing traces are from loss of data due to computer faults. Abbreviations: E = Experiment. T = Trial. *Experiment 1*: water ice / water ice. *Experiment 2*: monopotassium phosphate + water ice / water ice. *Experiment 3*: rochelle salt + water ice / water ice. *Experiment 4*: acetic acid 5% + iron filings + water ice / ammonia 2% + water ice.

| 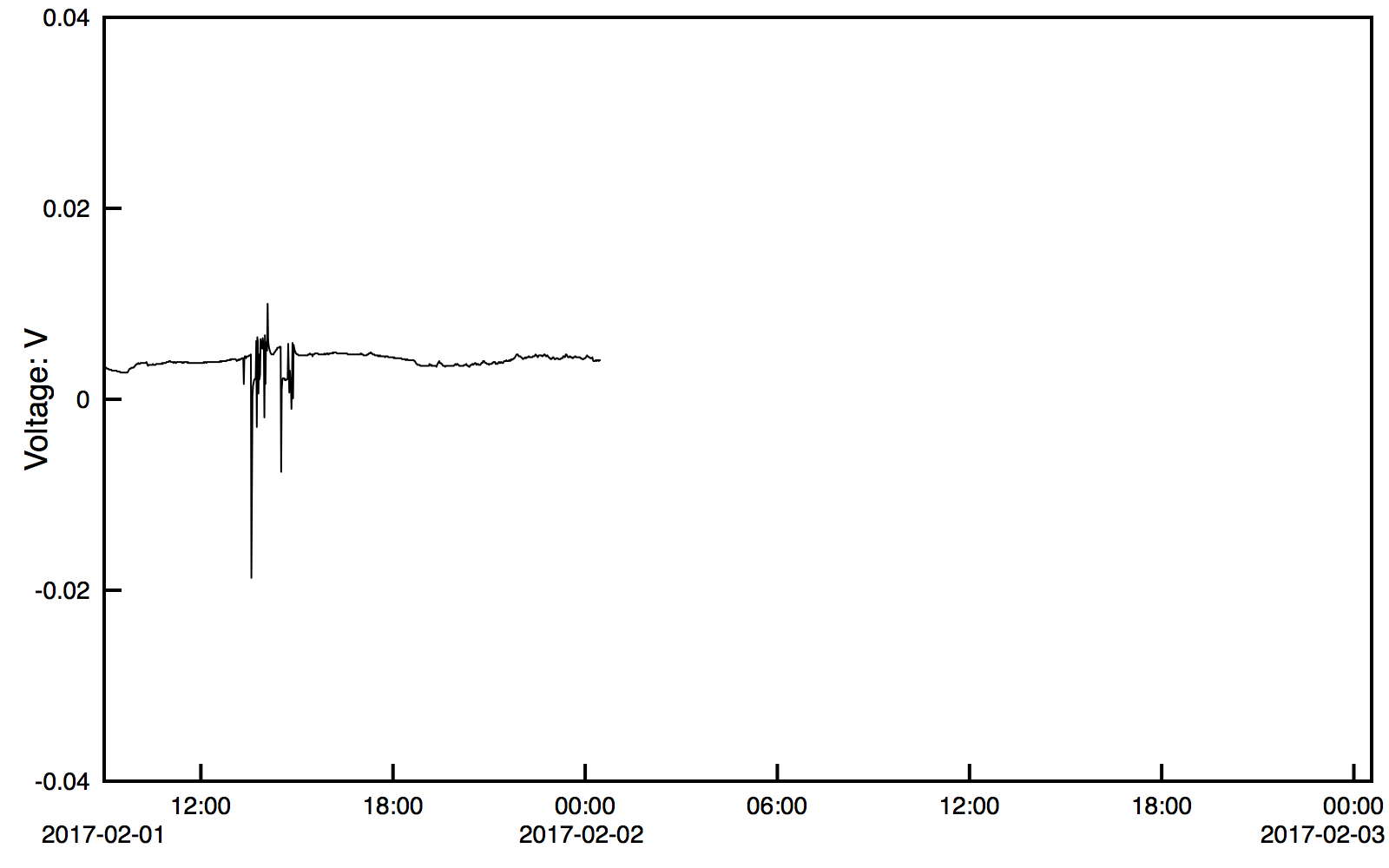 | 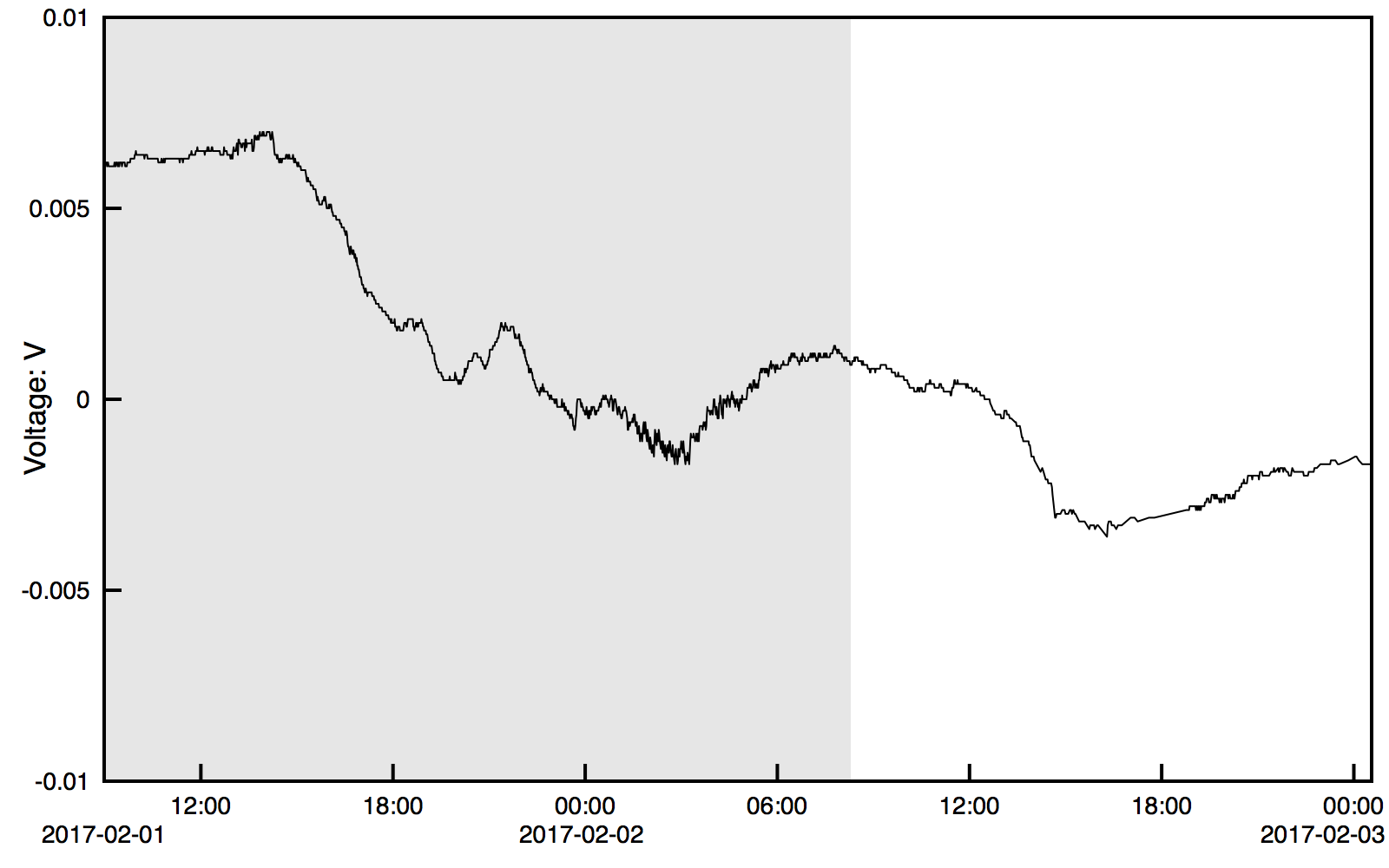 |
| --- | --- |
| 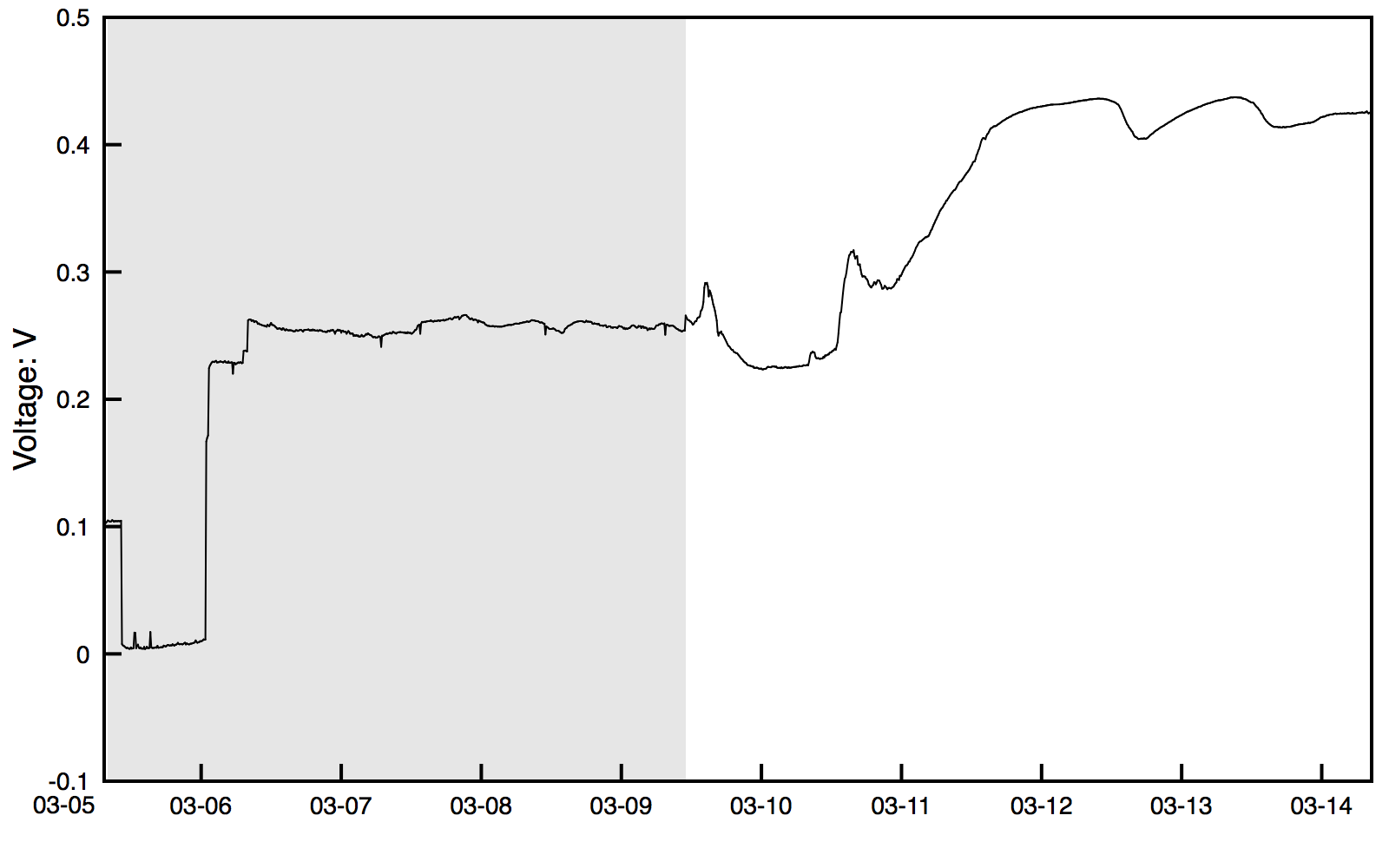 | 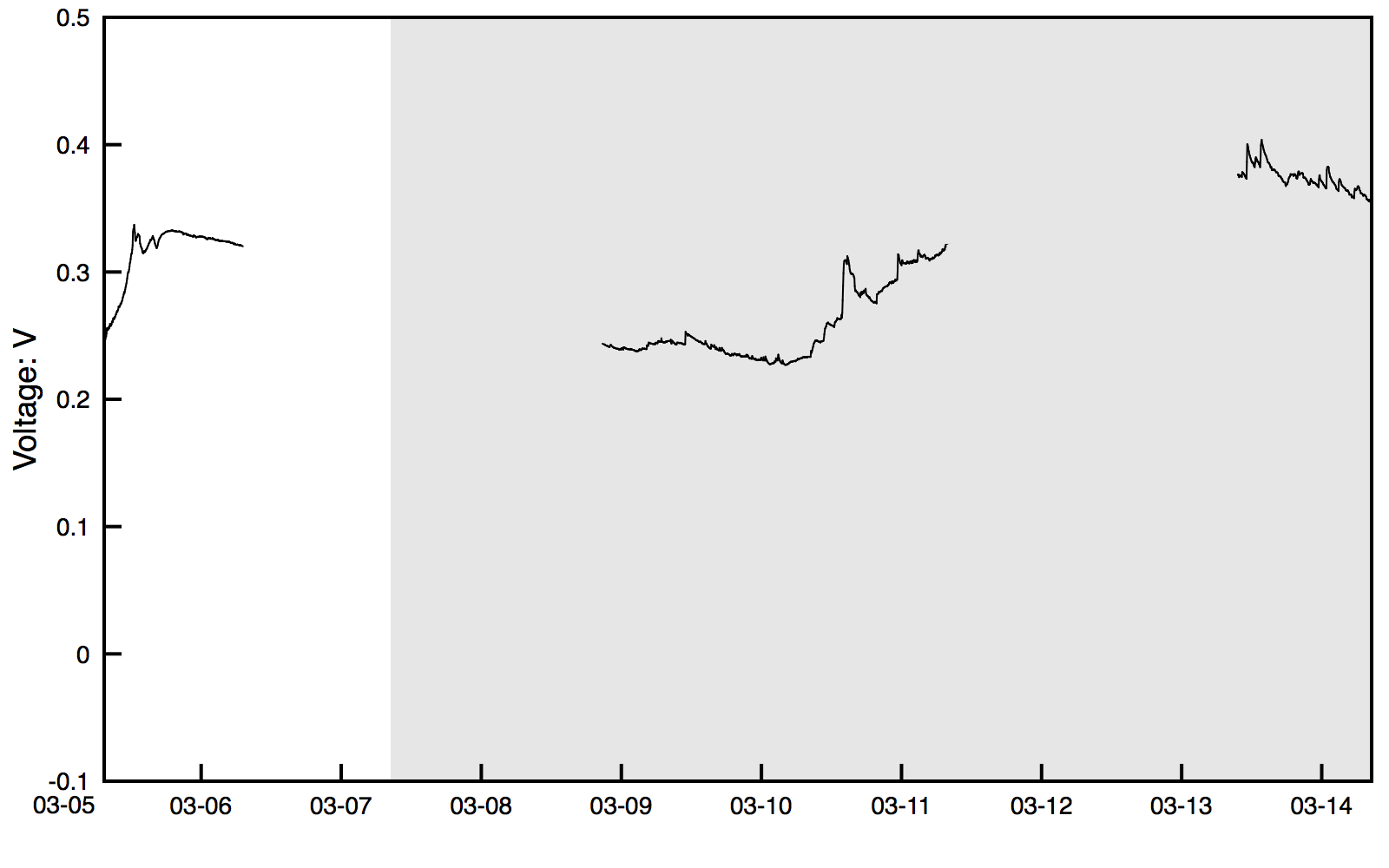 |

**Figure S19.** 1-minute voltage data showing coincident features. Two-layer ice panels with additional unfrozen middle. (top row) E5T1, E5T2. (bottom row) E6T1, E6T2. Grey indicates period when panel was covered. Missing traces are from loss of data due to computer faults. Abbreviations: E = Experiment. T = Trial. *Experiment 5*: kaolinite + sucrose + water ice / acetic acid 5% / limestone + water ice. *Experiment 6*: kaolinite + sodium chloride + water ice / hydrochloric acid 2% / monopotassium phosphate + water ice.

To make sense of these results with more clarity, let's look at what can be learned from all the trials, from Experiments 1-6. Some results are notable. The correlation between temperature and voltage during the experimental trials was striking in some cases. See, for example, the best fits, namely Year 1 Run 2 (E1T2, E2), and Year 1 Run 4 (E1T3, E3T1), shown here in Figure S20. Peaks and troughs in temperature and voltage are visually correlated.

This is not the only correspondence of note in Figure S20. The Year 1 Run 4 (E1T3) water-water panel (middle right) is a case: The temperature correlation exists until about 12:00 on Feb. 14, and then there are excursions and rapid changes in voltage over about six hours before stabilizing into a quiet concave curve, afterwards again matching the temperature. The start of the noisy interval corresponds with a peak in temperature.

The Year 1 Trial 1 (E1T1) water-water panel in Figure S21 (left top) is another case: It shows no correlation between the voltage and temperature, but a local positive temperature excursion at 20:00 on Jan. 22 is associated with a noisy period. The Year 2 Run 1 (E5T1) (kaolinite+sucrose)-acetic acid-limestone suspension (Panel A) in Figure S21 (right top) is another case. It shows the onset of a noisy interval coincident with peak temperature. These onset periods of voltaic noise in these cases correspond to periods of peak temperature, and are consistent with a crystallographic reorganization at that time based on a thermodynamic or pyroelectric mechanism.

| Year 1 Run 2  Top: E2 MKP-water  Middle: E1T2 Water-water  Bottom: Temperature | Year 1 Run 4  Top: E3T1 Rochelle salt-water  Middle: E1T3 Water-water  Bottom: Temperature |
| --- | --- |
| 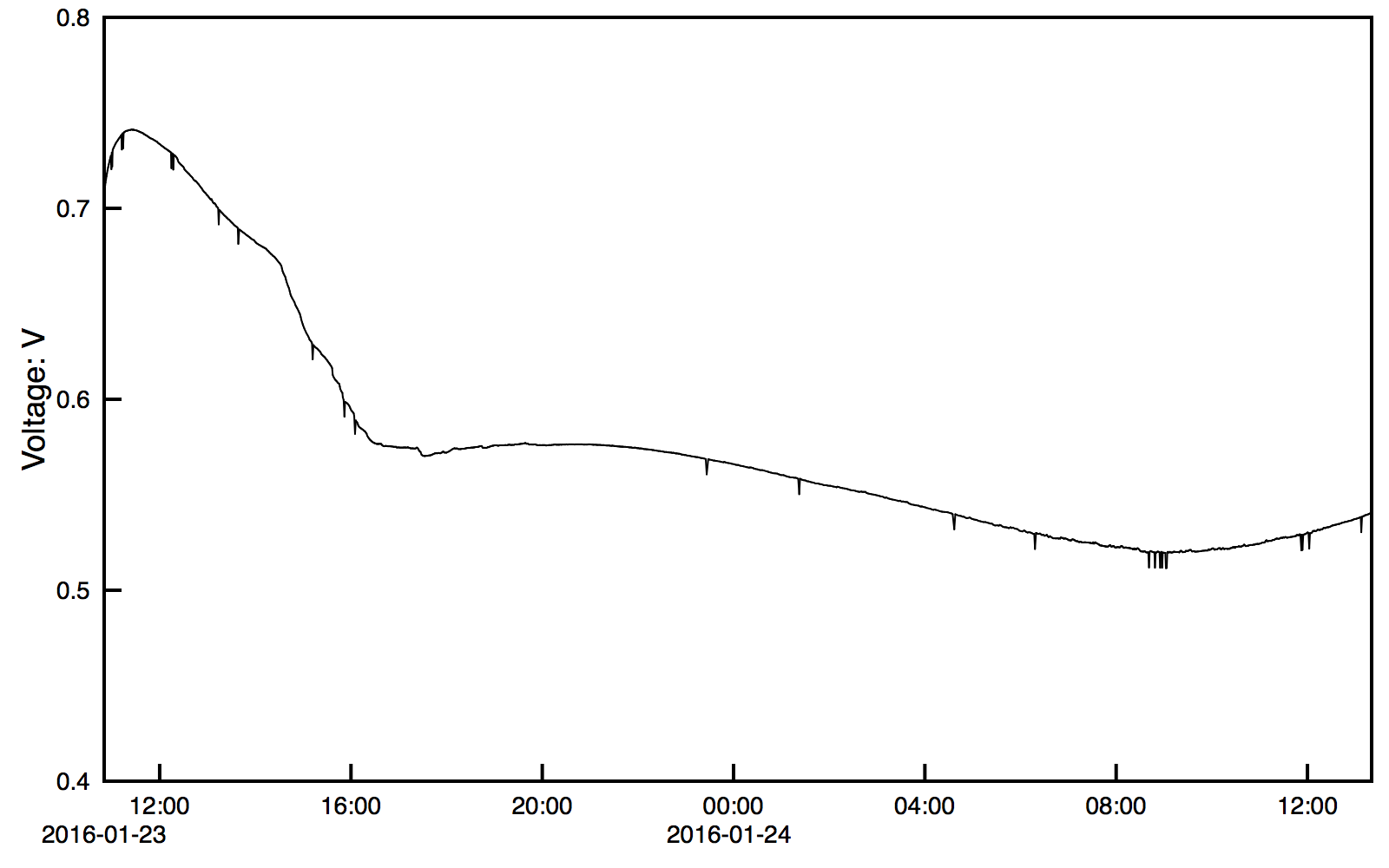 | 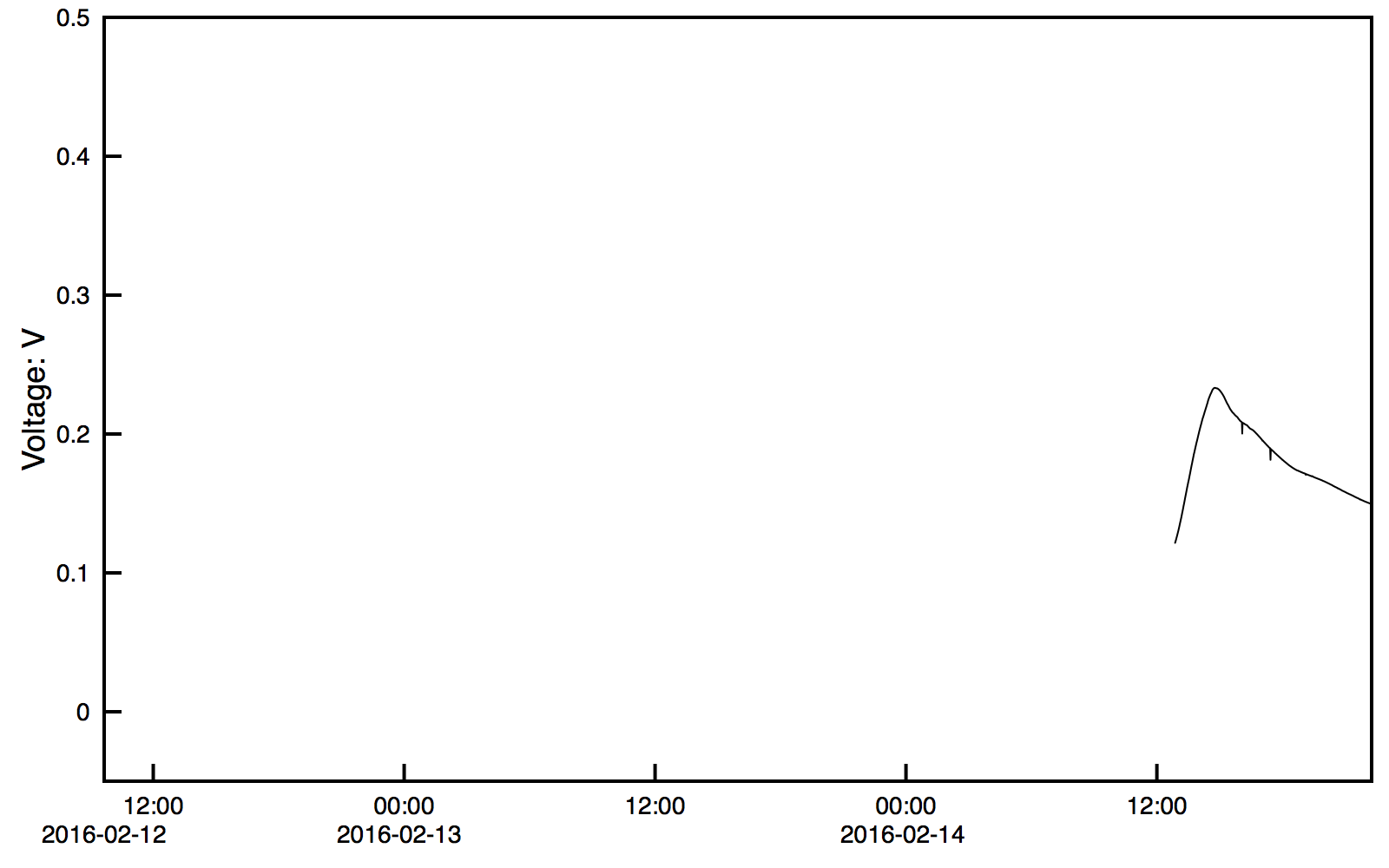 |
| 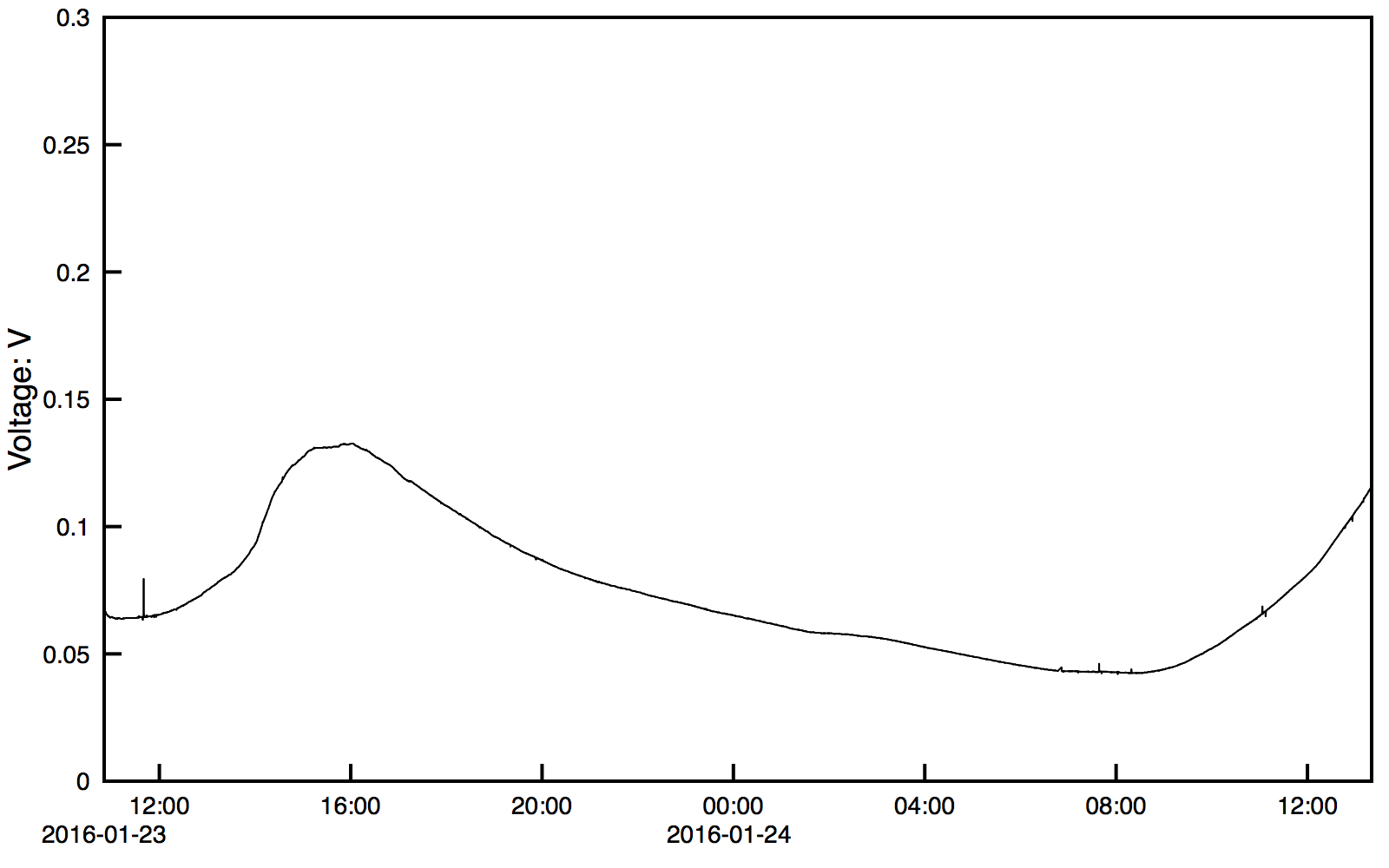 | 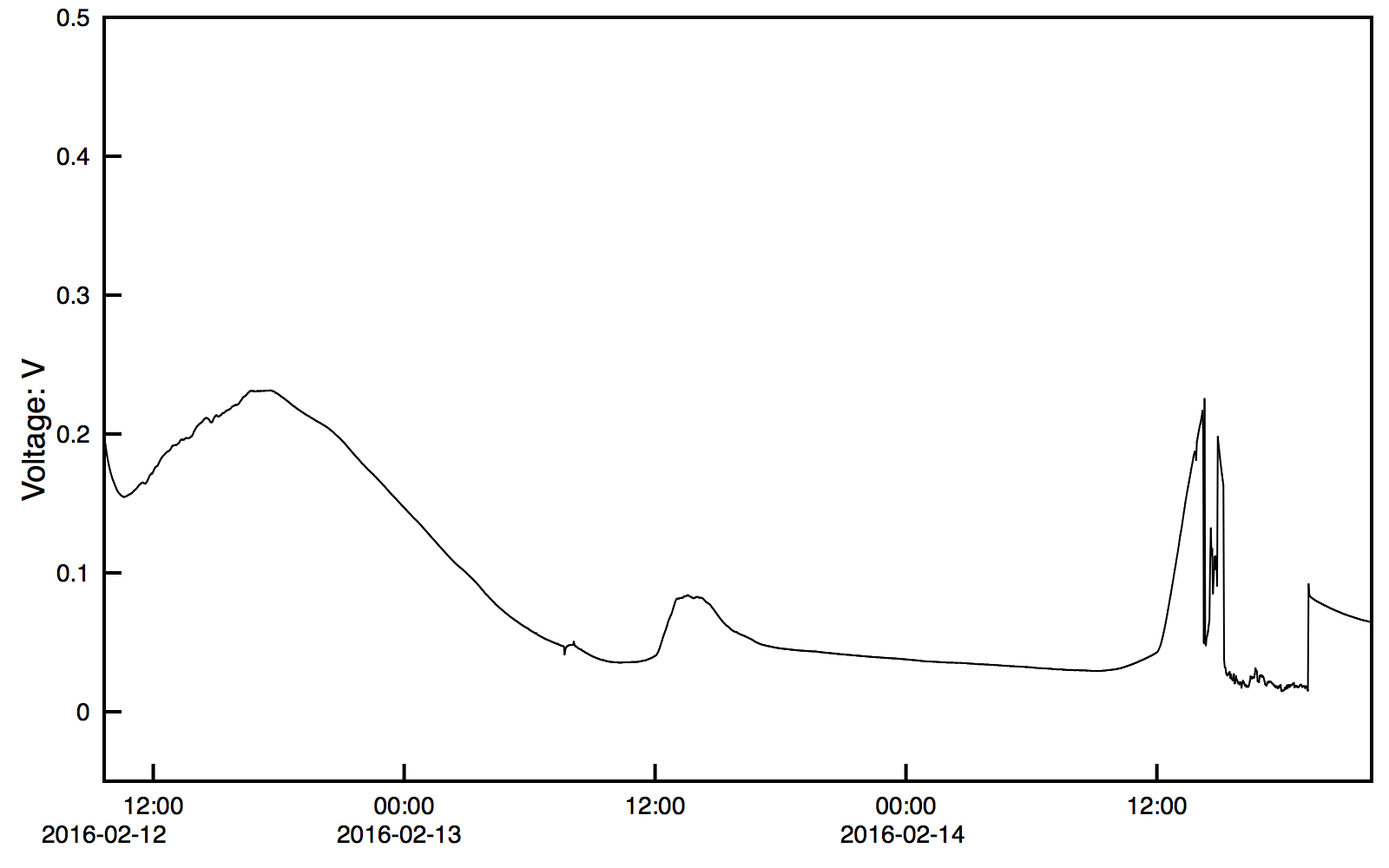 |
| 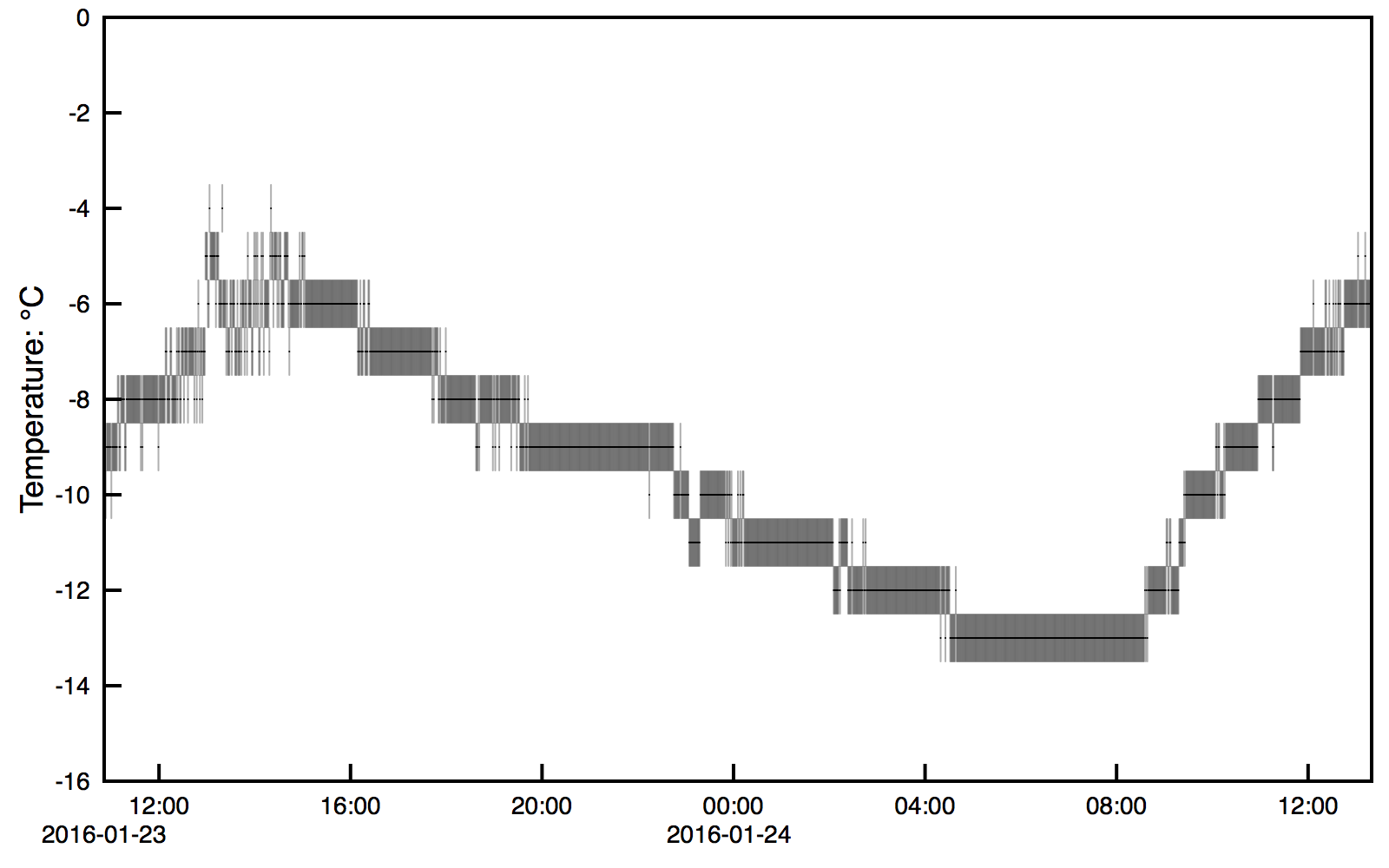 | 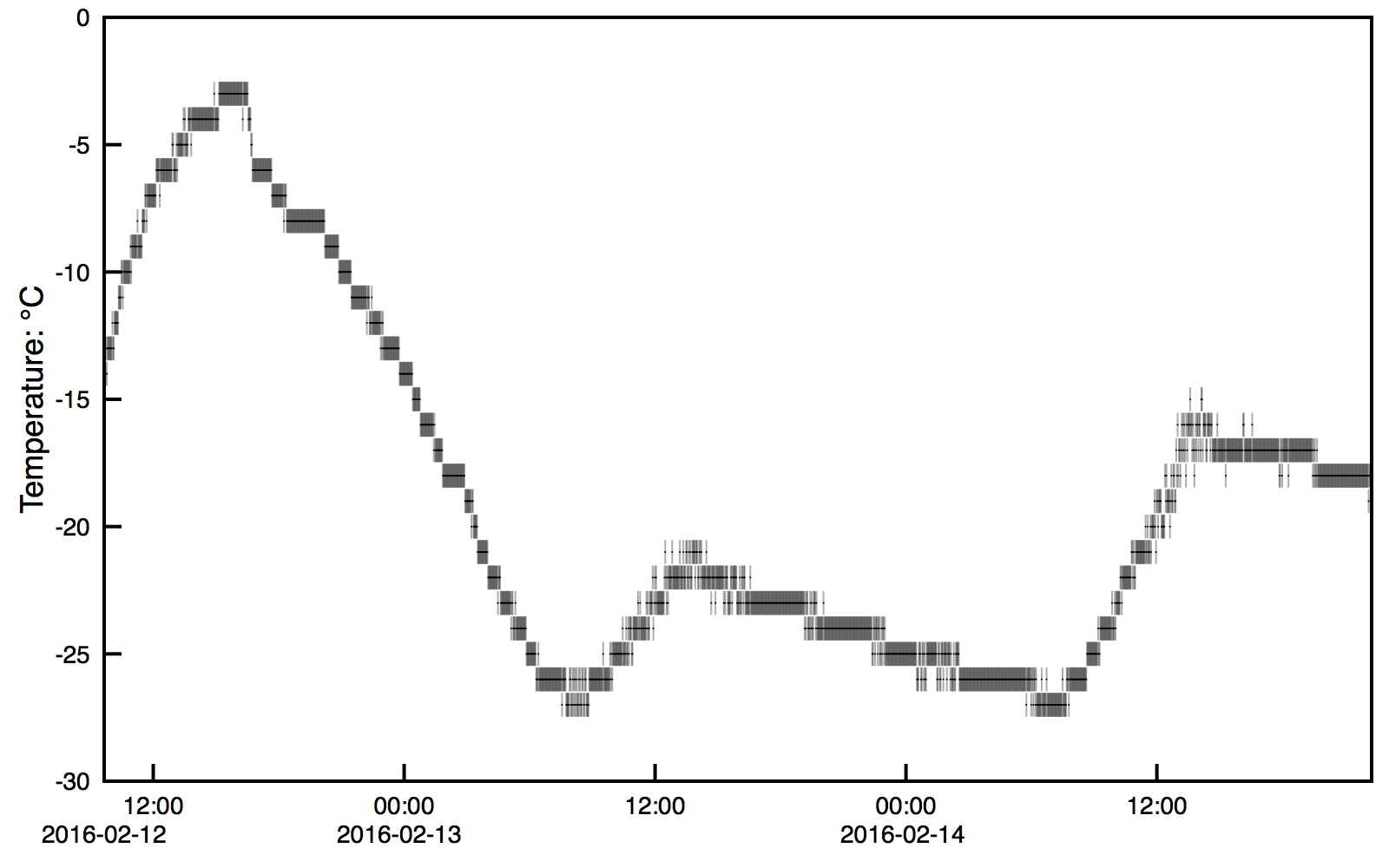 |

**Figure S20.** Correlation between prototype panel voltage and temperature in Year 1 Run 2 and Year 1 Run 4. Abbreviations: E = Experiment. T = Trial.

| Year 1 Run 1  Top: E1T1 water-water  Bottom: Temperature | Year 2 Run 1  Top: E5T1 Rochelle salt-water  Bottom: Temperature |
| --- | --- |
| 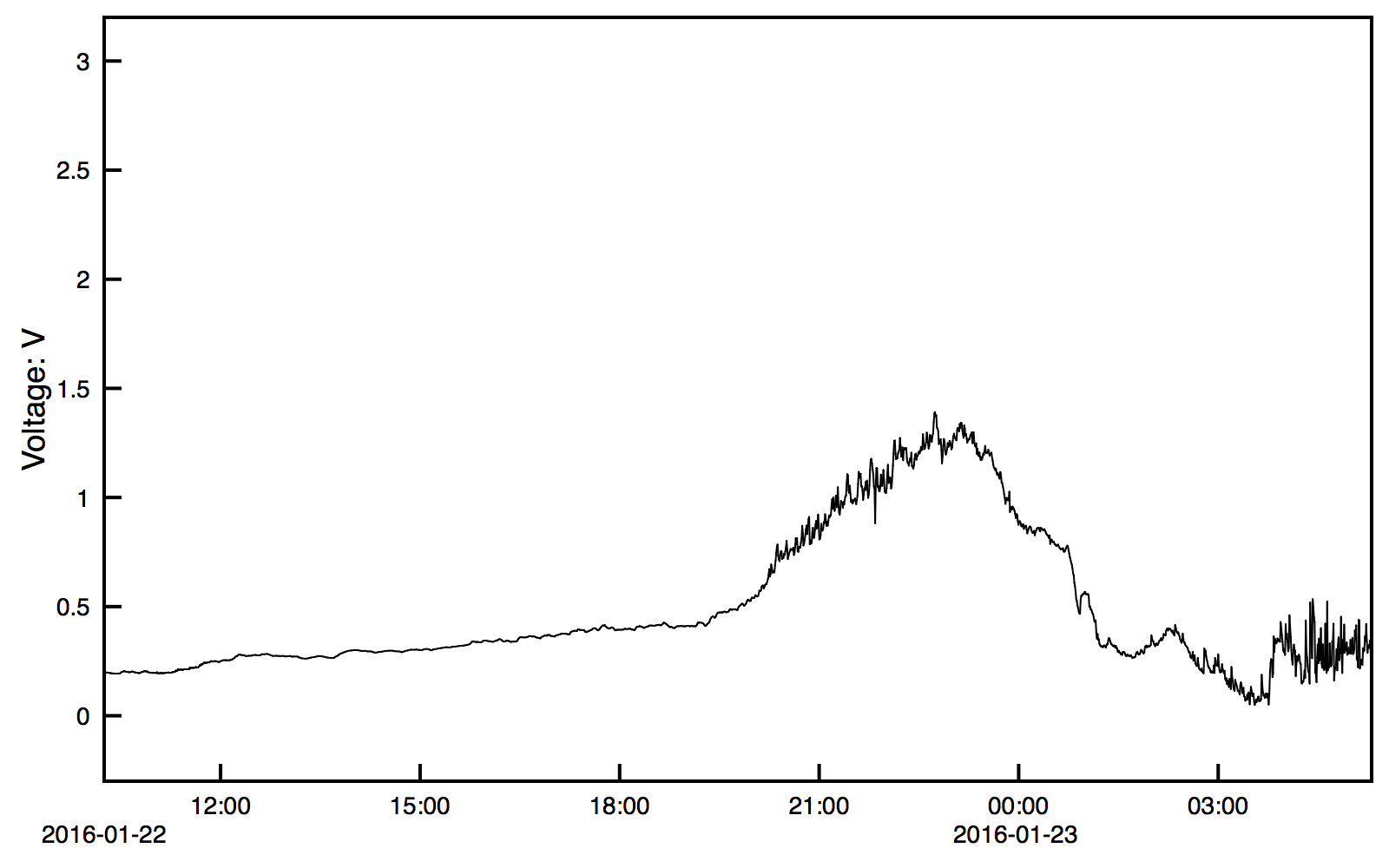 | 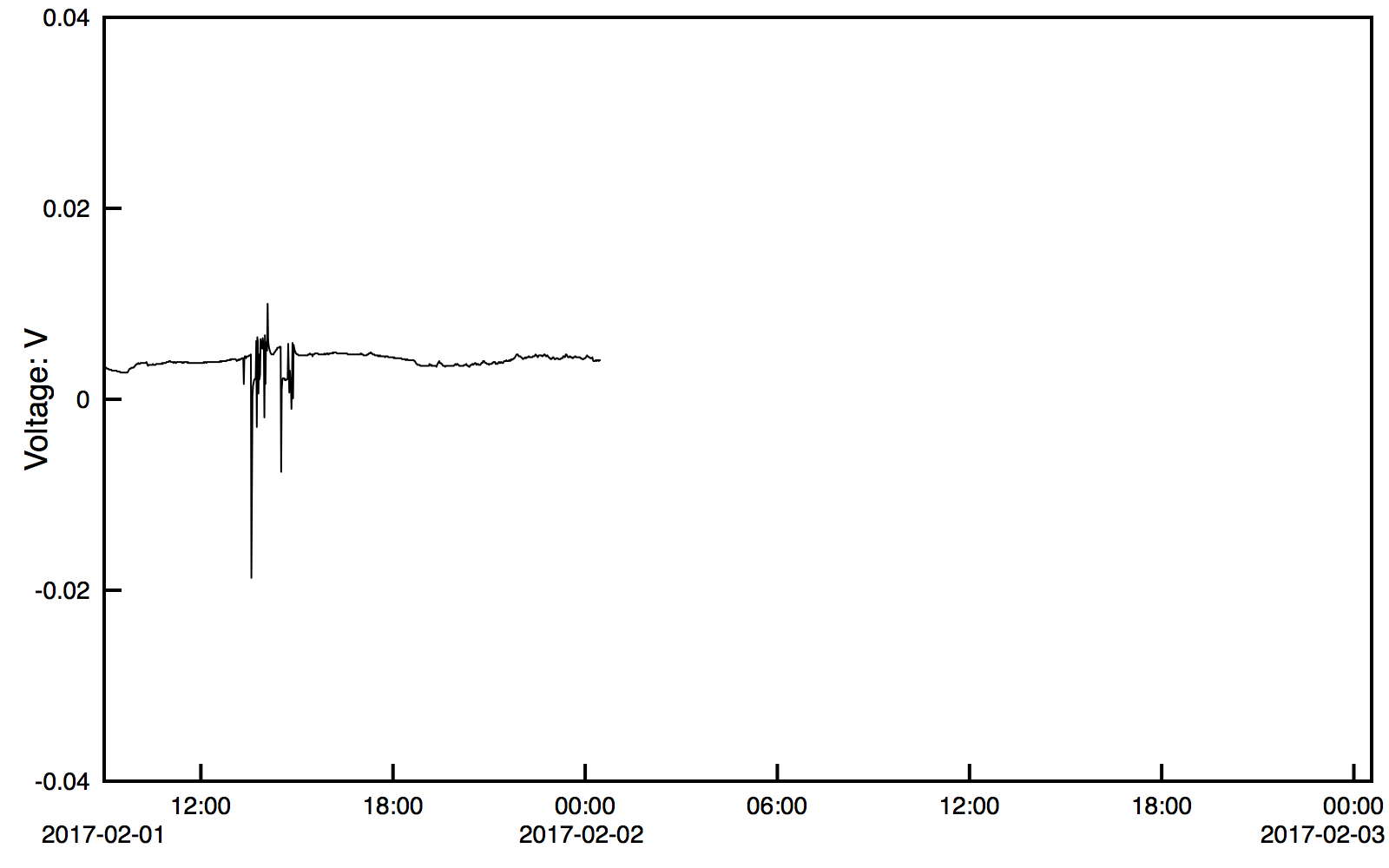 |
| 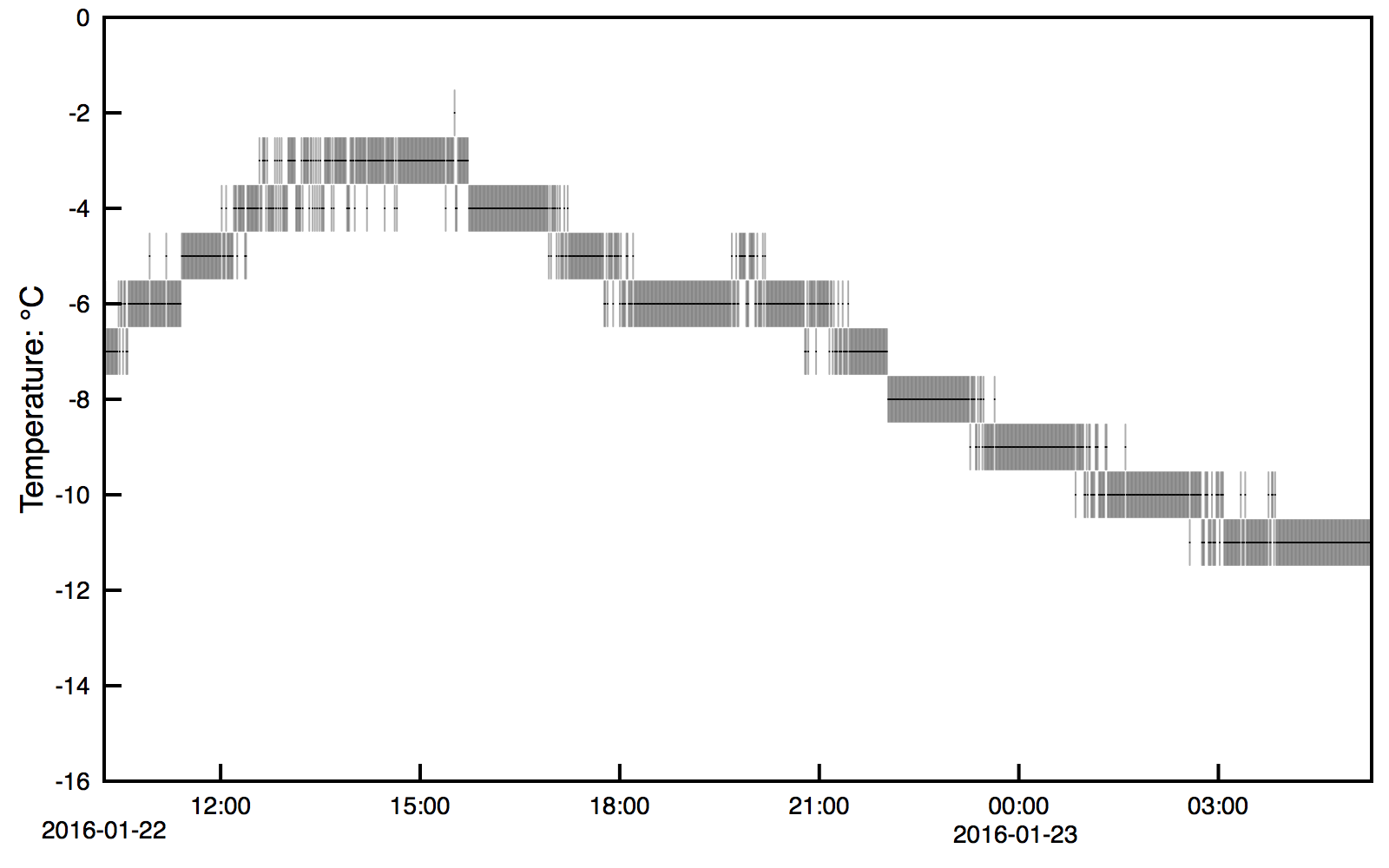 | 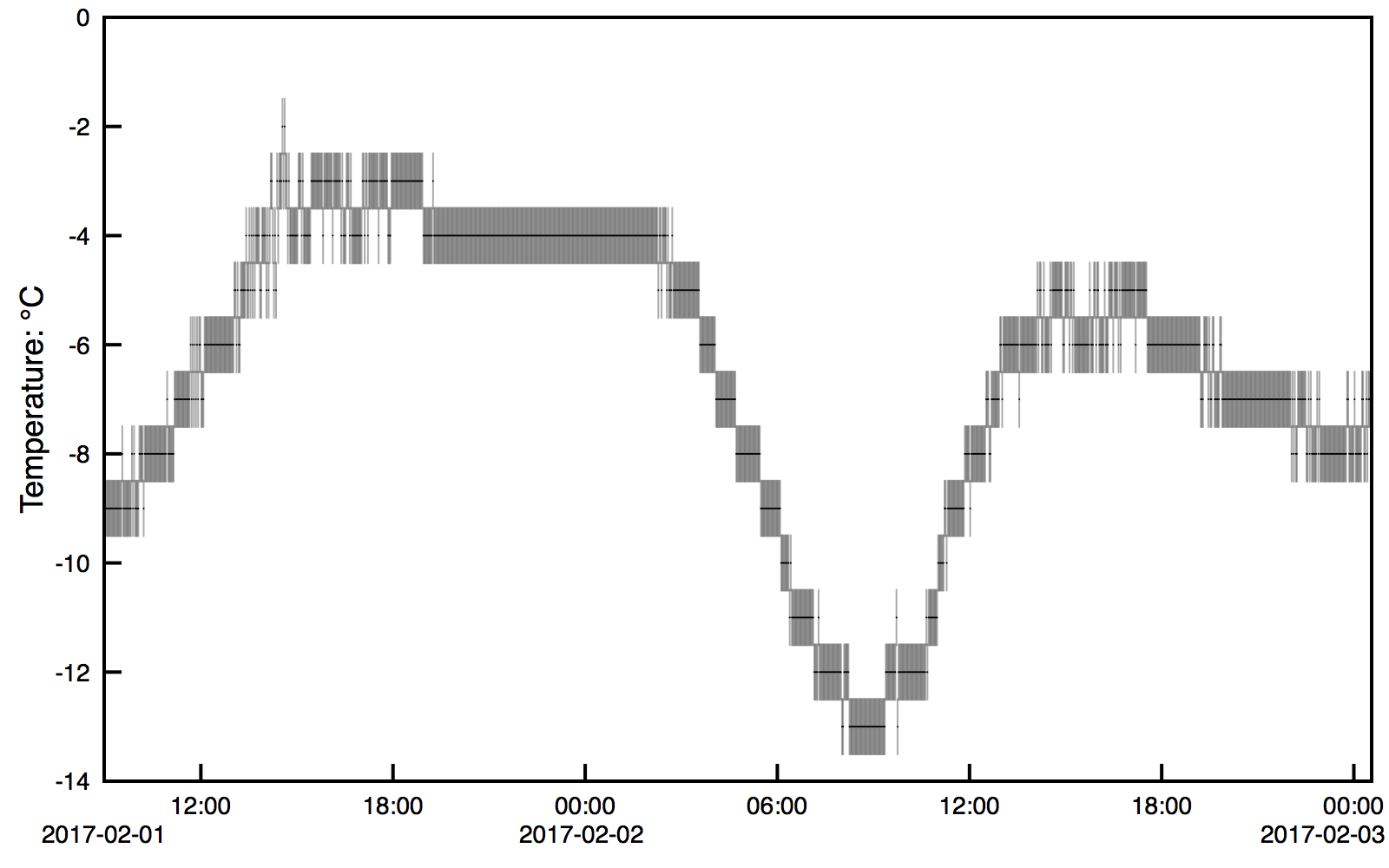 |

**Figure S21.** Prototype panel voltage and temperature. Note the onset of noise in the voltage trace and the corresponding temperature local high. Abbreviations: E = Experiment. T = Trial.

Ice formation is a dynamic process and changes in form during the trials were common. These included surface bulging, surface pitting or irregular surface texture, loss of transparency, exsolution of solute as a surface film whether as a crust or as a liquid. There were also ice nucleations preferential to the wires of the device and walls, plus regions of entrapped fluid and bubbles of fluid, as well as spatially organized bubble nucleations. Table S14 lists changes that occurred in each trial. Changes in panel form highlight the dynamic nature of ice, and its dependence on ambient energy in the system. Note also that typical pyroelectric values for ice are not known, but peak pyroelectric output of ferroelectric materials can be high during temperature changes, e.g. 50 V for barioperovskite at 109°C near its phase transition (Shiozaki et al., 2002). However, our experiments did not present evidence to elucidate active processes and mechanisms effectively beyond a qualitative judgement.

**Table S14.** Changes in Form Observed During Experimental Trials.

| **Phenomenon** | **E1T1** | **E1T2, E2** | **E1T3, E3T1** | **E3T2, E4** | **Y1R6** | **E5** | **E6** |
| --- | --- | --- | --- | --- | --- | --- | --- |
| Surface bulging | x | x | x |  |  |  |  |
| Surface pitting | x | x | x |  |  |  |  |
| Irregular surface |  |  |  |  |  |  | x |
| Transparency loss | x | x | x | x |  |  |  |
| Surface exsolution crust |  |  | x |  |  |  |  |
| Surface exsolution liquid |  |  |  |  | x | x |  |
| Ice on wires |  |  |  | x | x |  |  |
| Ice up walls |  |  |  |  | x | x |  |
| Entrapped fluid |  |  |  | x |  | x |  |
| Fluid bubbles | x |  |  |  | x |  |  |
| Spatially organized bubbles | x |  |  |  |  |  |  |

Note: Abbreviations: E = Experiment. T = Trial. Y = Year. R = Run. Y1R6 voltage data were not gathered correctly and not used.

Finally, one useful hypothesis related to power generation is that additives in the ice may absorb photons, and cause electron charge transfer. Kaolinite, for example, with a band gap of 4.52 eV, is the most photoelectric material present in our experiments, i.e. in Experiment 6. This was the most successful experiment, and is described in the main paper. The kaolinite may be responsible for the power production in an exposed panel. Our experiments did not find evidence to support or rule out this hypothesized ice additive photoelectric effect.

Section S3 Experimental Failures and Faults

There were faults with the computer logging and saving the data. These occurred four times out of twelve, i.e. 33% of the time, and resulted in data loss. Table S15 shows these, along with other equipment failures that affected the data. There were three faults noted with the DMMs. (1) One or both DMMs switched units for a few of the data points during three runs out of twelve during automatic logging, and these needed to be corrected *post hoc* by examining nearest neighbors, i.e. if the data was 100x their nearest neighbors, and the number of digits recorded was only two instead of four, these excursions in voltage were corrected by division by 100, e.g. 0.62 became 0.0062. The lack of two digits when four were typically recorded strongly suggested this correction. (2) During one run (E2), one DMM recorded a few excursions of a uniform magnitude (0.008 V) during automatic logging and these are also suspect but were retained. During measurement by hand, the DMMs performed as per their design, i.e. if a value was not stable, "Fail" would appear in the display, and the measurement needed to be taken again. (3) The batteries for both DMMs failed during Year 2 Run 1 (E5T1-2), resulting in some loss of data and ending the run earlier than planned.

Some differences between the first trial and all subsequent experiments and trials may be attributable to wear and oxidation that occurred during the first use of the wire mesh electrodes. These were notably oxidized during their first use. Changes between subsequent trials were not notable. Note also that the method of pouring the top layer liquid onto the frozen bottom layer resulted in fracture for nearly all of Experiments 1-4, i.e. all but E1T2 and E2. This necessarily led to inhomogeneities in the panels. Likewise, rainfall during Experiment 6 affected the surface of one of the panels (E6T2) and led to inhomogeneities.

**Table S15.** List of Equipment Failures Affecting the Data.

| **Run** | **Panel A** | **Panel B** | **Notes** |
| --- | --- | --- | --- |
| Year 1 Run 1 | Computer froze while saving data. Total loss of data. | ***Experiment 1 Trial 1*** | Fracture of bottom layer during fabrication. Also, voltages may have been higher for this run compared to all subsequent runs, as the first use of the wire mesh electrodes created oxidation. |
| Year 1 Run 2 | ***Experiment 2***  DMM recorded several intervals of voltage excursions all of the same magnitude (0.008 V). (retained) | ***Experiment 1 Trial 2*** |  |
| Year 1 Run 3 |  |  | Run abandoned due to warm weather. |
| Year 1 Run 4 | ***Experiment 3 Trial 1***  Computer froze while saving data. Loss of 82% of data.  DMM recorded 7 data points with magnitudes exactly x100 of nearest neighbors value. (corrected) | ***Experiment 1 Trial 3*** | Fracture of bottom layer during fabrication. |
| Year 1 Run 5 | ***Experiment 3 Trial 2*** | ***Experiment 4***  Computer froze while saving data. Loss of 12% of data. | Fracture of bottom layer during fabrication. |
| Year 1 Run 6 |  |  | Mistake in circuit design of meter connections. No valid data. |
| Year 2 Run 1 | ***Experiment 5 Trial 1***  DMM recorded 17 data points with magnitudes exactly x100 of nearest neighbors value. (corrected)  DMM battery failed. Loss of 61% of data. | ***Experiment 5 Trial 2***  DMM battery failed. Ended trial early. |  |
| Year 2  Run 2 | ***Experiment 6 Trial 1***  DMM recorded 224 data points with magnitudes exactly x100 of nearest neighbors value. (corrected) | ***Experiment 6 Trial 2***  Computer froze while saving data. Loss of 51% of data. | Rainfall affected the surface of E6T2. |

Section S4 Geomagnetic Correlation with Sunrise and Sunset

This is included for interest. Note as general knowledge: some low values in the total geomagnetic field correspond with the onset of dusk, but this is not a one-to-one correlation. See Figure S20 for some examples. Globally, low values in the geomagnetic field also correspond (to some extent) with periods of increased terrestrial risk from solar activity and space weather (Menvielle and Marchaudon, 2007).

| Year 1 Run 1  Top: Total geomagnetic field  Bottom: Lunisolar data | Year 2 Run 1  Top: Total geomagnetic field  Bottom: Lunisolar data | Year 2 Run 1  Top: Total geomagnetic field  Bottom: Lunisolar data | Year 2 Run 2  Top: Total geomagnetic field  Bottom: Lunisolar data |
| --- | --- | --- | --- |
| 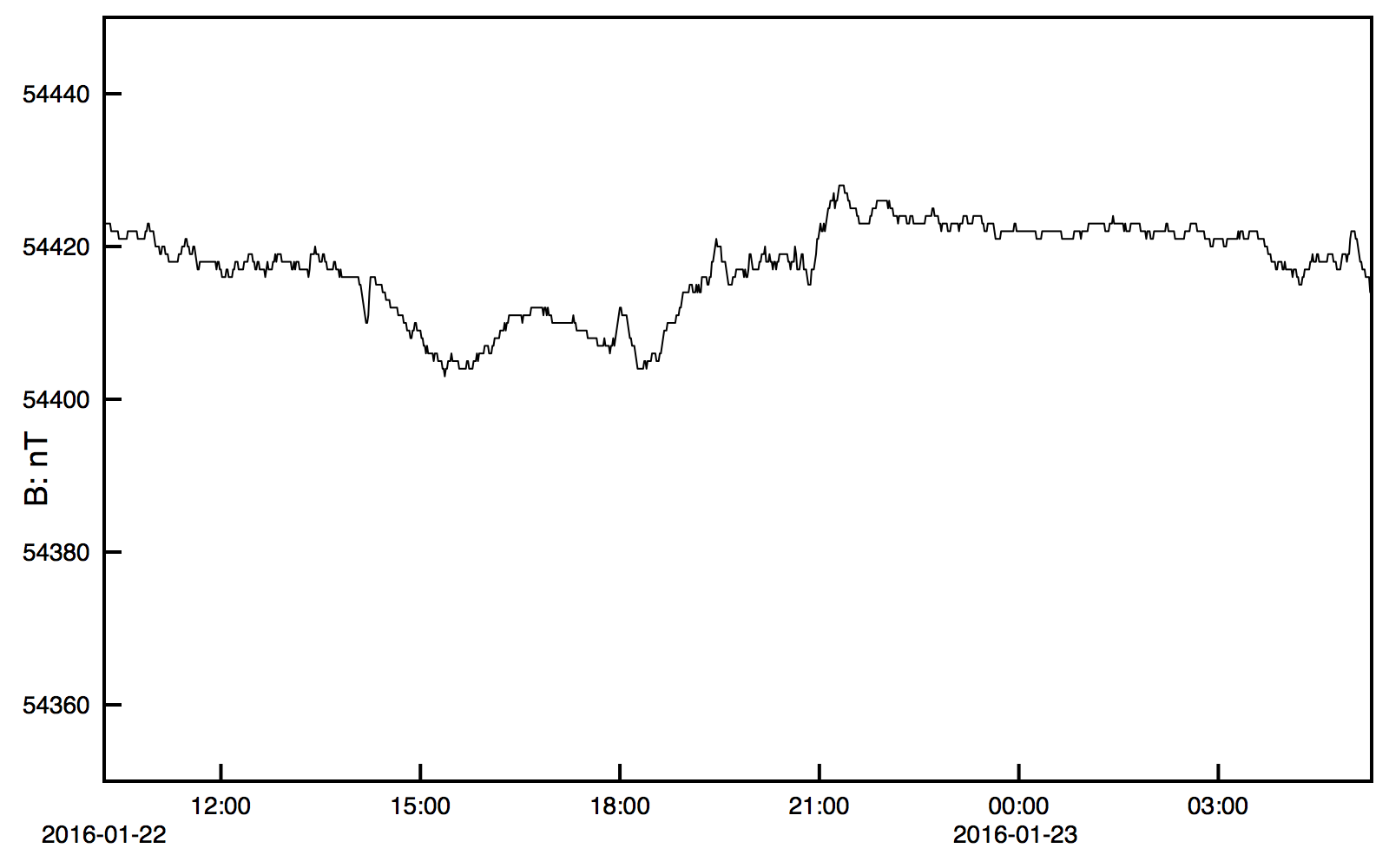 | 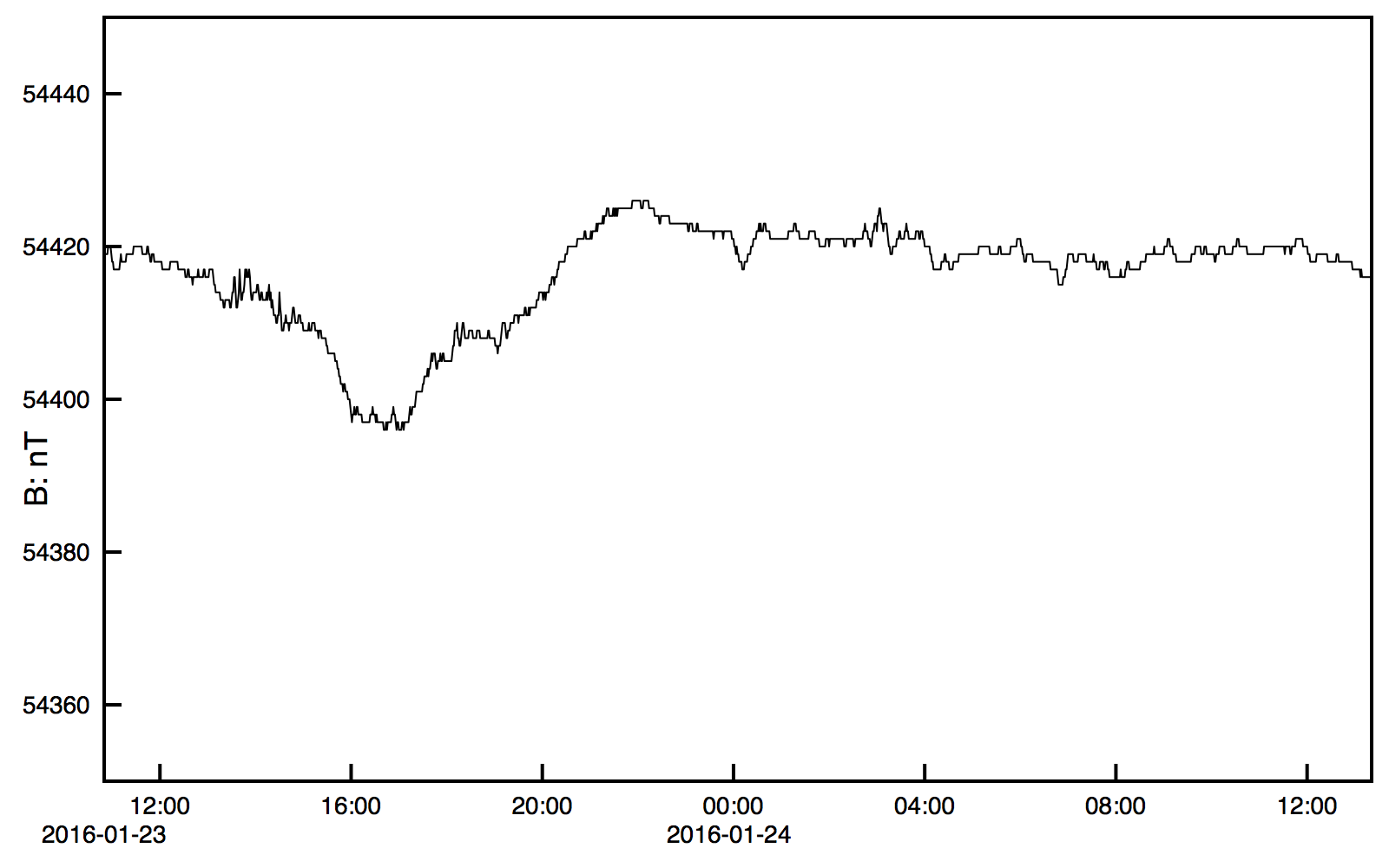 | 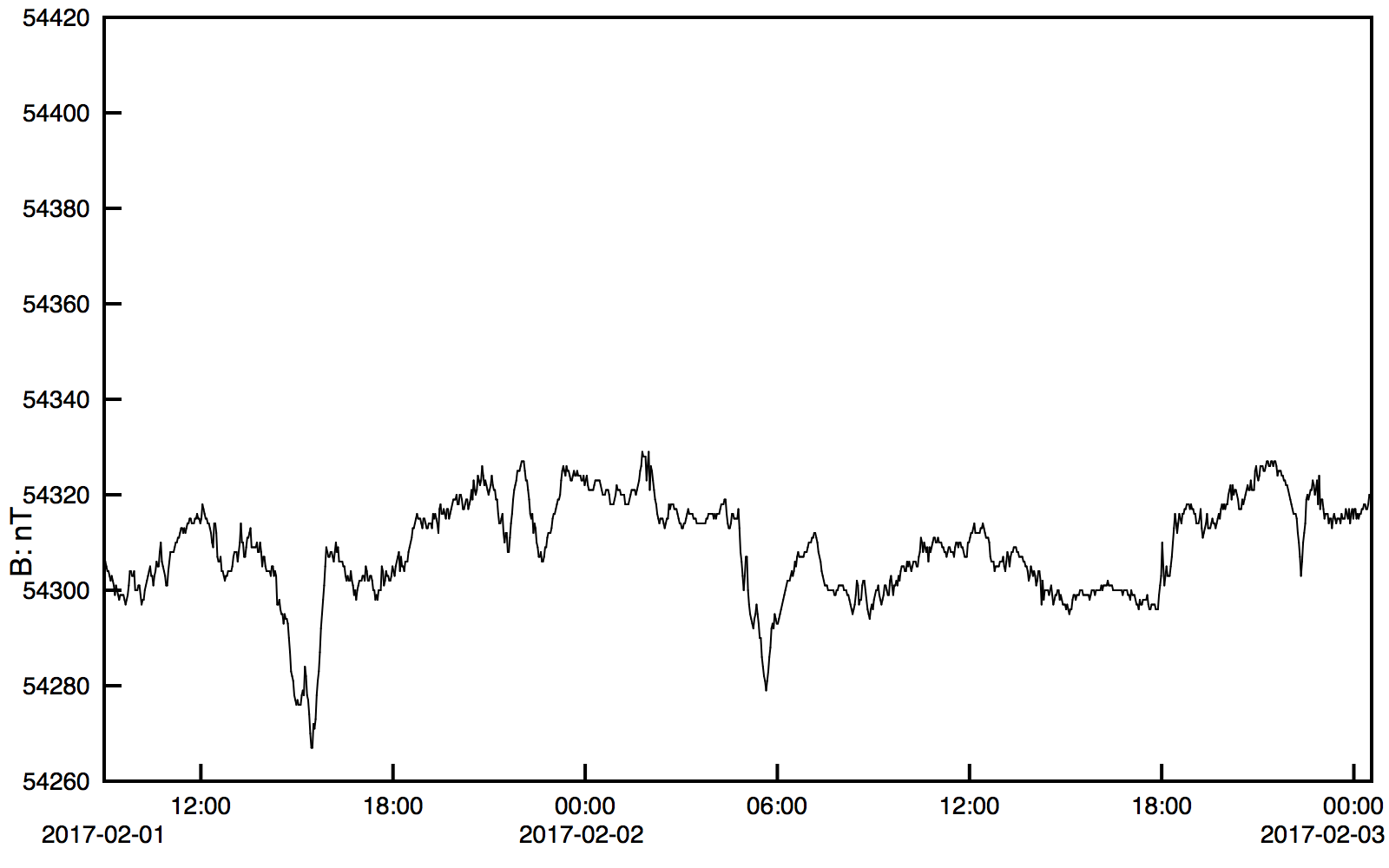 | 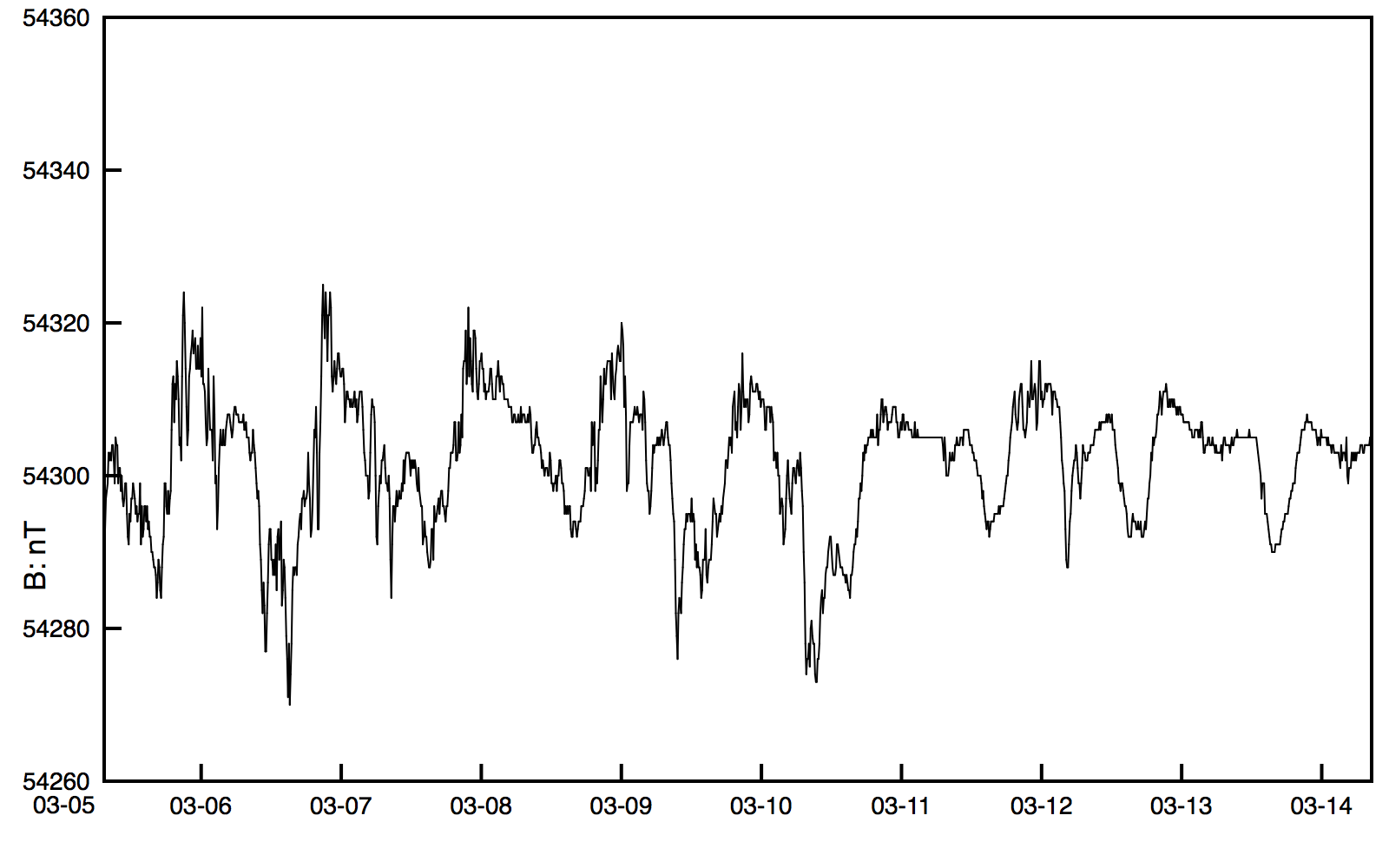 |
| 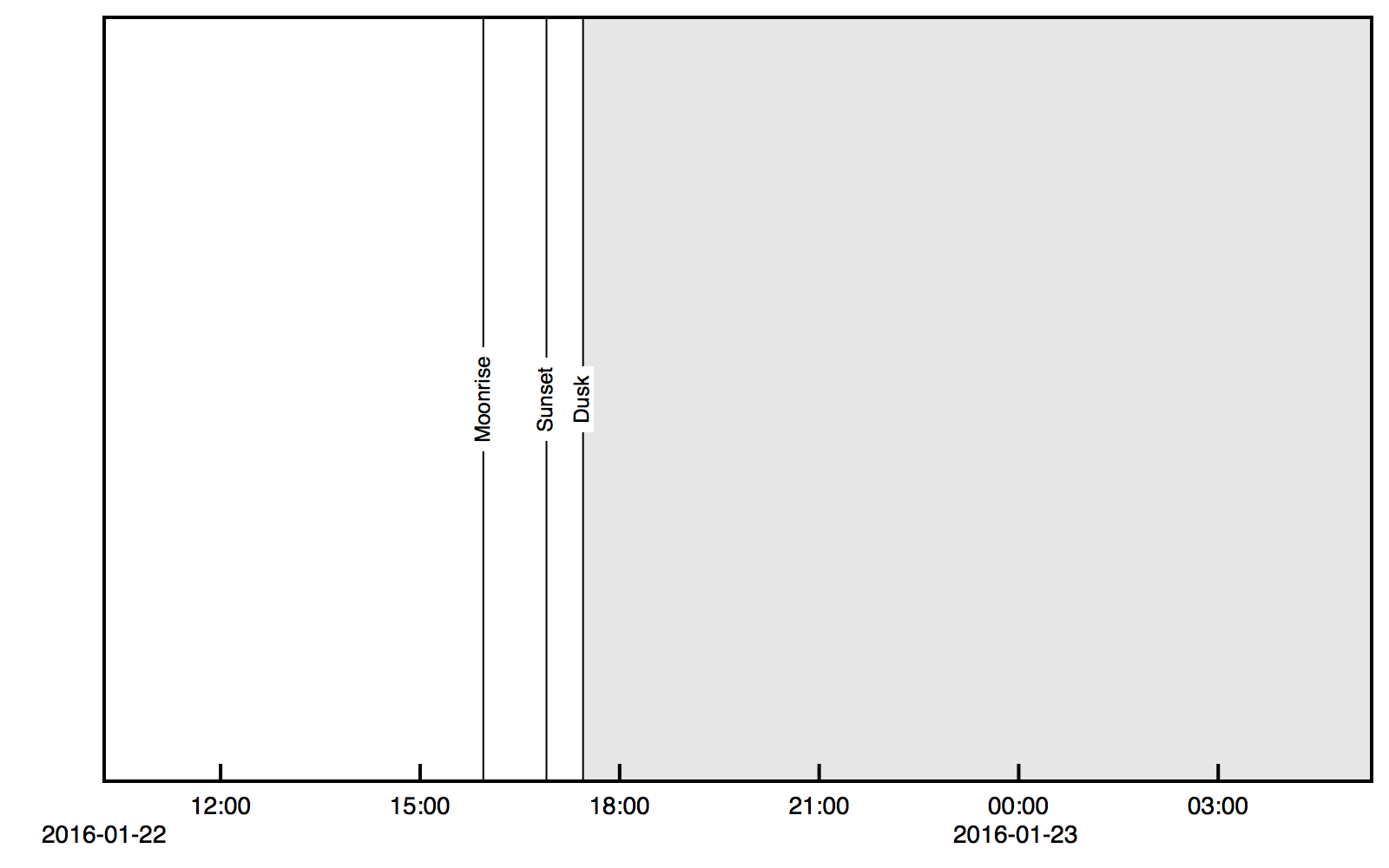 | 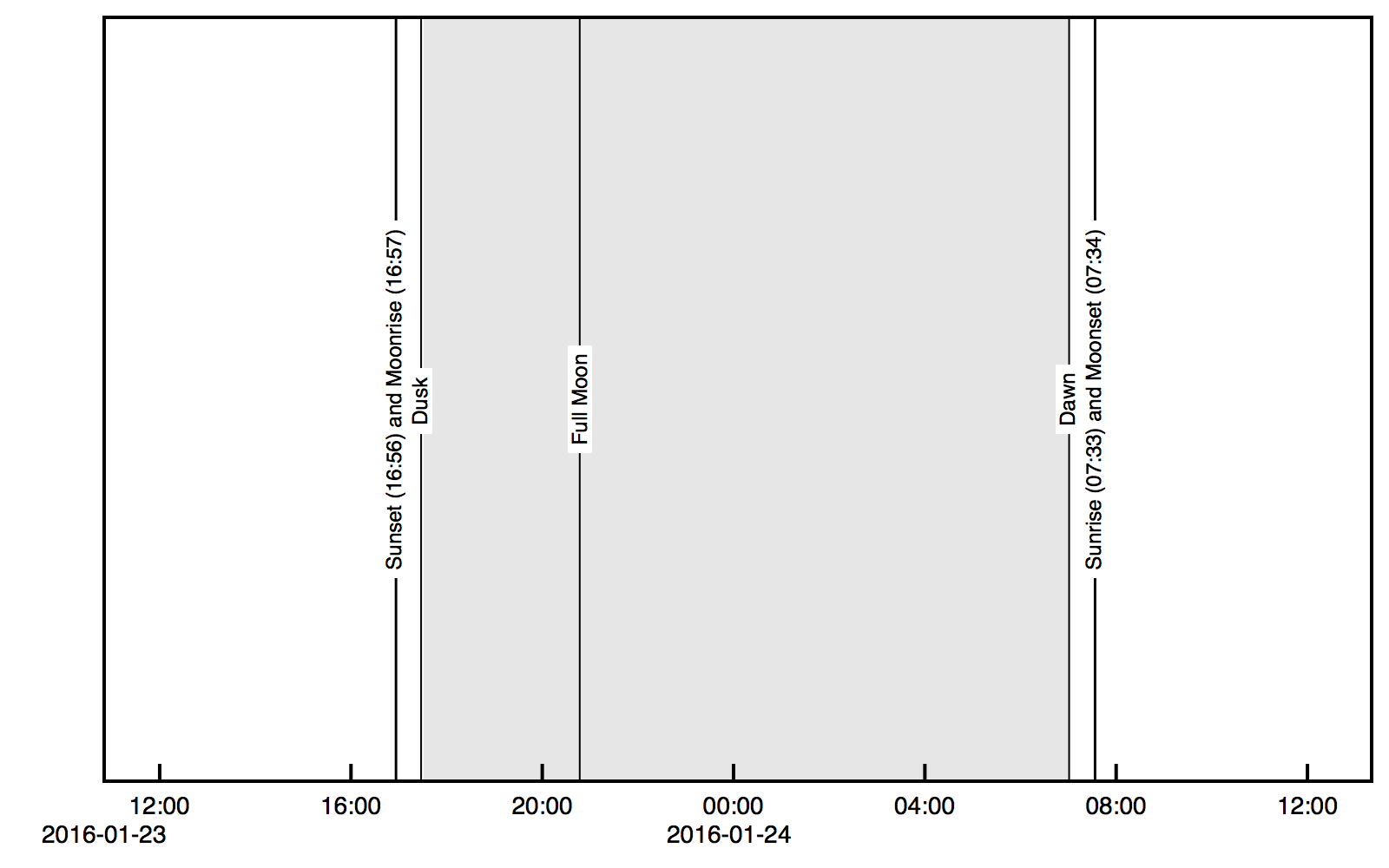 | 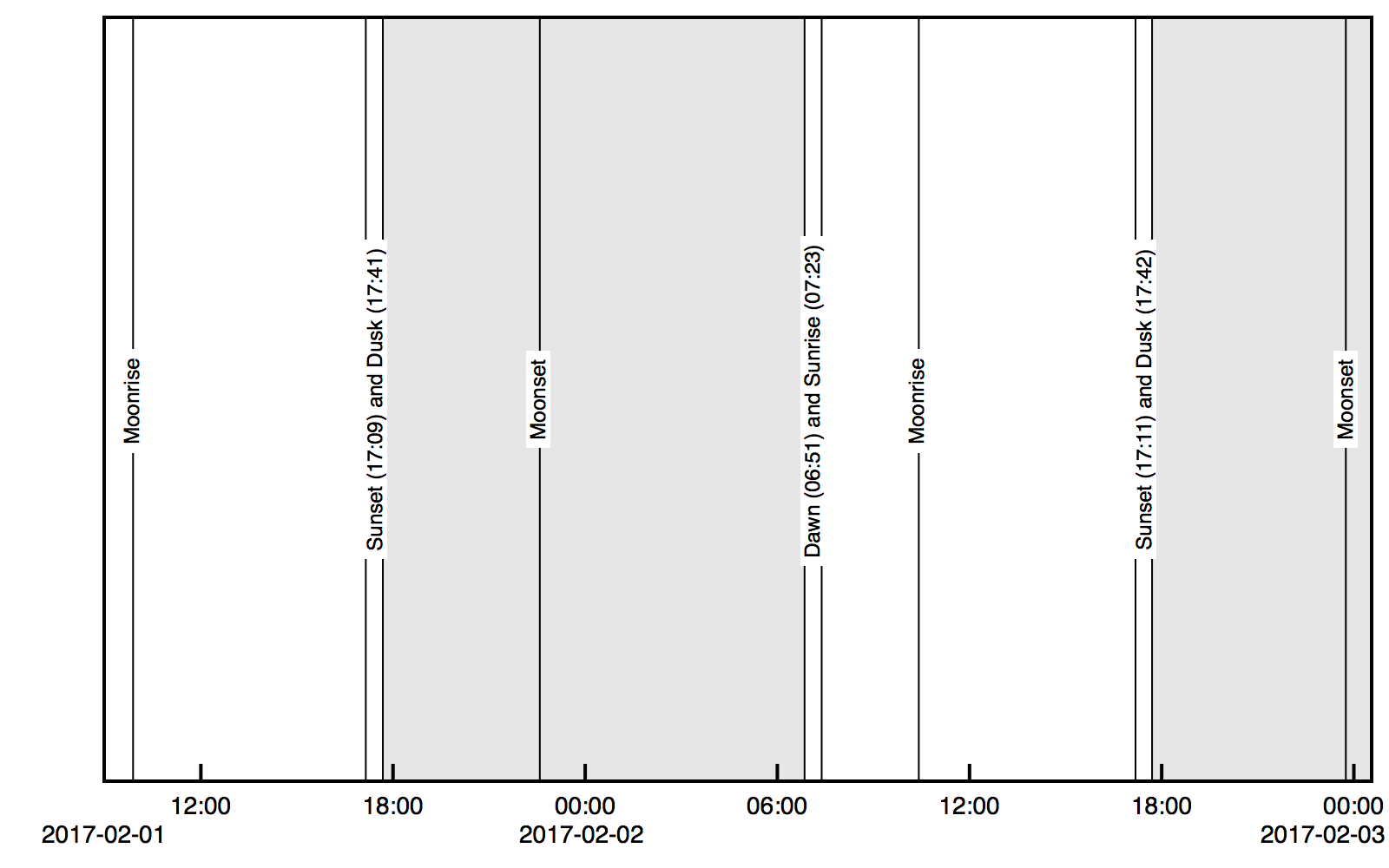 | 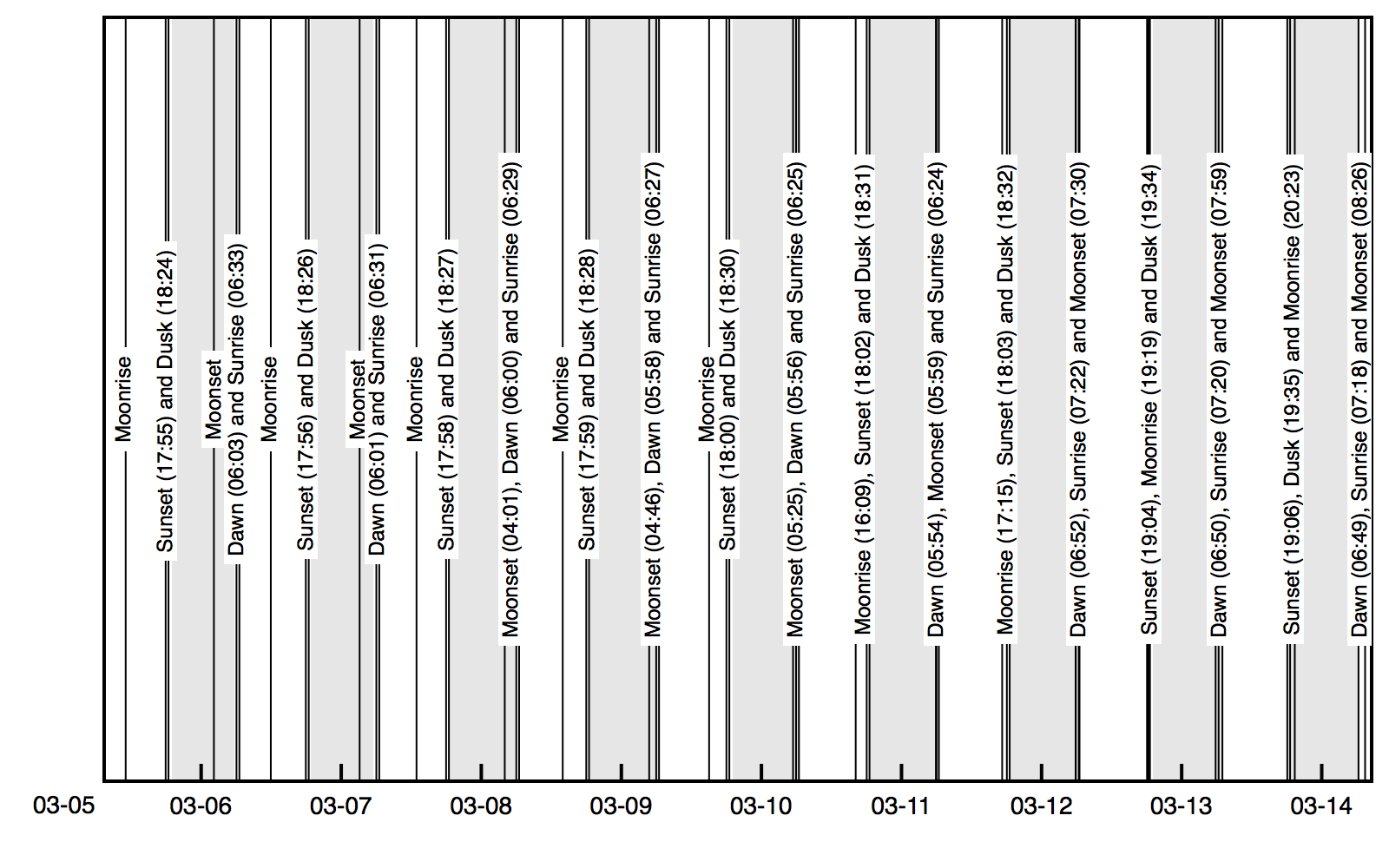 |

**Figure S20.** Comparison of total geomagnetic field and the onset of dusk in the study location during the first two runs of each experimental year. Note that the correspondence between the onset of dusk and a low field value is not one-to-one.

Section S5 Astrobiology and the Origin of Life

A proton pump is a protein-based structure that aids the transfer of protons across a membrane in living systems. It allows for a pH gradient across the membrane that can be used for biological processes.There are a number of different mechanisms/types of proton pumps, and these have evolved several times within living organisms independently (Nelson et al., 1992).

The experiments described in this paper have looked at a few cases of how proton transfer and pH work in icy systems. To think more broadly: Water ice is not present only in terrestrial environments. There are different types of icy systems in the Solar System, including comets, asteroids, planetary satellites (such as Europa, Ganymede, Callisto, Mimas, Enceladus, Titan, Miranda, Umbriel, Triton, etc.), dwarf planets (such as Pluto and Ceres) and planets such as Earth and Mars. Note that not all of these "icy worlds" are replete with water ice. There are a variety of different chemistries (e.g. methane on Pluto) and other features present, including cryovolcanoes, subsurface bodies of water, deformation from tidal forces, and even unique small structures such as brinicles (Vance et al., 2019). Some of these icy worlds include protobiological chemistries, for example, amino acids on the Murchison meteorite have been studied for decades (Kvenvolden et al., 1970).

Earth is one of many icy worlds. There is evidence to support the hypothesis that Earth was completely covered in ice in the past during the Neoproterozoic Era (Schrag and Hoffman, 2001) or that evidence of glaciation, for some unknown reason, is preferentially preserved from the Neoproterozoic (Etienne et al., 2007). Looking backwards at stellar evolution, the early Sun produced insufficient energy to create conditions of habitability (liquid water) on early Earth, in what is known as the "Faint Young Sun Paradox" (Obridko et al. 2020). Yet life is here.

Metabolism, heredity and membranes are all features of living organisms whose origin is not well understood, but progress is being made to see how environmental trends may influence their development (Takagi et al., 2020). For example, an environment with abundant energy is less likely to produce membranes and heredity, as exogenous chemical cycles are stable already. Lack of environmental energy produces pressure for membranes and heredity.

Although RNA can catalyze its own polymerization, the conditions for RNA to act as an autocatalyst do not work easily in aqueous environments, such as mid ocean ridge vent systems (Goodman and Lenferink, 2012) or geothermal fields (Damer and Deamer, 2020). To act as an autocatalyst to produce polymerization requires a high concentration of RNA. In water, the concentration doesn't occur because of dilution. But in ice, RNA may be isolated enough to act as an autocatalyst and produce RNA polymeric concentrations of note similar to those from nanoconfinement (Trixler, 2021). Thus modest production of electricity via the action of pH gradients in ice may be important for studies about the origin of life in icy worlds, as they provide a pathway for abiogenesis. This work may form the basis for further study related to protometabolic processes, and especially regular, cyclical systems such as tidal force piezoelectricity, and also autocatalytic systems in ice generally.

References

Damer, B., & Deamer, D. (2020). The hot spring hypothesis for an origin of life. *Astrobiology, 20*(4), 429-452. doi:10.1089/ast.2019.2045

Etienne, J. L., Allen, P. A., Rieu, R., Le Guerroué, E., & Hambrey, M. J. (2007). Neoproterozoic glaciated basins: a critical review of the Snowball Earth hypothesis by comparison with Phanerozoic glaciations. In *Special Publication-International Association of Sedimentologists, 39*, 343. doi:10.1002/9781444304435.ch19

Goodman, J. C., & Lenferink, E. (2012). Numerical simulations of marine hydrothermal plumes for Europa and other icy worlds. *Icarus, 221*(2), 970-983. doi:10.1016/j.icarus.2012.08.027

Kvenvolden, K., Lawless, J., Pering, K., Peterson, E., Flores, J., Ponnamperuma, C., ... & Moore, C. (1970). Evidence for extraterrestrial amino-acids and hydrocarbons in the Murchison meteorite. Nature, 228(5275), 923-926. doi:10.1038/228923a0

Menvielle, M., & Marchaudon, A. (2007). Geomagnetic indices in solar-terrestrial physics and space weather. In Space Weather (pp. 277-288). Springer, Dordrecht. doi:10.1007/1-4020-5446-7_24

Nelson, N., Beltrán, C., Supek, F., & Nelson, H. (1992). Cell biology and evolution of proton pumps. Cellular Physiology and Biochemistry, 2(3-4), 150-158. doi:10.1159/000154637

Obridko, V. N., Ragulskaya, M. V., & Khramova, E. G. (2020). Young Sun, galactic processes, and origin of life. *Journal of Atmospheric and Solar-Terrestrial Physics, 208*, 105395. doi:10.1016/j.jastp.2020.105395

Schrag, D. P., & Hoffman, P. F. (2001). Life, geology and snowball Earth. Nature, 409(6818), 306-306. doi:10.1038/35053170

Shiozaki Y., Nakamura E., Mitsui T. (2002). Electrical Properties: Ferroelectrics and Related Substances: Perovskite-type Oxides and LiNbO_3_ Family. In: *Landolt-Börnstein Numerical Data and Functional Relationships in Science and Technology, New Series, Group III: Condensed Matter*, Volume 36a1. (ed): O. Madelung, Berlin, Springer-Verlag, 588 pp.

Takagi, Y. A., Nguyen, D. H., Wexler, T. B., & Goldman, A. D. (2020). The coevolution of cellularity and metabolism following the origin of life. *Journal of Molecular Evolution, 88*(7), 598-617. doi:10.1007/s00239-020-09961-1

Trixler, F. (2021). The origin of nucleic acids. In A. Neubeck, S. McMahon (Eds.), *Prebiotic Chemistry and the Origin of Life*. Springer Nature. Retrieved from http://www.nano.geo.uni-muenchen.de/publications.htm

Vance, S. D., Barge, L. M., Cardoso, S. S., & Cartwright, J. H. (2019). Self-assembling ice membranes on Europa: Brinicle properties, field examples, and possible energetic systems in icy ocean worlds. Astrobiology, 19(5), 685-695. doi:10.1089/ast.2018.1826
